# Phase II clinical trial of nab‐paclitaxel plus cisplatin plus gemcitabine (NABPLAGEM) in patients with untreated advanced pancreatic cancer

**DOI:** 10.1002/cam4.7412

**Published:** 2024-06-22

**Authors:** Gayle S. Jameson, Peter J. Hosein, Erin Pierce, Mandana Kamgar, Michael S. Gordon, Courtney Snyder, Denise J. Roe, Betsy C. Wertheim, Marina Davey, Michael T. Barrett, Daniel D. Von Hoff, Erkut Borazanci

**Affiliations:** ^1^ HonorHealth Research Institute Scottsdale Arizona USA; ^2^ Translational Genomics Research Institute, (TGen) a part of City of Hope Phoenix Arizona USA; ^3^ University of Miami Miami Florida USA; ^4^ Ochsner Health New Orleans Louisiana USA; ^5^ Medical College of Wisconsin Milwaukee Wisconsin USA; ^6^ The University of Arizona Cancer Center Tucson Arizona USA; ^7^ Mayo Clinic Scottsdale Arizona USA

**Keywords:** clinical trial, nab‐paclitaxel + cisplatin + gemcitabine, pancreatic adenocarcinoma, phase II, untreated metastatic pancreatic adenocarcinoma

## Abstract

**Background:**

A previous phase IB/II study of nab‐paclitaxel + cisplatin + gemcitabine (NABPLAGEM) in 25 patients with previously untreated metastatic pancreatic ductal adenocarcinoma (PDAC) demonstrated favorable results. This phase II study was conducted to further evaluate the safety, efficacy, and impact on quality of life (QOL) of NABPLAGEM in a multi‐center setting.

**Methods:**

Participants were ≥18 years; had measurable PDAC; Karnofsky performance status of ≥70%; life expectancy ≥12 weeks; <grade 2 pre‐existing peripheral neuropathy, and adequate hematologic, hepatic, and renal function. Study treatment included nab‐paclitaxel (125 mg/m^2^), cisplatin (25 mg/m^2^), and gemcitabine (1000 mg/m^2^), on Days 1 and 8 of a 21‐day cycle. The primary endpoint was 12‐month overall survival (OS).

**Results:**

A total of 42 patients were enrolled with a median age of 66.8 years, a majority were male (66.7%) and white (76.2%). Treatment‐related adverse events ≥grade 3 were: thrombocytopenia (64.3%), anemia (47.6%), and neutropenia (26.1%). OS at 12 months was 38.1%, with 12% of patients alive as of last contact (20–40+ months). In 37 evaluable participants responses included: partial response (40.5%), stable disease (43.2%), and progressive disease (16.2%), with overall response rate of 40.5% and DCR of 88.6%. Statistically significant improvement in QOL was reported during the first three cycles.

**Conclusion:**

The primary objective of achieving a 12‐month survival comparable to the previous phase Ib/II NABPLAGEM PCRT 12‐001 was not achieved in this trial. NABPLAGEM has efficacy in advanced PDAC, demonstrating early disease control noted by improvement in patient symptoms, decrease in tumor volume and tumor markers within the first 9 weeks. The activity and tolerability make this a suitable regimen for further testing in the neoadjuvant setting.

## 1 INTRODUCTION

Pancreatic cancer is the third leading cause of cancer deaths in the United States, accounting for 8% of all cancer deaths and 3% of cancer cases. In 2023, 64,050 new cases and 50,550 deaths are expected.^1^

The majority of pancreatic ductal adenocarcinoma (PDAC) patients present with locally advanced or metastatic disease, resulting in a 5‐year survival rate of 11%, up from 7.8% in 2008.^1^, ^2^, ^3^ Treatment options for patients with inoperable, locally advanced, or metastatic disease remain limited and offer only modest improvements in overall survival (OS).

Greater insight into the biology of PDAC has prompted new treatment strategies. Pancreatic tumors possess numerous molecular signatures including DNA repair deficiencies and are potentially vulnerable to new targeted therapies and DNA damaging agents.^4^, ^5^ We previously presented our whole genome/transcriptome sequencing analysis of abnormal repair pathways of pancreatic tumors.^6^ Cisplatin prevents cellular DNA repair by binding to and causing crosslinking of DNA, triggering apoptosis. While a platinum in PDAC has been examined with oxaliplatin within the FOLFIRINOX combination and NALIRIFOX, cisplatin had been rarely described in combination with nab‐paclitaxel + gemcitabine.^7^, ^8^, ^9^ We previously tested the hypothesis that a combination regimen of NABPLAGEM in patients with advanced PDAC could be safe and improve treatment efficacy and patient outcomes in a phase Ib/II open‐label, multi‐institution (conducted at three US sites), prospective study in 2019. The maximum tolerated dose (MTD) of cisplatin in combination with nab‐paclitaxel + gemcitabine was established, and 25 patients were treated with NABPLAGEM. We achieved an ORR of 71% with two CRs, 12‐month OS of 65%, and median survival of 16+ months.^10^ The current phase II study was performed to further evaluate the safety, efficacy, and impact on quality of life (QOL). An additional aim was to define biomarkers of response with the hypothesis that tumors with features of homologous recombination deficiency (HRD) would be more sensitive to the addition of cisplatin.

## 2 MATERIALS AND METHODS

### 2.1 Study patients

The study protocol (Data S1) was approved by the institutional review boards/independent ethics committee at each of the four participating institutions. All patients provided written informed consent prior to study participation. Eligible patients were 18 years or older, had a histologically or cytologically confirmed metastatic PDAC that was measurable according to the RECIST 1.1, Karnofsky performance status (KPS) ≥70%, life expectancy of ≥12 weeks, and adequate hematologic, hepatic, and renal function (see Supplement 1 for additional eligibility criteria—Data S2). Archival biopsy tissue specimens were provided for all patients that had this available at enrollment. Fresh tissue collection was optional, required additional consent, and would only be collected if the patient would be undergoing a biopsy as part of routine care. Archival and fresh biopsy tissue with adequate tumor content were analyzed via comparative genomic hybridization (CGH) and next generation sequencing (NGS) (Data S2).

### 2.2 Study design and treatment

This phase II study evaluated the efficacy and safety of nab‐paclitaxel 125 mg/m^2^, gemcitabine 1000 mg/m^2^, and cisplatin 25 mg/m^2^ (NABPLAGEM), all administered intravenously (IV) on Days 1 and 8 every 21 days (see Data S1 for study design details).

Patients received IV premedication of dexamethasone 12 mg, palonosetron 0.25 mg, and fosaprepitant 150 mg, followed by oral dexamethasone 4 mg and ondansetron 8 mg twice daily for 2 days after chemotherapy. The drug sequence was a liter of IV hydration, nab‐paclitaxel, cisplatin, gemcitabine, and another liter of IV hydration. Patients received an additional liter of IV hydration on Day 2 and 9 for Cycles 1–3 unless the cisplatin was held. Pegfilgrastim 6 mg subcutaneous (SQ) was administered on Day 9 of each cycle, or Neulasta OnPro® on Day 8. After Cycle 1, filgrastim could be administered prior to Day 8 at the Investigator's discretion to prevent neutropenia.

### 2.3 Assessments

Adverse events (AEs) were graded according to the National Cancer Institute Common Terminology Criteria for Adverse Events (NCI‐CTC) version 5.0. Hematology and serum chemistries were performed weekly. AEs grade ≥3, all grade neuropathy AEs, and any AE that lead to study treatment discontinuation were collected.

Tumor response was assessed every 9 weeks per RECIST 1.1. Serial measurements of carbohydrate antigen 19‐9 (CA 19‐9), or carbohydrate antigen‐125 (CA‐125) or carcinoembryonic antigen (CEA) in patients who did not secrete CA 19‐9 (<35 U/mL), were performed at baseline and at the beginning of every treatment cycle. Patients were followed for survival until death or study closure.

### 2.4 Patient reported outcomes

An exploratory objective was to determine if there was an improvement in QOL and pain levels (as assessed by MD Anderson Symptom Inventory [MDASI‐GI] and the Brief Pain Inventory [BPI]). The questionnaires were completed at baseline, weekly during the treatment period, and at the end of treatment. A sensitivity analysis restricted to patients with non‐zero symptoms at baseline, since those who started with scores of zero points had no room for improvement.

### 2.5 Study endpoints

The primary endpoint was 12‐month OS rate. Secondary endpoints included: complete response (CR) rate (per RECIST 1.1), Disease Control Rate (DCR) at 9 weeks, defined as CR + partial response (PR) + stable disease (SD), treatment related adverse events (TRAE), change in tumor markers (CA 19‐9, or CA‐125 or CEA in non‐expressers), and rate of normalization of tumor markers. Additional secondary endpoints included patient reported QOL and pain levels.

### 2.6 Statistical analysis

The full statistical analyses are described in Data S1. The sample size of 42 patients was based on the phase III MPACT trial, demonstrating a 12‐month OS of 35%, versus 67.5% in the previously reported phase Ib/II NABPLAGEM PCRT 12‐001 trial.^10^, ^11^ The rate of 55% historical control was selected to provide a conservative estimate of survival for the current study. To detect a difference between 35% and 55% 12‐month OS rates with 80% power, hazard ratio of 0.57, and a one‐sided 0.025 alpha‐level, 42 patients were needed.

Survival and safety analysis included any patient who received at least one dose of study treatment. Cox proportional hazards regression was used to compare survival rates between patients with high or low baseline neutrophil lymphocyte ratio (NLR). The evaluable population for tumor response was defined as patients who had at least one post‐baseline tumor assessment (for RECIST1.1), or had clinical disease progression documented in the case a post‐baseline tumor assessment was not obtained. An exploratory analysis was performed to investigate the influence of HRD pathway mutations on survival. To increase the power of this analysis, patients in this trial were combined with the prior PCRT 12‐001 trial that used identical eligibility criteria.^10^

Final data analyses were performed using Stata 17.0 (StataCorp, College Station, TX). Data from patients who were alive as of the data‐cutoff of January 12, 2023 were censored for OS.

## 3 RESULTS

### 3.1 Patient characteristics

Forty‐two patients from four US sites consented and were enrolled from September 2019 through May 2021. Median age was 66.8 years (range: 40.7–82.9), with 28 (66.7%) males and a majority were white (76.2%). Patient demographic and disease characteristics are summarized in Table 1 and Data S2. The study treatment concluded in March 2022, and the last survival contact was completed in January 2023.

**TABLE 1:** Baseline patient characteristics, *n* = 42.

Characteristic	*n* (%)
Age, years	
Median (range)	66.8 (40.7–82.9)
>65	25 (59.5)
Gender	
Male	28 (66.7)
Female	14 (33.3)
Race/ethnicity	
Non‐Hispanic white	32 (76.2)
Hispanic white	5 (11.9)
Black	3 (7.1)
Asian	2 (4.8)
KPS score	
100%	5 (11.9)
90%	18 (42.9)
80%	17 (40.5)
70%	2 (4.8)
Tumor markers at C1/D1	
CA 19‐9 ≥ 35	35 (83.3)
CA 19‐9 < 35	6 (14.3)
CA 19‐9 not collected	1 (2.4)
CA 125 ≥ 35*	5 (11.9)
CEA >3*	3 (7.1)
Primary tumor location on pancreas	
Body	15 (35.7)
Head	16 (38.1)
Head and Body	1 (2.4)
Tail	10 (23.8)
Prior cancer treatment	
Adjuvant chemotherapy	4 (9.5)
PDAC surgery—Whipple	3 (7.1)
Radiotherapy	0 (0.0)
Neutrophil‐to‐lymphocyte ratio (NLR)	
≤5	22 (52.4)
>5	20 (47.6)

* Two patients were expressors of both CA 125 and CEA.

**FIGURE 1:**
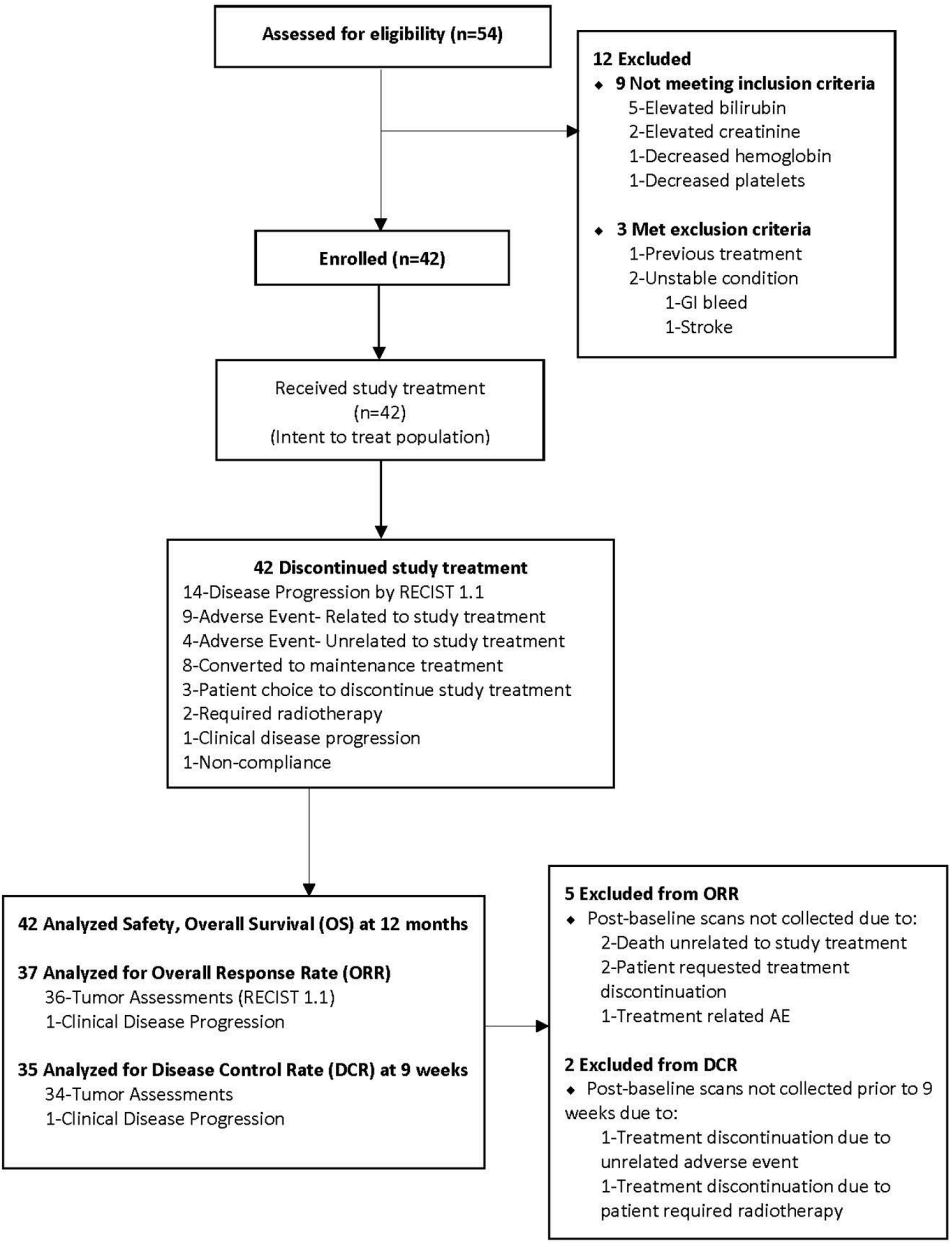
Consort diagram.

All patients received at least one dose of study treatment and were included in the safety analyses and survival analysis (*N* = 42). A total of 37 (88%) patients were evaluable for overall response rate (ORR), 36 (86%) with post‐baseline scans available for tumor assessment by RECIST 1.1, plus one additional patient with documented clinical disease progression prior to the follow‐up scan. Thirty‐five patients (83%) were evaluable for DCR at 9 weeks (Figure 1).

### 3.2 Treatment exposure

The median (range) duration of treatment was 6 (1–23) cycles, with 57% completing treatment for 6 or more cycles. Overall, 24/42 (57%) received all prescribed treatment doses (without interruptions, modifications, or discontinuation) for at least three cycles, 19/42 (45%) for at least six cycles, and 15/42 (36%) for at least nine cycles. Median (range) cumulative doses were nab‐paclitaxel 2221.5 mg, (271.2–6970.0), cisplatin 504.0 mg (54.2–1677.0), and gemcitabine 17620.0 mg (2170.0–53370.0).

### 3.3 Safety and adverse events

The most common TRAEs ≥grade 3 were: thrombocytopenia (27 patients [64.3%]) anemia (20 patients [47.6%]), neutropenia (11 patients [26.1%]), leukopenia (7 patients [16.7%]), diarrhea (5 patients [11.9%]), hypokalemia (4 patients [9.5%]), and chemotherapy induced peripheral neuropathy (CIPN) (3 patients [7.1%]) (Table 2 and Data S2). The proportion of patients who had any‐grade neuropathy was 19/42 (45%), with median (range) time to onset of 72 (9–257) days.

**TABLE 2:** Treatment‐related adverse events occurring in more than 5% of patients^a^.

Preferred term	No. (%) of patients^b^		
	Grade 3	Grade 4	Grade 5
Anemia	20 (47.6)	0	0
Colitis/Enterocolitis	2 (4.8)/1 (2.4)	0	0
Diarrhea	5 (11.9)	0	0
Fatigue	3 (7.1)	0	0
Hypokalemia	4 (9.5)	0	0
Neutrophil count decreased	7 (16.7)	4 (9.5)	0
Peripheral sensory neuropathy	3 (7.1)	0	0
Platelet count decreased	11 (26.2)	16 (38.1)	0
White blood cell decreased	6 (14.3)	1 (2.4)	0

^a^ Highest grade per person, per preferred term.

^b^ Percentage is based on the total number of patients in the safety analysis population *N* = 42.

Nine patients (21.4%) discontinued study treatment due to TRAEs: thrombocytopenia (*n* = 4), CIPN (*n* = 3), neutropenia (*n* = 1), and anemia (*n* = 1). A total of 28 Serious Adverse Events (SAEs) were reported in 22 patients (52%), with 8 (28.5%) related to study treatment (Data S2). Fatal events were assessed as unrelated to study treatment and included cardiac arrest in one patient on Cycle 1/Day 9, and a second death due to COVID‐19 viremia on Cycle 3/Day 8 (refer to Data S2 for SAE narratives).

### 3.4 Survival

**FIGURE 2:**
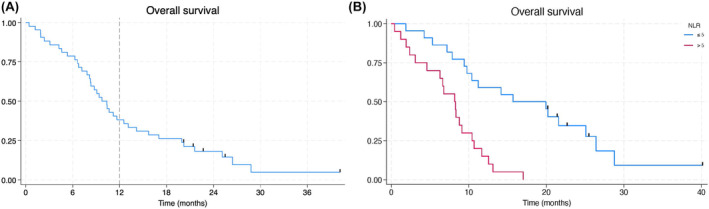
(A)Kaplan–Meier curve for overall survival. (B) Kaplan–Meier curve for overall survival—baseline NLR.

The primary objective of achieving a 12‐month survival comparable to the previous phase Ib/II NABPLAGEM PCRT 12‐001 was not achieved in this trial. The proportion (95% CI) of patients still alive at 12 months was 16/42, 38.1% (23.6–54.4%). The proportion (95% CI) of patients still alive at 18 months was 11/42, 26.2% (13.9–42.0%). Five patients remained alive at study conclusion (20–40+ months OS; Figure 2A). Median (95% CI) OS was 9.8 (8.2–12.6) months with a range 0.4–40.1+ months.

In a Cox proportional hazards regression model, patients with baseline NLR >5 had significantly worse survival compared to those with NLR ≤5 (hazard ratio = 4.3; *p* < 0.001). The 12‐month OS rate was 59% in the NLR ≤5, and 20% in the NLR >5 (Figure 2B).

### 3.5 Efficacy

Of the 37 patients evaluable for tumor response, the ORR was 40.5%, with CR in zero patients, confirmed PR in 15 (40.5%), SD in 16 (43.2%), and progressive disease (PD) in 6 (16.2%).

**FIGURE 3:**
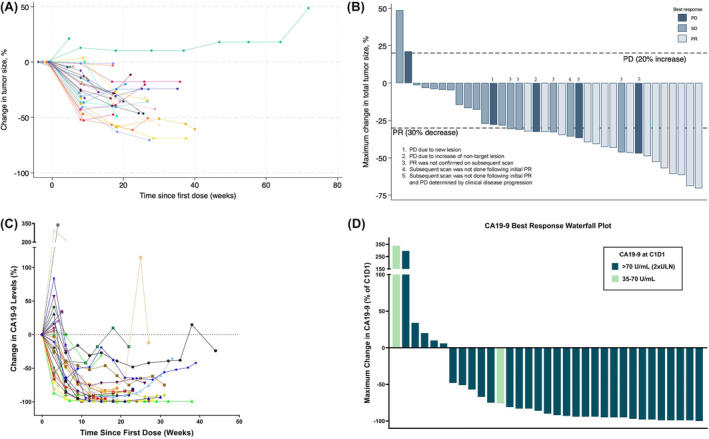
(A) Change in tumor size (sum) since baseline, by patient. (B) Response to treatment maximum change in tumor size. (C) Change in CA 19‐9 levels from baseline by patient over time. (D) Maximum % change in CA 19‐9 levels during the study.

A total of 35 patients were evaluable for DCR at 9 weeks; of these, zero achieved CR, 12 (34.3%; 95% CI, 19.1–52.2%) achieved PR, and 19 (54.3%; 95% CI, 36.6–71.2%) achieved SD, yielding a total of 31 (88.6.%; 95% CI, 73.3–96.8%) for DCR. For all patients who achieved PR at any time point throughout the study (*n* = 15), the median (range) time from enrollment to PR was 9.3 (8.1–26.9) weeks. The percent change in tumor size (sum) from baseline over time, and the maximal percent change in tumor size, are shown in Figure 3A,B, respectively.

### 3.6 Tumor markers

Of the 42 patients, 35 (83.3%) had elevated CA 19‐9 (≥35 U/mL), 5 (11.9%) had elevated CA 125 (≥35 U/mL), and 3 (7.1%) elevated CEA (≥3 U/mL) at baseline. Two patients had both elevated CA 125 and CEA. Mean ± SD baseline value of CA 19‐9 was 23,555.6 ± 45,009.0 U/mL, and CA 125 was 204.1 ± 228.8 U/mL.

In a paired analysis evaluating change from C1/D1 to end‐of‐study, CA 19‐9 significantly decreased: median (interquartile range [IQR]) change, −1047.2 (−9674.6, −44.0) U/mL; *p*, 0.001. Most patients (85%) experienced a decrease in CA 19‐9 during treatment (Figure 3C,D). CA 125 (log10) also significantly decreased in a linear mixed‐effects model (*p* < 0.001), but not in the paired analysis (*p*, 0.438). CEA was not analyzed due to insufficient sample size.

Among 31 patients with baseline CA 19‐9 levels twice the upper limit of normal (>70 U/mL), 7 achieved normalization (22.6%, 95% CI 9.6–41.1%). Of the four patients with elevated baseline CA 125, three (75.0%, 95% CI 19.4–99.4%) had CA 125 level normalized.

### 3.7 Patient reported outcomes

#### 3.7.1 MDASI‐GI

In a paired analysis evaluating change from baseline to C4/D1 (three cycles completed) timepoint to be consistent with first RECIST 1.1 assessment, there were statistically and clinically significant improvements in core symptom severity (*p* = 0.006) and symptom distress (interference) (*p* = 0.048), but not GI symptom severity (*p* = 0.437) However, in a sensitivity analysis limited to patients with GI symptoms at baseline (*n* = 40), GI symptoms significantly decreased over time (linear mixed‐effects model *p* = 0.005) (Data S2).

#### 3.7.2 BPI

In a paired analysis evaluating change from baseline to C4/D1 (Data S2), there were statistically and clinically significant improvements in pain severity (*p* = 0.013) and pain interference (*p* = 0.032) during the first three cycles of treatment. Similar results were observed in a sensitivity analysis restricting to patients with pain symptoms at baseline.

#### 3.7.3 Tumor profiling

Of 42 patients, 36 (85.7%) had archival biopsy tissue available and submitted for analysis. 31/42 (73.8%) consented to an optional tissue biopsy, and 7 (22.6%) had a biopsy performed and provided fresh tissue. Only one fresh tissue sample (01‐21) contained adequate tumor for the planned analysis, and results are presented in Data S2. Submitted tissues that failed analysis either lacked tissue, had only normal tissue, or contained denatured tumor tissue. A majority (54.7%) of these patients had commercial tumor profiling performed as standard of care, one possible reason the tumor tissue was exhausted. The somatic and germline results are shown in Data S2. A total of 3 out of 38 (7.9%) patients in this study with available genomic testing results had pathogenic germline or somatic variants in BRCA1/2 or PALB2. In the prior PCRT 12‐001 trial, 3 out of 14 (21.4%) patients with available genomic testing results had these HRD variants detected. A combined OS analysis of these 52 patients in both trials, stratified by HRD mutation status is illustrated in Data S2.

## 4 DISCUSSION

This phase II study was performed as a follow‐up to the initial study^10^ with the intent to further evaluate the safety and efficacy of NABPLAGEM in this patient population and evaluate the impact of NABPLAGEM on QOL and pain levels. However, compared to the original trial, this study reported lower 12‐month survival (38% vs. 64%), median OS (9.8 vs. 16.4 months), and ORR (42% vs. 71%).

Similar to the original study, patients in this study experienced a prompt and significant decrease in the tumor markers (Figure 3) and tumor volume (Figure 3A). The DCR was similar between the two studies, further illustrating an early response in the first three cycles of treatment. The TRAEs were similar with hematologic toxicities being most common. Based upon the safety profile of the original study, growth factor in the form of pegfilgrastim was utilized for every single patient. However, the number of patients that discontinued study treatment due to toxicity was higher in this current study (21.4%) compared to (8%) in the previous study. Additionally, 8/42 patients in this study ended NABPLAGEM with ongoing disease control and transitioned to maintenance therapy with capecitabine, of which 6 survived 12+ months. Importantly, we observed that QOL and pain improved within the first 9 weeks, countering many clinicians' and patients' fear that chemotherapy will increase the patients' symptom burden and decrease overall QOL. CIPN occurred in 45% of patients, average time to onset was 72 days, consistent with the nab paclitaxel/gemcitabine arm of MPACT trial (71 days).^12^ In both studies, we observed 2+‐year survivors; in the current study, 5 patients remained alive (20–40+ months OS) at study conclusion.

The central hypothesis for the addition of a platinum agent to the standard gemcitabine/nab‐paclitaxel backbone is the benefit seen in patients harboring HRD mutations. It is estimated that 5%–7% of patients with PDAC harbor pathogenic germline HRD variants, and 15%–17% harbor similar somatic variants.^13^ Combining patients with known core HRD mutations in both studies (Data S2), we note a trend towards longer survival. Unfortunately, due to insufficient tumor tissue for analysis and lack of commercial genomic testing results, we cannot draw conclusions regarding HRD mutations and disease response or survival on NABPLAGEM.

Apart from the limited knowledge of the patient's tumor sensitivity to cisplatin, other potential variables may have contributed to the observed lower survival and response rates in this trial compared to the original trial. Potential variables may include patient characteristics, including diversity and performance status, and the more evenly distribution of patients among the multiple sites (Data S2). Although the time on treatment was shorter in this study, there was no significant difference in dose intensity between the two studies (Data S2). Furthermore, our study findings indicate a consistent correlation between a baseline NLR >5 and worse OS, which aligns with recent findings from a post hoc analysis of the MPACT trial.^14^

When selecting candidates for NABPLAGEM, the patient's performance status and ability to comply with the treatment regimen, including long clinic days and supportive medication schedule, should be considered. Administering this regimen requires careful attention and diligent supportive care to minimize toxicities and ensure uninterrupted anti‐cancer treatment.

### 4.1 Limitations

This small phase II non‐randomized trial was conducted during the COVID‐19 pandemic, and study patients did incur interruptions and delays in treatment and increased risks while on chemotherapy. One patient died from multiorgan failure due to COVID‐19 viremia within the first 9 weeks of treatment. One of our sites was further impacted by a hurricane and although was able to recover quickly and open the clinic back up, they did have interruptions in study treatment.

The secondary endpoint of performing genomic characterization to expand the understanding of the molecular mechanisms of treatment response could not be achieved due to inadequate tumor tissue. This experience highlights the challenge of tumor testing and reinforces the FDA's proposal to limit or avoid collection of tumor biopsies in clinical trials, emphasizing the need for more effective methods of tumor analysis.^15^ Advances in circulating tumor DNA (ctDNA) have the potential to offer greater insight into PDAC tumor biology, prognosis, treatment selection, and longitudinal response monitoring, although additional prospective trials are needed.^16^

## 5 CONCLUSION

NABPLAGEM is an effective regimen in treating advanced PDAC, with early disease control noted by improvement in patient symptoms, decrease in tumor volume, and decline in tumor markers within the first 9 weeks of therapy. Given the prompt disease response observed in the original and current trial, this regimen is being studied in earlier stage PDAC patients in the neoadjuvant setting (NCT04669197, NCT03138720^17^).

## AUTHOR CONTRIBUTIONS

**Gayle S. Jameson:** Conceptualization (equal); data curation (lead); investigation (lead); writing – original draft (lead); writing – review and editing (lead). **Peter J. Hosein:** Data curation (equal); investigation (equal); writing – review and editing (equal). **Erin Pierce:** Data curation (equal); investigation (equal); writing – review and editing (equal). **Mandana Kamgar:** Data curation (equal); investigation (equal); writing – review and editing (equal). **Michael S. Gordon:** Supervision (equal); writing – review and editing (equal). **Courtney Snyder:** Data curation (equal); investigation (equal); writing – review and editing (equal). **Denise J. Roe:** Conceptualization (equal); formal analysis (equal); validation (equal); writing – review and editing (equal). **Betsy C. Wertheim:** Formal analysis (equal); visualization (equal); writing – review and editing (equal). **Marina Davey:** Data curation (equal); investigation (equal); writing – review and editing (equal). **Michael T. Barrett:** Formal analysis (equal); investigation (equal); writing – review and editing (equal). **Daniel D. Von Hoff:** Conceptualization (equal); formal analysis (equal); methodology (equal); writing – review and editing (equal). **Erkut Borazanci:** Data curation (equal); investigation (equal); writing – review and editing (equal).

## CONFLICT OF INTEREST STATEMENT

Gayle Jameson reports support for the present manuscript from Bistol Myers Squibb. Michael Barrett reports a grant/contract from National Cancer Institute. Erkut Borazanci reports consulting fees from BPG, Conjupro, Elevation Oncology, Ipsen, Nanology, Qurient, and Vivacitus. Michael Gordon reports grant/contract funding from Agenus, Arcus, Array, Corcept, Daiichi, Dynamicure, Endocyte, Forma, Genentech, GSK, I‐MAB Pharma, Imaginab, Nikang, Oncoresponse, Pfizer, Plexxicon, Pyxis, Revolution Medicine, Riboscience, Roche, Serono, SQZ, Theseu, Toray, and Tracon; and reports consulting fees from Astellas, Daiichi, Deciphera, ImaginAB, Imagining Endpoint, Morphic TX, OnQuality, Pfizer, Qualigen, Salarius, Springworks Tx, and Viracta; and reports prior patent for clinical trial enrollment; and reports participation on Data Safety Monitoring Board or Advisory Board at Tracon Pharma; and reports leadership/fiduciary role at SITC Workshop; and reports stock/stock options at Spinx Health Solution. Mandana Kamgar has received a grant from Cornerstone Pharmaceuticals. Erin Pierce reports consulting fees from Janssen and Exelixis; and reports payment/honoraria from Astellas, Exelixis, JADPRO, Janssen, Merck, Pfizer, and Sanofi; and reports payment for testimony from Exelixis; and reports support for attending meetings/travel from Exelixis, Janssen, Pfizer, and Sanofi; and reports participation on a Data Safety Monitoring Board/Advisory Board for Eisai, Exelixis, Janssen, Sanofi, and Seagen; and reports leadership or fiduciary role for Cancer Advocacy Group of Louisiana. Daniel Von Hoff reports being employed by McKesson; and reports consulting or advisory role at Imaging Endpoints, Immodulon Therapeutics, Senhwa Biosciences, Alpha Cancer Technologies, CanBas, Lixte Biotechnology, RenovoRx, TD2, Aptose Biosciences, EMD Serono, Fujifilm, Phosplatin Therapeutics, SOTIO, Geistlich Pharma, HUYA Bioscience International, Immunophotonics, Genzada Pharmaceuticals, LEAF Pharmaceuticals, Oncology Venture, Verily, Athenex, Novita Pharmaceuticals, Vicus Therapeutics, Codiak Biosciences, Agenus, Samumed, BioXCel Therapeutics, Bryologyx, Sirnaomics, AiMed, Corcept Therapeutics, Erimos Pharmaceuticals, Pfizer, GiraFPharma, Axis Therapeutics, ImmuneOncia, Viracta Therapeutics, AlaMab Therapeutics, Avesta76 Therapeutics, NeoTx, Xerient, Decoy Biosystems, Noxxon Pharma, Reglagene, Lycia Therapeutics, NGM Biopharmaceuticals, EXACT Therapeutics, Nirogy Therapeutics, Seagen, Agastiya Biotech, Amunix, Cytocom, GlaxoSmithKline, ImaginAb, SignaBlok, SonaCare Medical, Caribou Biosciences, Xenter, Compass Therapeutics, Vivacitas Oncology, OnQuality Pharmaceuticals, Sellas Life Sciences, Catamaran Bio, Thirona Biosciences, Bristol Myers Squibb, Remix Therapeutics, SMP Oncology fka SDP/Tolero, Bessor Pharma, Coordination Pharmaceuticals, Orphagen Pharmaceuticals, Red Arrow Therapeutics, Soley Therapeutics; and reports stock/ownership at Medtronic, CerRx, SynDevRx, United Healthcare, Anthem Inc, Stromatic Pharma, Systems Oncology, Stingray Therapeutics, FORMA Therapeutics, Orpheus Biosciences, AADi, and Origin Commercial Advisors; and reports patents/royalties/intellectual property at Intramedullary Catheter, Methods of Human Prostate Cancer, Use of 5 6‐Dihydro‐5‐Azacytidine in treatment of Prostate Cancer, Targeting Site‐2 Protease (S2P) for the Treatment of Pancreatic Cancer (pending), Targeting Ecto‐5‐Nucleotidase (Cd73) for the Treatment of Pancreatic Cancer, Targeting a Protein Tyrosine Phosphotase‐PRL‐1 for the treatment of pancreatic cancer (pending), targeting a protein PRC1 for the treatment of pancreatic cancer (pending), Targeting Ecto‐5‐Nucleotidase (CD73) for the treatment of pancreatic cancer (pending), Protein Kinase Inhibitors (pending), methods, compounds and compositions with genotype selective anticancer activity (pending), methods and kits to predict therapeutic outcome of BTK inhibitors (pending), muscle fatigue substance cytokines and methods of inhibiting tumor growth therewith (pending), and 2‐aryl‐pyridylazoles for the treatment of solid tumors such as pancreatic cancer (pending); and reports research funding from Lilly, Genentech, Celgene, Incyte, Merrimack, Plexxikon, Minneamrita Therapeutics, Abbvie, Aduro Biotech, Cleave Biosciences, CytRx Corporation, Daiichi Sankyo, Deciphera, Endocyte, Exelixis, Five Prime Therapeutics, Gilead Sciences, Merck, Pfizer, Pharmacyclics, Phoenix Biotech, Samumed, Strategia, and Halozyme. The other authors declare no conflicts of interest.

## ETHICS STATEMENT

The ethical approval for this study was obtained from Western Institutional Review Board (site: HonorHealth Research Institute, PI: Jameson), University of Miami Institutional Review Board (site: University of Miami, PI: Hosein), Medical College of Wisconsin/Froedtert Hospital Institutional Review Board (site: Medical College of Wisconsin, PI: Kamgar), and Ochsner Clinic Foundation Institutional Review Board (site: Ochsner Health, PI: Pierce).

## TRIAL REGISTRATION

ClinicalTrials.gov Identifier: NCT03915444.

## Supporting information


Data S1.

Data S2.

Data S3.

## Data Availability

The data that support the findings of this study are available on request from the corresponding author. Data availability is subject to institutional approval.

## References

[1] Siegel RL , Miller KD , Wagle NS , Jemal A . Cancer statistics, 2023. CA Cancer J Clin. 2023;73(1):17‐48. doi:10.3322/caac.21763 36633525

[2] Singhi AD , Koay EJ , Chari ST , Maitra A . Early detection of pancreatic cancer: opportunities and challenges. Gastroenterology. 2019;156(7):2024‐2040. doi:10.1053/j.gastro.2019.01.259 30721664 PMC6486851

[3] SEER*Stat Databases: November 2021 Submission . NIH National Cancer Institute Surveillance, Epidemiology, and End Results Program. Accessed June 14, 2023. https://seer.cancer.gov/data‐software/documentation/seerstat/nov2021/

[4] Barrett MT , Deiotte R , Lenkiewicz E , et al. Clinical study of genomic drivers in pancreatic ductal adenocarcinoma. Br J Cancer. 2017;117(4):572‐582. doi:10.1038/bjc.2017.209 28720843 PMC5558689

[5] Waddell N , Pajic M , Patch AM , et al. Whole genomes redefine the mutational landscape of pancreatic cancer. Nature. 2015;518(7540):495‐501. doi:10.1038/nature14169 25719666 PMC4523082

[6] Liang WS , Craig DW , Carpten J , et al. Genome‐wide characterization of pancreatic adenocarcinoma patients using next generation sequencing. PLoS One. 2012;7(10):e43192. doi:10.1371/journal.pone.0043192 23071490 PMC3468610

[7] Conroy T , Desseigne F , Ychou M , et al. FOLFIRINOX versus gemcitabine for metastatic pancreatic cancer. N Engl J Med. 2011;364(19):1817‐1825. doi:10.1056/NEJMoa1011923 21561347

[8] Wainberg ZA , Bekaii‐Saab T , Boland PM , et al. First‐line liposomal irinotecan with oxaliplatin, 5‐fluorouracil and leucovorin (NALIRIFOX) in pancreatic ductal adenocarcinoma: a phase I/II study. Eur J Cancer. 2021;151:14‐24. doi:10.1016/j.ejca.2021.03.028 33957442

[9] Wainberg ZA , Melisi D , Macarulla T , et al. NAPOLI‐3: a randomized, open‐label phase 3 study of liposomal irinotecan + 5‐fluorouracil/leucovorin + oxaliplatin (NALIRIFOX) versus nab‐paclitaxel + gemcitabine in treatment‐naïve patients with metastatic pancreatic ductal adenocarcinoma (mPDAC). J Clin Oncol. 2023;41(4_suppl):LBA661. doi:10.1200/JCO.2023.41.4_suppl.LBA661 PMC11664154 37708904

[10] Jameson GS , Borazanci E , Babiker HM , et al. Response rate following albumin‐bound paclitaxel plus gemcitabine plus cisplatin treatment among patients with advanced pancreatic cancer: a phase 1b/2 pilot clinical trial. JAMA Oncol. 2019;6(1):125‐132. doi:10.1001/jamaoncol.2019.3394 PMC6777241 31580386

[11] von Hoff DD , Ervin T , Arena FP , et al. Increased survival in pancreatic cancer with nab‐paclitaxel plus gemcitabine. N Engl J Med. 2013;369(18):1691‐1703. doi:10.1056/NEJMoa1304369 24131140 PMC4631139

[12] Personal correspondence between Gayle Jameson with Daniel D . Von Hoff on MPACT trial. 2014.

[13] Pishvaian MJ , Bender RJ , Halverson D , et al. Molecular profiling of patients with pancreatic cancer: initial results from the know your tumor initiative. Clin Cancer Res. 2018;24(20):5018‐5027. doi:10.1158/1078-0432.CCR-18-0531 29954777

[14] Goldstein D , El‐Maraghi RH , Hammel P , et al. Nab‐paclitaxel plus gemcitabine for metastatic pancreatic cancer: long‐term survival from a phase III trial. J Natl Cancer Inst. 2015;107(2):dju413. doi:10.1093/jnci/dju413 25638248

[15] Araujo D , Greystoke A , Bates S , et al. Oncology phase I trial design and conduct: time for a change—MDICT guidelines 2022. Ann Oncol. 2023;34(1):48‐60. doi:10.1016/j.annonc.2022.09.158 36182023

[16] Lapin M , Edland KH , Tjensvoll K , et al. Comprehensive ctDNA measurements improve prediction of clinical outcomes and enable dynamic tracking of disease progression in advanced pancreatic cancer. Clin Cancer Res. 2023;29(7):1267‐1278. doi:10.1158/1078-0432.CCR-22-3526 36662807 PMC10068442

[17] Borazanci EH , Jameson GS , Snyder CE , et al. Paclitaxel protein bound (A) plus gemcitabine (G) plus cisplatin (C), and paricalcitol (P)neoadjuvant therapy for localized pancreatic ductal adenocarcinoma (PDAC). J Clin Oncol. 2020;38(15_suppl):4631. doi:10.1200/jco.2020.38.15_suppl.4631

