# Supplementary material for: Phase II clinical trial of nab‐paclitaxel plus cisplatin plus gemcitabine (NABPLAGEM) in patients with untreated advanced pancreatic cancer
Source: Cancer Med. 2024 Jun 22;13(12):e7412. doi: 10.1002/cam4.7412 (PMC11192994; doi:10.1002/cam4.7412)
Supplement: Supplementary file 1 — Data S1. [file CAM4-13-e7412-s001.pdf]

Protocol #: AX-CL-PANC-PI-13301

A PHASE II TRIAL OF NAB-PACLITAXEL PLUS CISPLATIN PLUS GEMCITABINE IN PATIENTS WITH PREVIOUSLY UNTREATED METASTATIC PANCREATIC DUCTAL ADENOCARCINOMA

NCT03915444
IND Exempt

Sponsor
HonorHealth Research Institute

Lead Principal Investigator

Gayle S. Jameson, MSN, ACNP BC, AOCN
HonorHealth Research Institute
10510 N. 92nd Street
Scottsdale, AZ 85258
Tel: 480-323-1350
Fax: 480-323-1359
Email: gjameson@honorhealth.com

Consulting Investigator

Daniel D. Von Hoff, MD, FACP
Translational Genomics Research Institute (TGen)
445 N. Fifth Street. Phoenix, AZ 85004
Tel. 602-343-8492
dvh@tgen.org

Sub-Investigator

Erkut Borazanci, MD
HonorHealth Research Institute
10510 N. 92nd Street
Scottsdale, AZ 85258
Tel: 480-323-1350
Fax: 480-323-1359
Email: eborazanci@honorhealth.com

Sub-Investigator	Sub-Investigator	Sub-Investigator
Karen L. Ansaldo, PharmD<br>HonorHealth Research Institute<br>10510 N. 92nd St.<br>Scottsdale, AZ 85258<br>Tel. 480-323-1339<br>KAnsaldo@HonorHealth.com	Ludmil Alexandrov, PhD<br>University of California, San Diego<br>9500 Gilman Drive<br>CMM East, Room 2042<br>La Jolla, CA 92093-0695<br>l2alexandrov@ucsd.edu	Michael Barrett, PhD<br>Mayo Clinic Arizona<br>Mayo Collaborative Cancer Research Building, Cr2-111<br>13208 E. Shea Blvd, Suite 100<br>Scottsdale, AZ 85259

CONFIDENTIAL
The information contained in this document is confidential and the proprietary property of Sponsor. Any unauthorized use or disclosure of such information without the prior written authorization of the Sponsor is prohibited

Medical Monitor

Michael Gordon, MD
HonorHealth Research Institute
10510 N. 92nd Street
Scottsdale, AZ 85258
480-323-1350
Fax: 480-323-1359
Email: mgordon@honorhealth.com

Biostatistician

Kevin Gosselin, M.Ed, M.S., Ph.D.
Director, Academics and Biostatistics
HonorHealth Research Institute
10510 N. 92nd Street
Scottsdale, AZ 85258
Kgosselin@honorhealth.com
480-323-3251

Contract Research Organization (CRO)

Triligent International
27271 Las Ramblas, Suite 100
Mission Viejo, CA 92691
Email: AXCLPANC@triligent.com

SOURCE OF SUPPORT

This study is funded by

VERSION DATES

VERSION 5.1 JUN 16, 2021

PREVIOUS VERSIONS:

Version 4.0 OCT 15, 2020

Version 3.0 DEC 19, 2019

Version 2.0 MAY 28, 2019

Version 1.1 April 17, 2019

Version 1.0 April 01, 2019

INVESTIGATOR'S PROTOCOL AGREEMENT

Protocol No.: AX-CL-PANC-PI-13301

Study Title: A Phase II Trial of Nab-Paclitaxel plus Cisplatin plus Gemcitabine in Patients with Previously Untreated Metastatic Pancreatic Ductal Adenocarcinoma

Version Date: Jun 16, 2021

I confirm that my staff and I have carefully read and understand this protocol. I/we agree to comply with the procedures and terms of the study specified herein. In particular, I/we have agreed to:

- Abide by all obligations stated on Form FDA 1572 and on other document(s) required by local regulatory authority.
- Retain records and documents related to this trial for at least 7 years after the last approval of a marketing application in an International Conference on Harmonization (ICH) region and until there are no pending or contemplated marketing applications in an ICH region or at least 7 years have elapsed since the formal discontinuation of clinical development of the investigational products.
- Comply with Good Clinical Practice (GCP) and all applicable regulatory requirements.
- Maintain confidentiality and assure security of HonorHealth Research Institute (HRI), Celgene, and Triligent confidential documents.
- Obtain Institutional Review Board (IRB) approval of the protocol, any amendments to the protocol, and periodic re-approval as required, and to keep the IRB informed of adverse events and periodically report the status of the study to them.
- Not implement any deviations from or changes to the protocol without agreement from the sponsor and prior review and written approval from the IRB, except where necessary to eliminate an immediate hazard to the subjects or for administrative aspects of the study (where permitted by all applicable regulatory requirements).
- Assure that each patient enrolled into the trial has read, understands, and has signed the Informed Consent.
- Ensure that I and all persons assisting me with the study are adequately informed and trained about the investigational drug and of their study-related duties and functions as described in the protocol.
- Make prompt reports of serious adverse events (SAEs) and deaths (within 1 business day of becoming aware of the event) to the Sponsor (via Triligent) and Celgene as indicated in Section 7 of the protocol.
- Assure access to study monitors.
- Prepare and maintain adequate and accurate case histories designed to record all observations and other data pertinent to the investigation on each individual treated in the investigation.
- Arrange for the transfer of appropriate data from case histories to case report forms for the collection and transmission of data to the Sponsor.
- Cooperate fully with any study-related GCP audit as performed by quality assurance group specified by the sponsor.
- Abide by the stipulations in the Disclosure of Data section and the manuscript preparation/authorship guidelines established at the outset of the study.

Investigator's Printed Name: ______________________________

Investigator's Signature: ______________________ Date: ______________

SAE REPORTING

All SAEs must be reported via Medwatch Form 3500A along with the SAE Report Form Cover letter promptly to both Triligent and Celgene after the Investigator recognizes/classifies the event as a SAE.

For life-threatening or fatal events, the Investigator must report initial information on the SAE **within 1 business day** of becoming aware of the event, preferably by email or alternatively by fax or phone (Refer to protocol section 7.9 Investigator Reporting Serious Adverse Events).

Note: Participating study sites should NOT report SAEs to the FDA. Celgene will be responsible for reporting to FDA.

SAE Reporting to the Sponsor (via CRO, Triligent):

Phone:	317-402-9777
Fax:	949-203-6151
Email:	SAE@Triligent.com

AND

Celgene Corporation

Global Drug Safety and Risk Management
86 Morris Avenue
Summit, New Jersey 07901

**Phone:**	908-673-9667
**Toll Free:**	800-640-7854
**Fax:**	908-673-9115
**Email:**	drugsafety@celgene.com

For any additional questions regarding reporting requirements of a SAE, please contact Triligent as listed in the study contact list.

List of Abbreviations	
AE	Adverse Event
ALT (SGPT)	Alanine Aminotransferase (SGPT)
ALP	Alkaline Phosphatase
ANC	Absolute Neutrophil Count
ASCO	American Society of Clinical Oncology
AST (SGOT)	Aspartate Aminotransferase (SGOT)
β-hCG	Beta subunit of human chorionic gonadotrophin (hCG)
BPI	Brief Pain Inventory
BSA	Body Surface Area
BUN	Blood Urea Nitrogen
CA-125	Cancer antigen 125
CA19-9	Carbohydrate antigen 19-9
CBC	Complete Blood Count
CEA	Carcinoembryonic Antigen
CFR	Code of Federal Regulations
C_{max}	Maximum observed plasma concentration
CMH	Cochran-Mantel-Hanzel
CNV	Copy Number Variants
CNS	Central Nervous System
CRF	Case Report Form
CR	Complete Response
CRO	Contract Research Organization
CT	Computed Tomography
CTCAE	Common Terminology Criteria for Adverse Events
DHHS	Department of Health and Human Services
ECG	Electrocardiogram
eCRF(s)	Electronic Case Report Form(s)
FDA	Food and Drug Administration
^{18}F-FDG	Fluorine-18 Fluoro-D-Glucose
FFPE	Formalin-Fixed Paraffin-Embedded
GCP	Good Clinical Practice
G-CSF	Granulocyte Colony Stimulating Factors
HIPPA	Health Insurance Portability and Accounting Act
HIV	Human Immunodeficiency Virus
HRI	HonorHealth Research Institute
HUS	Hemolytic Uremia Syndrome
ICF	Informed Consent Form
ICH	International Conference of Harmonization
IEC	Independent Ethics Committee
IND	Investigational New Drug
INR	International Normalized Ratio
IP	Investigational Product
IRB	Institutional Review Board
IV	Intravenous
KPS	Karnofsky Performance Status
LD	Longest Diameter
MDASI	MD Anderson Symptom Inventory
MRI	Magnetic Resonance Imaging
MTD	Maximum Tolerated Dose

List of Abbreviations	
Nab	Nanoparticle albumin bound
NCI	National Cancer Institute
NRS	Numeric Rating Scale
NS	Normal Saline
NSCLC	Non-Small Cell Lung Cancer
OS	Overall Survival
PD	Progressive Disease
PDA	Pancreatic Ductal Adenocarcinoma
PFS	Progression-Free Survival
PO	Orally
PR	Partial Response
QC	Quality Control
RECIST	Response Evaluation Criteria in Solid Tumors
RPLS	Reversible Posterior Leukoencephalopathy Syndrome
SAE	Serious Adverse Event
SD	Stable Disease
SGOT	Serum Glutamate Oxaloacetic Transaminase
SGPT	Serum Glutamate Pyruvic Transaminase
SIADH	Syndrome of Inappropriate Antidiuretic Hormone Secretion
SPARC	Secreted Protein Acidic and Rich in Cysteine
SUSAR	Suspected Unexpected Serious Adverse Reaction
TGen	Translation Genomic Research Institute
ULN	Upper Limit of Normal
WBC	White Blood Cells

TRIAL SYNOPSIS

**Title and No:** A Phase II Trial of Nab-Paclitaxel plus Cisplatin plus Gemcitabine in Patients with Previously Untreated Metastatic Pancreatic Ductal Adenocarcinoma.<br>Protocol # AX-CL-PANC-PI-13301<br>IND Exempt April, 2019<br>Version 5.1 dated Jun 16, 2021
Trial Funded by Celgene<br><br>**Sponsor:** HonorHealth Research Institute, 10510 N. 92nd Street Scottsdale, AZ 85258
**Consulting Investigator:**<br>Daniel D. Von Hoff, MD<br>Translational Genomics Research Institute (TGen) An Affiliate of City of Hope
**Principal Investigator Site:**<br>Gayle Jameson ACNP- BC, AOCN - HonorHealth Research Institute, Scottsdale, AZ
**Medical Monitor:**<br>Michael Gordon, MD<br>Medical Director - HonorHealth Research Institute, Scottsdale, AZ
**Clinical Phase:** Phase II Open-Label study
**Objectives:**<br>**Primary:** The primary objective of this study is to evaluate the 12-month overall survival rates in patients with metastatic PDA treated with nab-paclitaxel plus cisplatin plus gemcitabine.<br><br>**Secondary:**<br>1. Evaluate the safety of cisplatin plus nab-paclitaxel plus gemcitabine.<br>2. Evaluate the efficacy of cisplatin plus nab-paclitaxel plus gemcitabine based on complete response rate (RECIST 1.1), disease control rate at 9 weeks, and change in CA 19-9 (or CA 125, or CEA if not expressers of CA 19-9).<br>3. Evaluate the participant’s self-reported quality of life and pain levels during this study.<br>4. To expand understanding of the molecular mechanisms of treatment response.
**Trial Design:** Open-label, single arm, study
**No. Patients:** Total of 50 patients recruited
**Name, dose of drugs:**<br><br>**Investigational Site’s commercial product inventory to be used.**<br>• Pre cisplatin hydration: 0.9% Sodium Chloride Injection 1000 mL with Magnesium Sulfate 2 grams IV infusion over 2 hours on days 1 and 8 repeated every 21 days.<br>• Palonosetron (Aloxi®) 0.25 mg IV, fosaprepitant (Emend®) 150 mg IV and dexamethasone 12 mg IV, or equivalent antiemetic regimen within 30 minutes prior to treatment on days 1 and 8, repeated every 21 days Patients will continue oral antiemetic prophylaxis at home with ondansetron 8mg bid and dexamethasone 4mg bid for 2 days after chemotherapy. The type of antiemetic prophylaxis used can vary based on institutional procedures.

- Nab-paclitaxel 125mg/m^2 over 30 minute IV infusion on days 1 and 8 repeated every 21 days. Following administration, the intravenous line should be flushed with sodium chloride 9 mg/ml (0.9%) solution for injection to ensure complete administration of the complete dose, according to local practice.

- Cisplatin 25mg/m^2 in 500 mL of NS (the investigator may reduce the volume to 250 mL of NS if clinically indicated) over 60 minute IV infusion on days 1 and 8 repeated every 21 days followed by:

- Gemcitabine 1000mg/m^2 in 500 mL of NS (the investigator may reduce the volume to 250 mL of if clinically indicated) over 30 minute IV infusion on days 1 and 8 repeated every 21 days.

- Post cisplatin hydration: IV fluids up to 1000 mL (with additives as clinically indicated) IV given as infusion on days cisplatin is administered on days 1 and 8 repeated every 21 days. May start at the same time as the gemcitabine infusion. Additional post-cisplatin hydration of 1 liter of IV fluids on days 2 & 9 required for Cycles 1-3. Volume may be reduced to 500 ml at the Investigator's discretion. Note: If the cisplatin is held in any cycle, the additional hydration on day of chemotherapy or day after would not be needed.

Patient Population: Patients with untreated metastatic pancreatic ductal adenocarcinoma.

Endpoints:

Primary: To evaluate the 12-month overall survival rates in patients with metastatic PDA.

Secondary:

- Treatment-related toxicities.

- Complete response rate as defined by CT scan using RECIST 1.1 criteria and CA 19-9 (or CA 125, or CEA if not expressers of CA 19-9) down to normal limits (from at least > 2X ULN).

- Disease control rate (CR, PR and SD at 9 weeks) in patients with metastatic PDA.

- Change in CA 19-9 (or CA 125, or CEA if not expressers of CA 19-9) in this patient population.

- Rates of normalization of CA 19-9 (or CA 125, or CEA if not expressers of CA 19-9).

- Participant's self-reported quality of life and pain levels during this study utilizing the MD Anderson Symptom Inventory (MDASI-GI) and Brief Pain Inventory (BPI).

- To expand understanding of the molecular mechanisms of treatment response by performing genomic characterization on patient's tumor biopsies. Our hypothesis is that the PDAs that respond to the nab-paclitaxel, cisplatin, gemcitabine regimen will have distinct mutational and CNV signatures.

Inclusion criteria:

1. Age ≥ 18 years of age; male or female.

2. Histologically or cytologically confirmed metastatic pancreatic ductal adenocarcinoma.

3. Capable of providing informed consent and complying with trial procedures.

4. Karnofsky Performance Status (KPS) of ≥ 70%.

5. Life expectancy ≥ 12 weeks.

6. Measurable tumor lesions according to RECIST 1.1 criteria.

7. < Grade 2 pre-existing peripheral neuropathy per NCI CTCAE, Version 5.0

8. Patient has acceptable coagulation status as indicated by an INR ≤1.5 x ULN. Patients on anticoagulation can be included at the discretion of the investigator.

9. Patients must have normal organ and marrow function as defined below:

   - Absolute neutrophil count ≥1,500/mm^3
   - Platelet concentration ≥100,000/mm^3 with no platelet transfusions within 7 days prior to laboratory sample
   - Hemoglobin > 9.0g/dL
   - Hematocrit level > 27%
   - Total bilirubin within 1.25 times institutional upper limit of normal (ULN)
   - Alanine aminotransferase (ALT) and AST ≤ 10 X institutional ULN
   - Serum creatinine < 1.5 mg/dl

10. Females of child-bearing potential (defined as a sexually mature woman who (1) has not undergone hysterectomy [the surgical removal of the uterus] or bilateral oophorectomy [the surgical removal of both ovaries] or (2) has not been naturally postmenopausal for at least 24 consecutive months [i.e., has had menses at any time during the preceding 24 consecutive months]) must:

    a. Either commit to true abstinence* from heterosexual contact (which must be reviewed on a monthly basis), or agree to use, and be able to comply with, effective contraception without interruption, 28 days prior to starting IP therapy (including dose interruptions), and while on study medication or for a longer period if required by local regulations following the last dose of IP; and

    b. Have a negative serum pregnancy test (β -hCG) result at screening and agree to ongoing pregnancy testing during the course of the study, and after the end of study therapy. This applies even if the subject practices true abstinence* from heterosexual contact.

11. Male subjects must practice true abstinence* or agree to use a condom during sexual contact with a pregnant female or a female of childbearing potential while participating in the study, during dose interruptions and for 6 months following discontinuation from study treatment, even if he has undergone a successful vasectomy.

* True abstinence is acceptable when this is in line with the preferred and usual lifestyle of the subject. [Periodic abstinence (e.g., calendar, ovulation, symptothermal, post-ovulation methods) and withdrawal are not acceptable methods of contraception].

Exclusion criteria:

1. Patients must have received no previous radiotherapy, surgery, chemotherapy or investigational therapy for the treatment of metastatic disease. Prior treatments in the neoadjuvant and/or adjuvant setting with gemcitabine and/or 5-FU based therapies or gemcitabine and/or 5FU administered as a radiation sensitizer are allowed, provided at least 6 months have elapsed since completion of the last dose and no lingering toxicities are present.

2. Palliative surgery and/or radiation treatment less than 4 weeks prior to initiation of study treatment.

3. Exposure to any investigational agent within 4 weeks prior to initiation of study treatment.

4. Evidence of central nervous system (CNS) metastasis (negative imaging study, if clinically indicated, within 4 weeks of Screening Visit).

5. History of other malignancies (except cured basal cell carcinoma, superficial bladder cancer or carcinoma *in situ* of the cervix) unless documented free of cancer for ≥5 years.

6. Current, serious, clinically significant cardiac arrhythmias as determined by the investigator.

7. History of HIV infection.

8. Active, clinically significant serious infection requiring treatment with antibiotics, anti-virals or anti-fungals.

9. Major surgery within 4 weeks prior to initiation of study treatment.

10. Any condition in the opinion of the principal investigator that might interfere with the patient's participation in the study or in the evaluation of the study results.

11. Any condition in the opinion of the principal investigator that is unstable and could jeopardize the patient's participation in the study.

Statistical Methods: The primary endpoint for this clinical trial is to evaluate the 12-month overall survival rates in patients with metastatic PDA. 42 patients are needed to detect a difference between 35% and 55% 12-month OS rates with 80% power, hazard ratio of 0.57, and a one-sided 0.025 alpha-level. Time-to-event endpoints, including PFS and OS will be assessed using the Kaplan-Meier method. Objective response rates, clinical benefit response, and CA 19-9, will be summarized descriptively.

Logistic regression will be used to assess the impact of patient characteristics on response and toxicity rates. The distribution of time-to-event endpoints (e.g. response duration, progression-free survival, overall survival) will be estimated using the Kaplan and Meier method. Cox (proportional hazards) regression will be used to evaluate multivariable predictive models of time-to-event outcomes.

Stopping Rules: The study will be stopped early based on either one of the following criteria being met:

1. Treatment related deaths > 2 patients in the first 25 patients, as that is the number we observed in the original 25 patient study.
2. Treatment related bleeding in the first 25 patients that is life threatening and/or fatal in > 1 patient.

Table of Contents

1 BACKGROUND ... 15
1.1 Potential Risks of nab-Paclitaxel ... 16
1.2 Potential risks of Cisplatin ... 16
1.3 Potential Risks of Gemcitabine ... 16
1.4 Potential risks of nab-Paclitaxel plus Gemcitabine ... 17
1.5 Potential risks of nab-Paclitaxel, plus Cisplatin, plus Gemcitabine ... 18
1.6 Study and Dose Rationale ... 18
1.7 Sequence of Drug Administration and Rationale ... 18
1.8 Quality of Life and Pain Assessments ... 18
1.9 Tumor Analysis ... 19
2 OBJECTIVES ... 19
2.1 Primary Objective ... 19
2.2 Secondary Objectives ... 19
2.3 Overview ... 20
2.4 Primary Endpoints ... 20
2.5 Secondary Endpoints ... 20
3 STUDY POPULATION ... 21
3.1 Patient Selection and Study Duration ... 21
3.2 Inclusion Criteria ... 21
3.3 Exclusion Criteria ... 22
3.4 Patient Enrollment ... 23
3.5 Patient Discontinuation ... 23
4 STUDY TREATMENT ... 24
4.1 Administration and Dosing ... 24
4.2 Body Surface Area Calculation ... 25
4.3 Dose Modification for Toxicity ... 25
4.3.1 Hematological Toxicity ... 25
4.3.2 Non-hematological Toxicity ... 27
4.3.2.1 Peripheral Neuropathy ... 27
4.3.2.2 Nephrotoxicity ... 27
4.3.2.3 Cutaneous Toxicity ... 28
4.3.2.4 Gastrointestinal Toxicity ... 28
4.3.2.5 Pulmonary Embolism ... 28
4.3.2.6 Interstitial Pneumonitis ... 28
4.3.2.7 Hypersensitivity Reactions ... 29
4.3.2.8 Prophylaxis use of Colony Stimulating Factors ... 29
4.3.2.9 Prophylaxis against Sepsis ... 29
4.4 Intended Dose Delays ... 29
4.5 Concomitant Therapy ... 30
4.6 Concomitant Therapies Requiring Caution ... 30
5 STUDY ASSESSMENTS ... 30
5.1 Laboratory Assessments ... 30
5.2 Screening (Within 21 Days Prior to First Dose on C1/D1) ... 30
5.3 On-Study Assessments ... 31
5.3.1 Tissue Analysis ... 31
5.3.2 Day 1 of each cycle (except where noted) ... 32
5.3.3 Day 8 of each cycle ... 33
5.3.4 Day 15 of each cycle ... 33
5.3.5 Tumor Assessments every three cycles (Prior to Cycles 4, 7, 10, etc.) ... 34

5.4 End of Treatment....34
5.5 Follow-Up Assessments after End of Study or Early Termination 34

6 EVALUATION OF RESPONSE.... 35
6.1 Best Overall Response....35
6.2 Overall Tumor Response....35
6.3 Not Evaluable 35
6.4 Progression-free Survival 35
6.5 Survival 35
6.7 Disease Definitions....36
6.8 Methods of Measurement....36
6.9 Baseline Documentation of "Target" and "Non-target" Lesions 37
6.10 Response Criteria....38
6.11 Evaluation of Best Overall Response....39
6.12 Duration of Overall Response....39
6.13 Duration of Stable Disease 39

7 SAFETY.... 40
7.1 Adverse Events 40
7.2 Serious Adverse Events 41
7.3 Adverse Event Severity 42
7.4 Duration....42
7.5 Action Taken 42
7.6 Outcome....42
7.7 Causality Assessment 42
7.8 Patient Reporting of AEs and SAEs....43
7.9 Investigator Reporting Adverse Events....43
7.9.1 Expedited Reporting by Investigator to (Sponsor/Celgene)....44
7.9.2 IRB Notification of SAEs 45
7.10 SAE Follow-Up 45
7.10.1 Sponsor Notification of Post-Study SAEs....45
7.11 Pregnancy 45
7.12 Overdose....46

8 STATISTICAL CONSIDERATIONS 47
8.1 Trial Design 47
8.2 Sample Size Considerations....47
8.3 Statistical Analysis Plan....47
8.3.1 Analysis of the Conduct of the Trial....47
8.3.2 Patient Disposition 47
8.3.3 Analysis of Patient Characteristics 48
8.4 Efficacy Analysis....48
8.5 Safety and Tolerance Analysis 49
8.5.1 Stopping Rules....49
8.6 Laboratory Assessments 49
8.6.1 Hematology Parameters 49
8.6.2 Clinical Chemistry 49
8.6.3 Peripheral Neuropathy 50
8.7 Replacement of Patients....50

9 ETHICAL, LEGAL, AND ADMINISTRATIVE ASPECTS 50
9.1 Ethics 50
9.1.1 Institutional Review Board/Ethics Committee Approval 50
9.1.2 Ethical Conduct of the Study....50
9.1.3 Informed Consent 50

9.2 Disclosure of Data 51
9.2.1 Confidentiality 51
9.2.2 Publication 51
9.2.3 Trial Registration and Results Posting Requirements....................51
9.3 Quality Assurance 52
9.3.1 Study Data Monitoring Plan....................52
9.3.2 Data Safety and Monitoring....................52
9.3.3 On-site Audits 52
9.5 Investigator Documentation 53
9.5.3 Investigator Protocol Agreement 53
9.5.4 Financial Disclosures 53
9.5.5 Laboratory Certification and Normal Ranges....................53
9.5.6 Records Retention 54

10 DATA MANAGEMENT 54
10.1 Case Report Forms 54
10.2 Source Documents 54

REFERENCES.................... 55

APPENDIX A: SCHEDULE OF TREATMENT AND EVALUATIONS 58

APPENDIX B: KARNOFSKY PERFORMANCE STATUS 61

APPENDIX C: REVISED NATIONAL CANCER INSTITUTE COMMON TERMINOLOGY CRITERIA FOR ADVERSE EVENTS (NCI CTCAE), VERSION 5.0 (PUBLISHED 27 NOVEMBER 2017) 62

APPENDIX D: DRUG STUDY DRUG PREPARATION, DOSING, ADMINISTRATION AND STORAGE 63

APPENDIX E: THE MD ANDERSON SYMPTOM INVENTORY GASTROINTESTINAL CANCER MODULE (MDASI-GI) (TO BE SUBMITTED SEPARATELY FROM PROTOCOL FOR IRB APPROVAL).................... 64

APPENDIX F: THE BRIEF PAIN INVENTORY (BPI) – SHORT FORM (TO BE SUBMITTED SEPARATELY FROM PROTOCOL FOR IRB APPROVAL) 65

APPENDIX G: 2006 UPDATE OF ASCO PRACTICE GUIDELINE RECOMMENDATIONS FOR THE USE OF WHITE BLOOD CELL GROWTH FACTORS: GUIDELINE SUMMARY 66

LIST OF TABLES

Table 1. Dose Reduction Schema 25
Table 2. Dose Modifications for Day 1 of Each Cycle (Hematologic Toxicity) 26
Table 3. Dose Modification for Day 8 of Each Cycle (Hematologic Toxicity)* 26
Table 4. Nab-paclitaxel and gemcitabine Dose Modifications for Day 1 of Each Cycle (Non-hematologic Toxicity)* 27
Table 5. Nab-paclitaxel and gemcitabine Dose Modifications Day 8 Each Cycle (Non-hematological Toxicity) 27
Table 6. RECIST 1.1 Target Lesion Response Criteria 38
Table 7. RECIST 1.1 Non-target Lesion Response Criteria 38
Table 8. RECIST Overall Response Criteria 39

1 BACKGROUND

Pancreatic cancer continues to be a highly lethal disease with an overall 5 year survival of only 8%. Since 2004, the incidence of pancreatic cancer has been increasing by 1.5% per year and it is estimated that there will be 55,440 new cases diagnosed in the United States in 2018, with 43,330 expected deaths. Pancreatic cancer is the fourth most common cause of cancer-related deaths in both men and women, and the incidence is slightly higher in men versus women (ACS 2018). Of all types of pancreatic cancers, pancreatic ductal adenocarcinoma (PDA) is by far the most common, representing 80% of cases (Dragovich 2012).

Due to the lack of adequate screening techniques, greater than 80% of patients at the time of diagnosis present with unresectable, advanced disease. Standard treatment options for inoperable patients with locally advanced and metastatic PDA have been quite limited. Gemcitabine monotherapy, approved by the FDA in 1996, demonstrated a median survival of 5.7 months (Burris 1997), and has been the mainstay in treating patients with PDA. The first combination regimen to demonstrate any survival benefit compared with gemcitabine alone was gemcitabine plus erlotinib, with median survival of 6.24 months versus 5.91 months for single agent gemcitabine (Moore 2007).

A meta-analysis of randomized trials by Heinemann and colleagues showed that patients with advanced pancreatic cancer and a good performance status may benefit from combination chemotherapy with gemcitabine plus a platinum agent or a fluoropyrimidine (Heinemann 2008). Multiple combination regimens are being utilized.

Recently, the regimen of 5-fluorouracil/leucovorin/irinotecan/oxaliplatin (FOLFIRINOX) compared with gemcitabine demonstrated improvement in both progression-free survival (6.4 vs. 3.3 months) and overall survival (11.1 vs. 6.8 months) for patients with a good performance status. FOLFIRINOX, however, is associated with substantial grade 3 and 4 toxicities, including diarrhea, nausea, vomiting, fatigue, neutropenia and febrile neutropenia, and cannot be given to patients >76 years of age or in some cases patients with head of the pancreas tumors (Conroy 2011, Assaf 2011).

Current treatment regimens for advanced PDA although offering modest improvements in progression-free survival (PFS) and overall survival (OS), are clearly inadequate in achieving long term survival in these patients. Additional treatment strategies are desperately needed.

Von Hoff and colleagues recently presented data supporting the use of nab-paclitaxel and gemcitabine in a phase I/II trial in patients with previously untreated advanced PDA. All patients at the recommended phase II dose (n=44) had a decrease in CA 19-9. This regimen also demonstrated an objective response rate of 48% with median survival of 12.2 months and 48% 1-year survival and 25% 2-year survival. It is speculated that reducing the dense tumor stroma, using an albumin-coated nanoparticle (nab-paclitaxel) homing to the protein SPARC (secreted protein acidic and rich in cysteine), may allow the chemotherapy to reach the tumor tissue more efficiently (Von Hoff 2011). An international phase III trial comparing this combination to gemcitabine single agent has completed accrual and results demonstrated a statistically significant improvement in overall survival for advanced pancreatic cancer patients using the gemcitabine and nab-paclitaxel over gemcitabine alone (see Appendix D).

Building on the design and mechanisms of action of the nab-paclitaxel and gemcitabine combination, this protocol introduces a third cytotoxic agent, cisplatin, to be added to this doublet. The rationale for adding cisplatin to nab-paclitaxel and gemcitabine is that in a study of 1,029 patients whose pancreatic cancer tumors were sent for molecular profiling, 57% of these tumors were negative for ERCC1, indicating sensitivity to a platinum anti-tumor agent (Von Hoff 2012). In addition to the above, in our whole genome/transcriptome sequencing analysis, we found that

abnormal repair pathways were a feature of all of the pancreatic cancers that were sequenced (Liang 2012). Cisplatin prevents cellular DNA repair by binding to and causing crosslinking of DNA, triggering apoptosis. Cisplatin has been used in other combination regimens to treat patients with PDA. For example, the cisplatin, epirubicin, 5-fluorouracil and gemcitabine (PEFG) regimen had an acceptable toxicity profile and was associated with a 24% partial response rate, 5 month progression-free survival (PFS) and 8.3 month overall survival as second line therapy (Reni 2008).

Through 2013, there had been no documented reports of the combination of cisplatin with nab-paclitaxel and gemcitabine in the treatment of any human cancer. However, cisplatin has been combined with paclitaxel and gemcitabine in the treatment of patients with locally advanced or metastatic non-small cell lung cancer (NSCLC) patients and has shown substantial antitumor activity with an acceptable safety profile. In this phase I-II study of 65 patients with advanced NSCLC, the overall response rate was 57% (Frasci 1999).

A recent pilot, phase IB/IIA study investigating the efficacy and safety of cisplatin with nab-paclitaxel and gemcitabine in 25 patients with previously untreated metastatic PDA demonstrated a 67.5% 12-month overall survival and an acceptable toxicity profile at the MTD cisplatin dose of 25mg/m2. There was a 71% overall response rate; 2 pts had a complete response (8.3%) and 15 with partial responses (62.5%). In addition, 4 patients experienced stable disease (16.7%) (Jameson, 2019). These encouraging data are the basis for this protocol.

1.1 Potential Risks of nab-Paclitaxel

The most common toxicities reported for nab-paclitaxel include myelosuppression, predominantly neutropenia, infections (24%), dyspnea (12%), peripheral neuropathy and nausea and vomiting, myalgias and arthralgias, mucositis, alopecia, transaminitis, serum creatinine elevation. Other reported infrequent toxicities include; allergic reaction, loss of appetite, diarrhea, constipation, cough, edema, fever, pruritus, hypotension, nail changes, vision changes, rash, pulmonary edema, irregular heartbeat (see Appendix D).

1.2 Potential risks of Cisplatin

The most common toxicities of cisplatin include nephrotoxicity (28-36%; acute renal failure and chronic renal insufficiency), peripheral neuropathy (dose and duration dependent), nausea and vomiting (76% to 100%), myelosuppression (25% to 30%; nadir: day 18-23; recovery: by day 39; mild with moderate doses, mild-to-moderate with high-dose therapy), liver enzymes increased (especially SGOT and bilirubin), ototoxicity (10% to 30%; manifested as high frequency hearing loss; ototoxicity is especially pronounced in children), tissue irritation (extravasation).

Other toxicities (<1%) include alopecia (mild), anaphylactic reaction, arrhythmias, arterial vasospasm (acute), blurred vision, bradycardia, diarrhea, heart block, heart failure, hemolytic anemia (acute), hemolytic uremic syndrome, hypercholesterolemia, hypocalcemia, hypokalemia, hypomagnesemia, hyponatremia, hypophosphatemia, limb ischemia (acute), mesenteric ischemia (acute), myocardial infarction, myocardial ischemia, mouth sores, neutropenic typhilitis, optic neuritis, orthostatic hypotension, pancreatitis, papilledema, phlebitis, reversible posterior leukoencephalopathy syndrome (RPLS), SIADH, stroke, thrombophlebitis, thrombotic thrombocytopenic purpura (see Appendix D).

1.3 Potential Risks of Gemcitabine

The most common toxicities reported for gemcitabine include myelosuppression, transient elevations in serum transaminases (approximately 70%), nausea and vomiting (69%), fever (41%), rash (30%), diarrhea (19%), flu syndrome, (19%), infection (16%), alopecia (15%), edema

(13%), stomatitis (11%),) neurotoxicity (mild 10%, severe <1%), mild proteinuria and hematuria; Hemolytic Uremia Syndrome (HUS) reported rarely (0.25%), dyspnea (0.2%) and serious pulmonary toxicity (0.06%). Also reported include constipation and pruritus (see Appendix D).

1.4 Potential risks of nab-Paclitaxel plus Gemcitabine

The safety of nab-paclitaxel plus gemcitabine was initially reported by Von Hoff and colleagues (Von Hoff 2011). The most common toxicities seen at the recommended phase II dose were anemia, leukopenia, neutropenia, thrombocytopenia, fatigue, alopecia, sensory neuropathy and nausea (Von Hoff 2011).

In subjects with metastatic pancreatic cancer, who received the combination of nab-paclitaxel and gemcitabine, there may be an increase of sepsis. Pneumonitis appears to occur more often (4%) when the two drugs are given together. This requires early detection and treatment as it may be life-threatening or even fatal. In addition, acute renal or kidney failure and hemolytic uremic syndrome have been reported commonly and uncommonly, respectively, in combination of nab-paclitaxel with gemcitabine.

A very rare condition known as Posterior Reversible Encephalopathy Syndrome has occurred when gemcitabine is given alone or in combination with other chemotherapy medications.

A very rare condition known as Capillary Leak Syndrome that causes leaking of fluid outside of blood vessels has occurred when gemcitabine is given alone or in combination with other chemotherapy medications.

Additional side effects observed during post-marketing surveillance of gemcitabine, not otherwise noted above include:

- vasculitis
- gangrene

Additional side effects observed during post-marketing surveillance of nab-paclitaxel, not otherwise noted above include:

- cranial nerve palsies and vocal cord paresis
- palmar-plantar erythrodysethesia syndrome
- photosensitivity reaction
- Stevens-Johnson syndrome, toxic epidermal necrolysis, erythema multiforme radiation pneumonitis, radiation recall phenomenon

Elderly

In subjects ≥ 65 years old, who received nab-paclitaxel and gemcitabine, a higher incidence of diarrhea, decreased appetite, dehydration, and epistaxis has been reported compared to subjects < 65 years old. In subjects ≥ 75 years old, a higher incidence of serious adverse reactions and adverse reactions leading to treatment discontinuation has been reported.

Adverse drug reactions reported from post-marketing experience, even though their frequency is unknown, have been similar in type and severity to those reported in nab-paclitaxel clinical trials.

1.5 Potential risks of nab-Paclitaxel, plus Cisplatin, plus Gemcitabine

The combination of nab-paclitaxel, cisplatin, and gemcitabine has not been reported. However, a similar combination of paclitaxel, cisplatin, and gemcitabine given on days 1 and 8 every 21 days studied by Frasci, et al, (see section 1.0), showed anticipated toxicities to include myelosuppression, neutropenia, neuropathy, nephrotoxicity diarrhea, vomiting, fatigue, musculoskeletal pain and rhinorrhagia. In that study, grade 2 neurotoxicity was seen in 8 patients (10.6%), grade 3 sensory and motor neuropathy was seen in one patient (1.3%). Mild or moderate nephrotoxicity occurred in a total of 7 patients (9.3%) (Frasci 1999).

1.6 Study and Dose Rationale

This is a phase II open-label, study evaluating the efficacy and safety of nab-paclitaxel 125mg/m^2, cisplatin 25 mg/m^2, and gemcitabine 1000 mg/m^2, all administered intravenously (IV) on Days 1 and 8 every 21 days until development of toxicity that is unacceptable in the opinion of the patient or the Investigator or upon disease progression. The doses of the nab-paclitaxel and gemcitabine are taken from the phase I/II trial (Von Hoff 2011). Building upon this two drug foundation, a phase Ib/II pilot trial investigated the safety and efficacy of nab-paclitaxel and gemcitabine plus cisplatin by Jameson, et al. In 24 evaluable patients (n=25), a 71% overall response rate was noted with 2 complete responses, 15 partial responses, 4 stable disease and 3 patients with progressive disease. The median overall survival rate was 16.4 months (95% CI 10.2, 25.3) with 16 (64%) patients alive at 1 year, 10 (40%) alive at 2 years, 4 (16%) at 3 years, and 1 (4%) patient alive at 4 + years. Three patients remain alive as of last contact 12/05/18 (36-59.7 months OS). Median PFS was 10.1 months (95% CI, 6.0 -12.5). The MTD of cisplatin was determined to be 25mg/m^2. The dosing schedule of the 3 drug combination is taken from the aforementioned lung cancer study (Liang 2012).

1.7 Sequence of Drug Administration and Rationale

The sequence of drug administration is nab-paclitaxel, then cisplatin, then gemcitabine. After adequate hydration (see Section 4.1), Nab-paclitaxel is given first, targets SPARC (secreted protein acidic and rich in cysteine) in tumor cells and may be taken up by the process of macropinocytosis. Then the cisplatin is given. Gemcitabine is given last because nab-paclitaxel decreases cytidine deaminase which potentiates gemcitabine activity (less degradation of gemcitabine by the enzyme).

1.8 Quality of Life and Pain Assessments

Two well-known tools will be utilized to assess participant's self-reported quality of life and pain levels during this study. The MD Anderson Symptom Inventory (MDASI-GI) and Brief Pain Inventory (BPI) are valid, reliable, and sensitive instruments for assessing the severity of symptoms and their interference in patients' daily functioning. The MDASI asks patients to rate the worst severity of symptoms during the past 24 hours (0 not present to 10 as bad as you can imagine). It also rates the extent to which symptoms interfere with various aspects of life (e.g., general activity, mood, relations with other people, and enjoyment of life) using a scale ranging from 0 (did not interfere) to 10 (interfered completely). The BPI uses four pain severity items rated with a 0 to 10 numeric rating scale (NRS); the interference scale is similar to the MDASI (0 = no interference to 10 = interferes completely). Internal consistency measures (reliability) in prior studies were high at 0.80 or more (Cronbach alpha) for the BPI and MDASI (Atkinson et al, 2010; Cleeland et al, 2000; Janjan et al, 2007; Wang et al, 2010).

Instruments will be administered at screening, at the beginning of each cycle throughout treatment and at end of study. Please see Appendix (E) and (F) for information on instruments.

1.9 Tumor Analysis

Pancreatic adenocarcinoma tumors are known to have numerous driver mutations along with DNA repair problems (Barrett 2017). The identification of patterns of vulnerable target mutations would be very useful in further understanding not only tumor biology but response to therapy and treatment selection. Work done by Alexandrov and colleagues has illustrated a pattern of DNA mutations labeled, 'Signature 3" (Alexandrov 2013). This signature of tumor mutations in pancreatic cancer includes the family of BRCA mutations. Interestingly, in a sample of pancreatic cancer patients, all responders of platinum therapy exhibited this mutation signature *including* those that lacked defects in BRCA1 or BRCA2 (Alexandrov 2015). The correlation of the presence of this pattern on tumor cells with response to our "Triplet" platinum based therapy could potentially lead to improved selection of patients who would be respond to this therapy. Therefore, further study is warranted. We have included the tumor analysis of the signature 3 pattern of mutation in this study.

The genomic analysis of fresh tissue or archived tumor paraffin blocks will be used to look for copy number variants (CNV), and gene mutations including Signature 3. The genes and pathways targeted by selected mutations in both responder and non-responder patients will provide highly favorable candidate markers and targets that can be advanced in future studies for improved patient care. Preferably, this analysis will be conducted on the metastatic tumor tissue when available.

Next generation sequencing will be conducted on the samples. All libraries and sequencing will be done in the Mayo Clinic Medical Genome Facility using their established protocols. Each tumor will then be assessed for the presence of mutational signatures that predict response to DNA damage and repair targeting therapies. The primary and secondary analyses of sequencing data will be done in collaboration with Mayo Clinic Bioinformaticians.

Barrett Laboratory:
Mayo Clinic Arizona
Mayo Collaborative Cancer Research Building, Cr2-111
Attn: Michael Barrett, PhD
13208 E. Shea Blvd, Suite 100
Scottsdale, AZ 85259

Instructions for shipment of samples to the analysis laboratory are detailed in the Trial Lab Manual.

2 OBJECTIVES

2.1 Primary Objective

The primary objective of this study is to evaluate the 12-month overall survival rates in patients with metastatic PDA treated with nab-paclitaxel plus cisplatin plus gemcitabine.

2.2 Secondary Objectives

The secondary objectives of this study are to:

- Evaluate the safety of cisplatin plus nab-paclitaxel plus gemcitabine

- Evaluate the efficacy of cisplatin plus nab-paclitaxel plus gemcitabine based on complete response rate (RECIST 1.1), disease control rate at 9 weeks, and change in CA 19-9 (or CA 125, or CEA if not expressers of CA 19-9)

- Evaluate the participant's self-reported quality of life and pain levels during this study

- To expand understanding of the molecular mechanisms of treatment response

2.3 Overview

This is a phase II open-label study evaluating the efficacy and safety of nab-paclitaxel cisplatin, and gemcitabine in patients with metastatic pancreatic ductal adenocarcinoma.

An individual cycle of therapy will be defined as Days 1 and 8 every 21 days. Multiple cycles may be administered until the patient is withdrawn from therapy.

Overall response rates as well as individual categories of response (CR, PR, SD, and PD) will be determined using RECIST 1.1 (Frese 2012). Time-to-event endpoints, including PFS and OS will be assessed using the Kaplan-Meier method (Kaplan 1958). Evaluation of stable disease at 9 weeks will also be assessed. Toxicity (adverse events) will be recorded using the NCI CTCAE, version 5.0 (see Appendix C).

2.4 Primary Endpoints

- To evaluate the 12-month overall survival rates in patients with metastatic PDA.

2.5 Secondary Endpoints

To evaluate the following:

- Treatment-related toxicities.

- Complete response rate as defined by CT scan using RECIST 1.1 criteria and CA 19-9 (or CA 125, or CEA if not expressers of CA 19-9) down to normal limits (from at least > 2X ULN).

- Disease control rate (CR, PR and SD at 9 weeks) in patients with metastatic PDA.

- Change in CA 19-9 (or CA 125, or CEA if not expressers of CA 19-9) in this patient population.

- Rates of normalization of CA 19-9 (or CA 125, or CEA if not expressers of CA 19-9).

- Participant's self-reported quality of life and pain levels during this study utilizing the MD Anderson Symptom Inventory (MDASI-GI) and Brief Pain Inventory (BPI).

- To expand understanding of the molecular mechanisms of treatment response by performing genomic characterization on patient's tumor biopsies. Our hypothesis is that the PDAs that respond to the nab-paclitaxel, cisplatin, gemcitabine regimen will have distinct mutational and CNV signatures.

3 STUDY POPULATION

3.1 Patient Selection and Study Duration

Up to 50 patients will be recruited from up to 4 study centers in the United States with the goal of 42 evaluable patients. The expected duration of this study is 36 months; 24 months for accrual and 12 months follow-up. Enrollment into the screening or treatment phase of the study will be stopped when the anticipated or actual patient numbers have been achieved across all study sites or in the event that any of the stopping rules as defined in Section 8.5.1 are met.

3.2 Inclusion Criteria

Patients must meet the following criteria to be included in the study:

1. Age ≥18 years of age; male or female.

2. Histologically or cytologically confirmed metastatic pancreatic ductal adenocarcinoma.

3. Capable of providing informed consent and complying with trial procedures.

4. Karnofsky Performance Status (KPS) of ≥ 70%.

5. Life expectancy ≥ 12 weeks.

6. Measurable tumor lesions according to RECIST 1.1 criteria.

7. < Grade 2 pre-existing peripheral neuropathy per NCI CTCAE, Version 5.0.

8. Patient has acceptable coagulation status as indicated by an INR ≤1.5 x ULN. Patients on anticoagulation can be included at the discretion of the investigator.

9. Patients must have normal organ and marrow function as defined below:

   - Absolute neutrophil count ≥1,500/mm^3
   - Platelet concentration ≥100,000/mm^3 with no platelet transfusions within 7 days prior to laboratory sample
   - Hemoglobin > 9.0g/dL
   - Hematocrit level > 27%
   - Total bilirubin within 1.25 times institutional upper limit of normal (ULN)
   - Alanine aminotransferase (ALT) and AST ≤ 10 × institutional ULN
   - Serum creatinine <1.5 mg/dl

10. Females of child-bearing potential (defined as a sexually mature woman who (1) has not undergone hysterectomy [the surgical removal of the uterus] or bilateral oophorectomy

[the surgical removal of both ovaries] or (2) has not been naturally postmenopausal for at least 24 consecutive months [i.e., has had menses at any time during the preceding 24 consecutive months]) must:

a. Either commit to true abstinence* from heterosexual contact (which must be reviewed on a monthly basis), or agree to use, and be able to comply with, effective contraception without interruption, 28 days prior to starting IP therapy (including dose interruptions), and while on study medication or for a longer period if required by local regulations following the last dose of IP; and

b. Have a negative serum pregnancy test (β -hCG) result at screening and agree to ongoing pregnancy testing during the course of the study, and after the end of study therapy. This applies even if the subject practices true abstinence* from heterosexual contact.

11. Male subjects must practice true abstinence* or agree to use a condom during sexual contact with a pregnant female or a female of childbearing potential while participating in the study, during dose interruptions and for 6 months following discontinuation from study treatment, even if he has undergone a successful vasectomy.

* True abstinence is acceptable when this is in line with the preferred and usual lifestyle of the subject. [Periodic abstinence (e.g., calendar, ovulation, symptothermal, post-ovulation methods) and withdrawal are not acceptable methods of contraception].

3.3 Exclusion Criteria

Patients meeting the following criteria will not be enrolled:

1. Patients must have received no previous radiotherapy, surgery, chemotherapy or investigational therapy for the treatment of metastatic disease. Prior treatments in the neoadjuvant and/or adjuvant setting with gemcitabine and/or 5-FU based therapies or gemcitabine and/or 5FU administered as a radiation sensitizer are allowed, provided at least 6 months have elapsed since completion of the last dose and no lingering toxicities are present.

2. Palliative surgery and/or radiation treatment less than 4 weeks prior to initiation of study treatment.

3. Exposure to any investigational agent within 4 weeks prior to initiation of study treatment.

4. Evidence of central nervous system (CNS) metastasis (negative imaging study, if clinically indicated, within 4 weeks of Screening Visit).

5. History of other malignancies (except cured basal cell carcinoma, superficial bladder cancer or carcinoma *in situ* of the cervix) unless documented free of cancer for ≥5 years.

6. Current, serious, clinically significant cardiac arrhythmias as determined by the investigator.

7. History of HIV infection.

8. Active, clinically significant serious infection requiring treatment with antibiotics, anti-virals or anti-fungals.

9. Major surgery within 4 weeks prior to initiation of study treatment.

10. Any condition in the opinion of the principal investigator that might interfere with the patient’s participation in the study or in the evaluation of the study results.

11. Any condition in the opinion of the principal investigator that is unstable and could jeopardize the patient’s participation in the study.

3.4 Patient Enrollment

All patients must have a signed and dated current, IRB approved informed consent and HIPAA Authorization prior to any study specific screening procedures being performed.

A trial and site specific screening number will be assigned by the site when the patient signs the Informed Consent Form. Once all screening procedures have been completed and laboratory values have been reported, the investigator will register the patient into the study as follows:

1. Completion of all data entry into the required screening eCRFs
2. Provide HRI with the completed patient eligibility forms signed by the Investigator to HRI via fax or email.

HRI will review the screening eCRFs within 48 hour and confirm eligibility of the patient and assign the trial specific patient enrollment number.

If the patient is determined NOT eligible for the study, the patient will be determined a screen failure and the reason noted on the patient eligibility form and sent to the Investigator for the study records. The Investigator, or delegated study team member, is responsible for entering all patients consented to the study in the EDC, including screen failures.

3.5 Patient Discontinuation

Patients will be discontinued from the treatment under the following circumstances:

1. Disease progression.

2. Patient’s physician considers a change of therapy would be in the best interest of the patient.

3. Patient requests discontinuation.

4. Continued unacceptable toxicities despite optimal treatment or dose reduction.

5. Patient becomes pregnant or fails to use adequate birth control (for those patients who are fertile).

6. Need for any treatment not allowed by the protocol.

7. Non-compliance.

4 STUDY TREATMENT

4.1 Administration and Dosing

Treatment will be administered by qualified and trained site personnel in a hospital, clinic, or other outpatient setting appropriate for chemotherapeutic infusions. Investigator sites will utilize their own commercial supply for this trial.

No investigational or commercial agents or therapies other than those described may be administered with the intent to treat the patient's malignancy.

The solution for infusion will be prepared at each investigational site. Detailed guidelines for the preparation and administration of nab-paclitaxel, cisplatin and gemcitabine are provided in Appendix D. The order of infusion with premedication is as follows:

- Pre cisplatin hydration: 0.9% Sodium Chloride Injection 1000 mL with Magnesium Sulfate 2 grams IV infusion over 2 hours on days 1 and 8 repeated every 21 days.

- Palonosetron (Aloxi®) 0.25 mg IV, fosaprepitant (Emend®) 150 mg IV and dexamethasone 12 mg IV, or equivalent antiemetic regimen within 30 minutes prior to treatment on days 1 and 8, repeated every 21 days Patients will continue oral antiemetic prophylaxis at home with ondansetron 8mg bid and dexamethasone 4mg bid for 2 days after chemotherapy. The type of antiemetic prophylaxis used can vary based on institutional procedures.

- Nab-paclitaxel 125mg/m^2 over 30 minute IV infusion on days 1 and 8 repeated every 21 days. Following administration, the intravenous line should be flushed with sodium chloride 9 mg/ml (0.9%) solution for injection to ensure complete administration of the complete dose, according to local practice. followed by:

- Cisplatin 25mg/m^2 in 500 mL of NS (the investigator may reduce the volume to 250 mL of NS if clinically indicated) over 60 minute IV infusion on days 1 and 8 repeated every 21 days followed by:

- Gemcitabine 1000mg/m^2 in 500 mL of NS (the investigator may reduce the volume to 250 mL of if clinically indicated) over 30 minute IV infusion on days 1 and 8 repeated every 21 days.

- Post cisplatin hydration: IV fluids up to 1000 mL (with additives as clinically indicated) IV given as infusion on days cisplatin is administered on days 1 and 8 repeated every 21 days. May start at the same time as the gemcitabine infusion. Additional post-cisplatin hydration of 1 liter of IV fluids on days 2 & 9 required for Cycles 1- 3. Volume may be reduced to 500 ml at the Investigator's discretion. Note: If the cisplatin is held in any cycle, the additional hydration on day of chemotherapy or day after would not be needed.

In the event of extravasation during the infusion of nab-paclitaxel, cisplatin and gemcitabine, the infusion should be immediately terminated and patients treated according to local site protocols. The infusion should then be restarted in another vein.

4.2 Body Surface Area Calculation

The calculation of the dose of cisplatin, gemcitabine, and nab-paclitaxel will be based on the patient's body surface area (BSA) using the Mosteller formula (Verbraeken 2006). The BSA will be calculated before each new cycle, based on the actual height and weight of the patient. If there has been a > 10% weight change from baseline (Cycle 1 Day 1), the drug doses will be recalculated based on the new BSA value. Doses are rounded to the nearest whole milligram.

4.3 Dose Modification for Toxicity

Toxicities will be graded using the NCI CTCAE v5.0 (see Appendix C). If toxicity occurs during or after any treatment cycle, the toxicity will be graded and appropriate supportive care treatment may be administered to decrease the signs and symptoms (e.g. antiemetics, antidiarrheals, antipyretics, antihistamines).

Doses of nab-paclitaxel and gemcitabine may be reduced in individual patients in accordance with the schedule in Table 1. In general, doses that have been reduced for toxicity will not be escalated back to the starting level. Growth factors may be used to treat hematologic toxicity and will not constitute a dose reduction.

A maximum of a 3-week treatment delay is permitted to allow recovery of toxicities or other concurrent illness that prevents the patient from receiving chemotherapy.

Table 1. Dose Reduction Schema

Dose Level	nab-Paclitaxel (mg/m2)	Cisplatin Dose 1	Gemcitabine (mg/m2)
Level - 0 (baseline)	125mg/m2	No change	1000mg/m2
Level -1	100mg/m2	No change	800mg/m2
Level -2	75mg/m2	No change	600mg/m2

4.3.1 Hematological Toxicity

In the event dose modifications are required at the beginning of a cycle or within a cycle due to hematologic toxicities, doses of nab-paclitaxel, cisplatin, and gemcitabine may be adjusted as detailed in Table 2 and Table 3.

During Cycle 1, if the patient experiences a grade 4 hematologic adverse event related to chemotherapy as determined by the investigator, dose reduce to Level -1 for Cycle 2.

Dose Modifications at Day 1

Table 2. Dose Modifications for Day 1 of Each Cycle (Hematologic Toxicity)

ANC		Platelets	Timing
≥ 1.5 x 10^9/L	And	≥ 100 x 10^9/L	Treat on time
< 1.5 x 10^9/L	Or	< 100 x 10^9/L	Delay by 1 week intervals until recovery

Dose Adjustments within a Treatment Cycle

In the event that patients must have treatment delayed within a treatment cycle due to hematologic toxicities, those doses held during a cycle will not be made up. Dose modifications due to hematologic toxicity (as represented by the blood counts and toxicities, below) within a treatment cycle should be adjusted as outlined in Table 3.

Table 3. Dose Modification for Day 8 of Each Cycle (Hematologic Toxicity)*

Day 8 Laboratory Results	Day 8 Nab-paclitaxel	Day 8 Cisplatin	Day 8 Gemcitabine
ANC > 1000 and Platelets ≥ 75,000	100%	100%	100%
ANC 500-1000[a] or Platelets 50,000-74,999	Decrease dose by 1 level (treat on time)	100%	Decrease dose by 1 level (treat on time)
ANC < 500 or Platelets < 50,000	Hold	Hold	Hold
Febrile Neutropenia (Grade 3 or 4)[b]	Hold. Upon resuming dosing, decrease to next lower dose level and do not re-escalate throughout the rest of treatment	Hold	Hold. Upon resuming dosing, decrease to next lower dose level and do not re-escalate throughout the rest of treatment
Recurrent Febrile Neutropenia (Grade 3 or 4)	Decrease 2 dose levels (to 75 mg/m2) and do not re-escalate throughout the rest of treatment	Hold	Decrease 2 dose levels (to 600 mg/m2) and do not re-escalate throughout the rest of treatment.

* see Table 1 for dose reduction schedule

[a] If patients do not experience resolution of neutropenia within 21 days, despite uninterrupted G-CSF treatment, study treatment will be discontinued.

[b] Febrile patients (regardless of neutrophil count) should have their chemotherapy treatment interrupted (see Section 4.3.2.9). A full sepsis diagnostic work-up should be performed while continuing broad spectrum antibiotics. If cultures are positive, the antibiotic may or may not be changed, depending on the sensitivity profile of the isolated organism. Patients with persisting fever after 3 weeks, despite uninterrupted antibiotic treatment, will discontinue study treatment. Febrile neutropenic patients can also receive G-CSF, in addition to antibiotic treatment, to hasten the resolution of their febrile neutropenia (following current institutional guidelines). In all cases, blood counts must have returned to baseline levels before resuming chemotherapy treatment.

4.3.2 Non-hematological Toxicity

Dose reductions for non-hematologic toxicity that occur despite adequate background medical therapy should be undertaken in accordance with Table 4. Cisplatin is addressed in sections 4.3.2.1, 4.3.2.2, and 4.3.2.7

Table 4. Nab-paclitaxel and gemcitabine Dose Modifications for Day 1 of Each Cycle (Non-hematologic Toxicity)*

Non Hematologic Toxicity and/or Dose Hold with Previous Cycle	
**Toxicity/dose held**	***nab*-paclitaxel + gemcitabine dose this cycle**
Grade 0, 1 or 2 toxicity	Same as Day 1 previous cycle (except for Grade 2 cutaneous toxicity where doses of nab-paclitaxel and gemcitabine should be reduced to next lower dose level: please refer to Section 4.3.2.3)
Grade 3 toxicity[a,c]	Decrease *nab*-paclitaxel and gemcitabine to next lower dose level[a]
Grade 4 toxicity[b]	Off protocol treatment[b]
Dose held in 2 previous consecutive cycles	Decrease nab-paclitaxel and gemcitabine to next lower dose level and continue throughout the rest of treatment

* Excluding peripheral neuropathy (section 4.3.2.1) and nephrotoxicity (section 4.3.2.2).
[a] If the toxicity only affects neuropathy, then only *nab*-paclitaxel should be reduced (please see Section 0).
[b] Pulmonary embolism (a Grade 4 toxicity in the CTCAE tables) if mild or asymptomatic, will be exempt from this requirement (please see Section 4.3.2.5).
[c] Excluding electrolyte abnormalities per judgment of the physician/investigator.

Table 5. Nab-paclitaxel and gemcitabine Dose Modifications Day 8 Each Cycle (Non-hematological Toxicity)

CTC Grade	Percent of Day 1<br>***nab*-paclitaxel + gemcitabine Dose**
**Grade 0-2**	100%[a]
**Grade 3+**	Hold treatment until resolution to ≤ Grade 1 [b,c].

[a] Except for cutaneous toxicity: refer to Section 4.3.2.3.
[b] Pulmonary embolism (a Grade 4 toxicity in the CTCAE tables) if mild or asymptomatic, will be exempt from this requirement (please see Section 4.3.2.5).
[c] Excluding electrolyte abnormalities per judgment of the physician/investigator.
Please note when one drug is discontinued then it will be a matter of patient situation and clinical judgement as to whether some or all of the other agents are discontinued.

4.3.2.1 Peripheral Neuropathy

Cisplatin and nab-paclitaxel treatment should be withheld in patients who experience ≥ Grade 3 peripheral neuropathy. Gemcitabine administration can continue during this period. Cisplatin may be resumed at the same dose and nab-paclitaxel treatment may be resumed at the next lower dose level in subsequent cycles after the peripheral neuropathy improves to ≤ Grade 1. The time to resolution to Grade ≤ 1 should be the adverse event duration used for adverse event reporting.

Patients experiencing peripheral neuropathy that requires a delay in scheduled cisplatin and nab-paclitaxel dosing for ≥ 21 days will discontinue study treatment, will have the end of treatment procedures completed per Section 5.4, and will continue to be followed for survival per Section 5.5.

4.3.2.2 Nephrotoxicity

Cisplatin (cisplatin injection) produces cumulative nephrotoxicity which is potentiated by aminoglycoside antibiotics (see Appendix D). The serum creatinine, BUN, creatinine clearance,

and magnesium, sodium, potassium, and calcium levels should be measured prior to initiating therapy, and prior to each subsequent course. Cisplatin should not be given unless serum creatinine is < 1.5 mg/dl.

4.3.2.3 Cutaneous Toxicity

Patients who develop Grade 2 or 3 cutaneous toxicity should have their nab-paclitaxel and gemcitabine dose reduced to the next lower dose level as per Table 1. If the patient continues to experience these reactions, despite dose reduction, treatment should be discontinued. Patients who develop Grade 4 cutaneous toxicity should have treatment discontinued.

4.3.2.4 Gastrointestinal Toxicity

If Grade 3 mucositis or diarrhea occurs, all 3 study drugs should be withheld until resolution to ≤ Grade 1, then reinstituted at the next lower dose level as per Table 1. Patients who develop Grade 4 mucositis or diarrhea should have treatment discontinued.

4.3.2.5 Pulmonary Embolism

Asymptomatic or clinically mild pulmonary embolism can be treated with low-molecular weight heparin without interruption of therapy. Moderate to severe pulmonary embolism will require permanent discontinuation of treatment.

4.3.2.6 Interstitial Pneumonitis

Pulmonary toxicity has been reported for both gemcitabine and paclitaxel. Epidemiology reports show that gemcitabine monotherapy is weakly associated with lung toxicity. A retrospective review (Meadors 2006) of pooled clinical trial data of 4,448 patients with mixed cancer indications reported an incidence of dyspnea of 0.2% and serious pulmonary toxicity of 0.06%. Paclitaxel monotherapy is weakly associated with lung toxicity (Rowinsky 1995). Dyspnea with bronchospasm has been reported in 0.3 to 0.9%, with 30% of type 1 hypersensitivity reactions. Combination chemotherapy of gemcitabine and paclitaxel shows a higher incidence of pneumonitis (4%) compared to either drug alone. This requires early detection and treatment as it may be life-threatening or even fatal.

During study participation, patients should be carefully monitored for signs and symptoms of pneumonitis (i.e. episodes of transient or repeated dyspnea with unproductive persistent cough or fever) and, if observed, immediate clinical evaluation and timely institution of appropriate management (emphasizing the need for corticosteroids if an infectious process has been ruled out as well as appropriate ventilation and oxygen support when required). Administration of study drugs will be permanently discontinued upon making a diagnosis of interstitial pneumonitis.

<u>Prevention, Surveillance and Management of Interstitial Pneumonitis</u>

- During study treatment, episodes of transient or repeated dyspnea with unproductive persistent cough or fever should be paid attention to. Radiographic evaluation with chest X-rays and CT scans (normal or high resolution) may be indicated to look for infiltrates, ground-glass opacities or honeycombing patterns. Pulse oximetry and pulmonary function tests can show respiratory and ventilation compromise.

- Infections should be ruled out with routine immunological/ microbiological methods. Transbronchial lung biopsy is not recommended, given its limited value and risk of pneumothorax and hemorrhage, and should be reserved for cases with unclear etiology.

- Study drug administration should be interrupted upon diagnosis of interstitial pneumonitis and patients permanently discontinued from further study drug treatment. After ruling out an infectious etiology, intravenous high-dose corticosteroid therapy and secondary pathogen coverage should be instituted without delay. Patients with an added immunological component may also require immune modulation with azathioprine or cyclophosphamide. Appropriate ventilation and oxygen support should be used when required.

4.3.2.7 Hypersensitivity Reactions

Hypersensitivity reactions are not usually expected with cisplatin, *nab*-paclitaxel or gemcitabine. If they do occur, minor symptoms such as flushing, skin reactions, dyspnea, hypotension, or tachycardia may require temporary interruption of the infusion. However, severe reactions, such as hypotension requiring treatment, dyspnea requiring bronchodilators, angioedema, or generalized urticaria require immediate discontinuation of the offending agent and aggressive symptomatic therapy. Patients who develop a severe hypersensitivity reaction should not be re-challenged.

4.3.2.8 Prophylaxis use of Colony Stimulating Factors

Based on the ASCO guidelines- Appendix G (Smith 2015) for use of granulocyte colony stimulating factors, (G-CSF) for regimens with at least a 20% risk of febrile neutropenia, pegfilgrastim will be administered subcutaneously on day 9 of each treatment cycle. Neulasta OnPro® may be given on **Day 8** if patient is not returning for Day 9 hydration.

Filgrastim may be administered at the Investigator's discretion, e.g. after day 1 in cycles 2 and beyond to prevent neutropenia on day 8.

G-CSF may also be given according to institutional guidelines for the treatment of neutropenic fever or infections associated with neutropenia and for the prevention of febrile neutropenia in patients with an ANC < 500 cells/µL. Patients not experiencing resolution of neutropenia within 21 days, despite uninterrupted G-CSF treatment, will discontinue study treatment.

4.3.2.9 Prophylaxis against Sepsis

Due to the incidences of non-neutropenic sepsis, at the first occurrence of fever ≥ 38.5°C (regardless of neutrophil count), institution of ciprofloxacin (500 mg orally, twice daily)—or amoxicillin/clavulanate (Augmentin®, 500 mg orally, 2-3 times daily) in patients with allergy to fluoroquinolones—should be initiated. On their first visit, patients should be provided with enough ciprofloxacin (or the alternative antibiotic) for use at home, and they should be instructed to begin taking it when they first record a temperature of ≥ 38.5 °C (or if they feel they are developing a fever and a thermometer is not available). They should also immediately contact their physician for guidance on where to go for blood counts and to be evaluated for sepsis as soon as possible. Hospitalization or evaluation in the emergency room may be required depending on the clinical presentation. If hospitalization is required, please refer to Section 7.2 of this protocol to report the event as a Serious Adverse Event (SAE).

4.4 Intended Dose Delays

Intended cycles may be delayed for non-toxicity reasons for up to 7 days (for reasons such as scheduling conflicts), but only with documentation and explanation in the CRF. Dosing on day 8 will not be delayed beyond +1 day. If an individual dosing day needs to be delayed beyond +1 day, that particular dose is not to be administered and will be considered a missed dose.

4.5 Concomitant Therapy

Necessary supportive measures for optimal medical care may be given throughout the study, including IV antibiotics to treat infections, blood components, and antiemetics. Additional care will be administered as indicated by the treating physician and the patient's medical need. No concomitant cytotoxic therapy, whether conventional or investigational, will be allowed during this study. All concomitant medications and supportive therapy must be recorded on the appropriate CRF.

Radiotherapy is not allowed while the patient is enrolled in this study.

Routine **prophylactic use** of a colony-stimulating factor (G-CSF) should be used according to the American Society of Clinical Oncology guidelines (Appendix G) and recorded in the EDC.

Erythropoietin is permitted if clinically indicated.

4.6 Concomitant Therapies Requiring Caution

Cisplatin nephrotoxicity may be exacerbated by treatment with other nephrotoxic drugs (e.g. aminoglycoside antibiotics, non-steroidal anti-inflammatory drugs). Caution with use of other nephrotoxic drugs.

If anticoagulation with warfarin is necessary, frequent monitoring of prothrombin time and the International Normalized Ratio (INR) is recommended.

The use of vitamins or supplements that have reported use for the treatment or prevention of cancer, or that may interact with any of the study medications is not permitted.

5 STUDY ASSESSMENTS

5.1 Laboratory Assessments

All hematology, blood chemistries, urinalyses, and serum or urine pregnancy tests (if applicable) will be performed by the local laboratory for each investigational site.

Prior to study enrollment, each patient will have the following assessments completed (see Appendix A).

5.2 Screening (Within 21 Days Prior to First Dose on C1/D1)

1. Written informed consent to be obtained prior to any study specific screening procedures being performed.*

2. Review inclusion/exclusion criteria

3. Medical history including concurrent baseline conditions (using NCI CTCAE version 5.0; Appendix C), prior cancer therapy (including documentation of prior surgery, adjuvant or neoadjuvant chemotherapy and radiotherapy)*

4. Complete physical examination including height (cm) and weight (kg)*

5. Karnofsky Performance Status (KPS) (see Appendix B)*

6. Vital signs (blood pressure, pulse, respiratory rate, and temperature)*

7. Computed tomography (CT) / magnetic resonance imaging (MRI) scan to document disease status (including chest, abdomen, pelvis, and other regions as clinically indicated. In addition, brain scan is required to exclude brain metastases if clinically indicated only. If a CT scan was taken within 28 days prior to first dose, a new scan is not necessary. (see RECIST 1.1 criteria in Section 6)

8. Electrocardiogram (ECG)*

9. Complete blood count (CBC) with differential and platelet count*

10. Serum chemistries (for hepatic and renal function tests) including: blood urea nitrogen (BUN), phosphorus, magnesium, creatinine, creatinine clearance (the Cockcroft-Gault equation may be used to calculate the creatinine clearance at any of the time points in this protocol), total protein, albumin, calcium, glucose, total bilirubin, alkaline phosphatase (ALP), aspartate aminotransferase (AST), alanine aminotransferase (ALT), and electrolytes (chloride, sodium, potassium, and bicarbonate)*

11. CA 19-9 (or CA 125, or CEA if not expressers of CA 19-9)*

12. PT/INR*

13. Urinalysis (lab): protein, specific gravity, glucose, and blood*

14. Serum pregnancy test (if applicable)

15. Concomitant medication notation (to include all medications taken within 30 days prior to study enrollment)

16. Patient to complete MDASI-GI and BPI questionnaires

* These procedures, *if done prior to obtaining consent* as usual medical care, can be accepted as screening procedures if completed within 21 days prior to cycle 1, day 1.

Once eligibility is confirmed by the Investigator, the site is to register the patient following the procedures outlined in Section 3.4.

5.3 On-Study Assessments

Patients must begin Cycle 1 within 21 days of signing the informed consent document and after the screening assessments. Treatment will be administered by qualified and trained site personnel in a hospital, clinic, or other out-patient setting appropriate for chemotherapeutic infusions. All assessments should be performed preferentially on the same day of the study visit but may be performed within 1 day prior if medical or scheduling conditions require.

5.3.1 Tissue Analysis

Once eligibility is confirmed, and the patient is enrolled in the study, the study site is to obtain, if available, the archived paraffin block of tumor specimen, metastatic tumor sample is preferred, for tissue analysis as detailed in Section 1.9. Availability of archival tissue is not required for patient to be deemed eligible for enrollment into the study.

Additionally patients will be provided the option to consent to provide fresh frozen tissue samples from a newly obtained core or excisional biopsy if performed recently (within the last 30 days prior to Cycle 1/Day 1) and during their participation in the study should they be scheduled for a biopsy as part of their standard of care during treatment or at the time of disease progression. The patient should be consented with the tissue collection informed consent prior to the biopsy. If the patient consents to this optional tissue collection, the fresh tissue sample should be collected per the instructions in the laboratory manual for tissue analysis as detailed in Section 1.9.

Any samples remaining after the trial specified analyses are completed will be transferred to and stored at HonorHealth for future research purposes at HonorHealth's discretion. This includes the original specimen collected from the patient tumor tissue, as well as derivatives created from the original specimen (DNA, RNA, blocks or slides). Documentation of the transfer of all specimens will be recorded on the Sample Transfer Form- refer to Trial Laboratory Manual

5.3.2 Day 1 of each cycle

All assessments should be performed preferentially on the same day of the study visit but may be performed within 1 day prior to study dose administration if medical or scheduling conditions require.

- Inclusion/exclusion review (Cycle 1 only)
- Medical history review (Cycle 1 only)
- Directed physical exam
- Vital Signs (blood pressure, pulse, respiratory rate, and temperature) to be collected prior to treatment.
- Measurement of weight (kg) and BSA calculation prior to dosing (BSA will be calculated before each new cycle, based on the actual height and weight of the patient. After Cycle 1, if there has been a > 10% weight change from baseline (Cycle 1 Day 1), the drug doses will be recalculated based on the new BSA value.) (See Section 4.2).
- Karnofsky Performance Status (KPS) (see Appendix B)
- Hematology: CBC with differential and platelet count (see Section 5.2)
- Serum chemistries (see Section 5.2)
- CA 19-9 (or CA 125, or CEA if not expressers of CA 19-9)
- PT/INR required only If patient is on anticoagulation with warfarin.
- Urinalysis (see Section 5.2)
- Serum Pregnancy if applicable. Negative result must be confirmed prior to dosing.
- AEs using the NCI CTCAE (see Appendix C)
- Concomitant medication notation
- Patient to complete MDASI-GI and BPI questionnaires

- Administration of triple therapy as per Section 4.1

5.3.3 Day 8 of each cycle

All assessments should be performed preferentially on the same day of the study visit but may be performed within 1 day prior to study dose administration if medical or scheduling conditions require.

- Directed physical exam
- Vital Signs (blood pressure, pulse, respiratory rate, and temperature) to be collected prior to treatment.
- Measurement of weight (kg)
- Karnofsky Performance Status (KPS) (see Appendix B)
- Hematology: CBC with differential and platelet count
- Serum chemistries (see Section 5.2)
- AEs using the NCI CTCAE (see Appendix C)
- Concomitant medication notation
- Administration of triple therapy as per Section 4.1

5.3.4 Day 15 of each cycle

Cycle 1 day 15 clinic visit can be conducted by telemedicine (video teleconference or phone call). All assessments should be performed preferentially on the same day of the study visit but may be performed within 1 day prior if medical or scheduling conditions require. Lab tests on day 15 of each cycle can be done at a local lab if the patient is unable to visit the site. Lab results are to be available to the investigator within 1 day to allow evaluation of potential adverse events and patient safety.

- Directed physical exam (not required on Day 15 of cycles 2+)
- Vital Signs (blood pressure, pulse, respiratory rate, and temperature) to be collected prior to treatment. Note: not required on Day 15 of cycles 2+
- Karnofsky Performance Status (KPS) (see Appendix B) (not required on Day 15 of cycles 2+)
- Hematology: CBC with differential and platelet count
- Serum chemistries (see Section 5.2)
- AEs using the NCI CTCAE (see Appendix C)

- Concomitant medication notation

5.3.5 Tumor Assessments every three cycles (Prior to Cycles 4, 7, 10, etc.)

- In order to more precisely determine time to progression, the investigator is encouraged to obtain radiological assessments earlier if there is a strong clinical suspicion of disease progression, in order to either confirm or refute the clinical impression.
- Reassessment of the extent of tumor should be made by the same imaging methods used to establish baseline tumor measurements.

5.4 End of Treatment

When the patient completes all cycles of study medication, or withdraws from treatment prior to completing all cycles, the following assessments will be performed 14-28 (+/- 2) days after completing the last dose of study medication:

- Directed physical exam, if deemed necessary
- Measurement of weight (kg)
- Karnofsky Performance Status (KPS) (see Appendix B)
- Vital signs (see Section 5.2)
- Hematology: CBC with differential and platelet count
- Serum chemistries (see Section 5.2)
- CA 19-9 (or CA 125, or CEA if not expressers of CA 19-9)
- Urinalysis (see Section 5.2)
- Serum pregnancy if applicable
- Concomitant medication notation
- CT/MRI scan to evaluate disease status (using same imaging method as Baseline)
- AEs using the NCI CTCAE version 5.0 (see Appendix C)
- Patient to complete MDASI-GI and BPI questionnaires
- Confirm contact information for patient and a designated family member and remind patient of FU telephone contact that will be conducted monthly for survival status for up to 12 months from time of enrollment.

5.5 Follow-Up Assessments after End of Study or Early Termination

Follow-up assessments for survival, in person or by telephone, will be conducted for all patients 30 days after the End of Study or Early Termination Visit and then monthly for up to 12 months

from the time of enrollment. In the event that a patient cannot be reached during the follow-up period, a designated family member may be contacted.

6 EVALUATION OF RESPONSE

6.1 Best Overall Response

Best overall response is defined as the best response recorded from the start of the treatment until disease progression/recurrence (taking as reference for tumor progression, the smallest measurements recorded since the treatment started). The patient's best response assignment will depend on the achievement of both measurement and confirmation criteria.

6.2 Overall Tumor Response

The overall tumor response rate is defined as the total proportion of patients who have an objective tumor response (CR + PR). Rates for the individual categories of response (CR, PR, SD, and PD) will also be determined.

6.3 Not Evaluable

Patients will be defined as being not evaluable for response if a radiological assessment cannot be made. These patients will be counted as treatment failures in the analysis of tumor response data.

In some circumstances, it may be difficult to distinguish residual disease from normal tissue. When the evaluation of a CR depends upon this determination, it is recommended that the residual lesion be investigated by PET scan, fine needle aspirate or biopsy when possible before confirming the CR status.

6.4 Progression-free Survival

Progression-free survival (PFS) is defined as the time from enrollment (i.e. assignment of patient ID number) to first documentation of objective tumor progression or to death due to any cause in the absence of previous documentation of objective tumor progression will be censored at the last date the patient was known to be progression-free in patients who do not have objective tumor progression and who are: 1) still on study at the time of an analysis; 2) are given anti-tumor treatment other than the study treatment; or 3) are removed from study follow-up prior to documentation of objective tumor progression.

6.5 Survival

Survival is defined as the time from enrollment (i.e. assignment of patient ID number) to date of death. In the absence of confirmation of death, survival time will be censored at the last date the patient is known to be alive.

6.6 Guidelines for Measuring Disease

Antitumor activity will be evaluated by RECIST 1.1 criteria (Eisenhauer 2009). These response criteria are widely recognized and accepted as the standard criteria for determining response in patients with solid tumors.
https://ctep.cancer.gov/protocolDevelopment/docs/recist_guideline.pdf

6.7 Disease Definitions

Measurable disease is defined as the presence of ≥1 measurable lesion. If the measurable disease is restricted to a solitary lesion, its neoplastic nature should be confirmed by cytology/histology.

At Baseline, tumor lesions will be categorized as:

Measurable Lesions: lesions that can be accurately measured in at least one dimension (longest diameter in the plane of measurement is to be recorded) with a minimum size of:

- 10 mm by CT scan (CT scan slice thickness no greater than 5 mm)
- 10 mm caliper measurement by clinical exam (lesions which cannot be accurately measured with calipers should be recorded as non-measurable)
- 20 mm by chest X-ray.
- Malignant lymph nodes: to be considered pathologically enlarged and measureable, a lymph node must be ≥15 mm in short axis when assessed by CT scan (CT scan slice thickness recommended to be no greater than 5 mm). At Baseline and follow-up, only the short axis will be measured and followed.

Or

Non-measurable Lesions: all other lesions, including small lesions (longest diameter <10 mm or pathological lymph nodes with ≥10 to <15 mm short axis) as well as truly non-measurable lesions. Lesions considered truly non-measurable include: leptomeningeal disease, ascites, pleural/pericardial effusion, inflammatory breast disease, lymphangitis cutis/pulmonis, abdominal masses/abdominal organomegaly identified by physical exam that is not measurable by reproducible imaging techniques.

For special considerations regarding lesion measurability for bone lesions, cystic lesions and lesions with prior local treatment, consult the RECIST 1.1 guidelines.

All measurements should be taken and recorded in metric notation using a ruler or calipers. All baseline evaluations should be performed as closely as possible to the beginning of treatment and never more than 4 weeks before beginning of treatment.

The same method of assessment and the same technique should be used to characterize each identified and reported lesion at baseline and during follow-up.

Clinical lesions will only be considered measurable when they are superficial (e.g. skin nodules and palpable lymph nodes). For the case of skin lesions, either a CT scan or documentation by color photography, including a ruler to estimate the size of the lesion, is to be done.

6.8 Methods of Measurement

Tumor measurements should be performed using the same method as well as the same staff member per patient, if possible, throughout the study. CT and MRI are the best currently available and reproducible methods to measure target lesions selected for response assessment. Conventional CT and MRI should be performed with cuts of ≤5 mm in slice thickness contiguously. This applies to tumors of the chest, abdomen, and pelvis.

As a rule, the minimum size of the lesion should be no less than double the slice thickness.

Lesions on chest x-rays are acceptable as measurable lesions when they are clearly defined and surrounded by aerated lung. However, CT is preferable.

Ultrasound should not be used to measure tumor lesions. It is, however, a possible alternative to clinical measurements of superficial palpable lymph nodes, subcutaneous lesions, and thyroid nodules. Ultrasound might also be useful to confirm the complete disappearance of superficial lesions usually assessed by clinical examination. If new lesions are identified by ultrasound in the course of the study, confirmation by CT or MRI is necessary.

Tumor markers *alone* cannot be used to assess objective tumor response. If markers are initially above the upper normal limit, they must normalize for a patient to be considered in complete clinical response.

Cytology and histology can be used to differentiate between partial response (PR) and complete response (CR) in rare cases (e.g. after treatment to differentiate between residual benign lesions and residual malignant lesions in tumor types such as germ cell tumors) if required by protocol.

6.9 Baseline Documentation of “Target” and “Non-target” Lesions

Target Lesions: all measurable lesions up to a maximum of 2 lesions per organ and 5 lesions in total, representative of all involved organs will be identified as target lesions and will be recorded and measured at baseline. Target lesions should be selected on the basis of their size (lesions with the longest diameter [LD]), be representative of all involved organs, and their suitability for accurate repetitive measurements (either by imaging techniques or clinically). A sum of the LD for all target lesions will be calculated and reported as the baseline sum LD. The baseline sum of the LD will be used as reference to further characterize the objective tumor response.

Non-target Lesions: all other lesions (or sites of disease) including pathological lymph nodes should be identified as non-target lesions and will also be recorded at baseline. Measurements of these lesions are not required, but the presence or absence of each should be noted throughout follow-up.

6.10 Response Criteria

Table 6. RECIST 1.1 Target Lesion Response Criteria

Target Response Criteria	Definition
Complete Response (CR)	The disappearance of all target lesions and no new sites or disease-related symptoms confirmed at least 4 weeks after initial documentation. Any pathological lymph nodes (whether target or non-target) must have a reduction in short axis to < 10 mm. All sites must be assessed, including non-measurable sites, such as effusions, or markers.
Partial Response (PR)	At least a 30% decrease in the sum of the diameters of target lesions, taking as a reference the baseline sum of the diameters confirmed at least 4 weeks after initial documentation. PR is also recorded when all measurable disease has completely disappeared, but a non-measurable component (i.e., ascites) is still present but not progressing.
Stable Disease (SD)	Neither sufficient shrinkage to qualify for partial response nor sufficient increase to qualify for progressive disease.
Progressive Disease (PD)	At least a 20% increase in the sum of the diameters of target lesions, taking as reference the smallest sum of the diameters recorded since on study (this includes the baseline sum if that is the smallest on study), which must also demonstrate an absolute increase of at least 5 mm; or the appearance of one or more new lesions;

Non-target lesion response will be classified according to the RECIST Non-Target Lesion Response Criteria in the following table.

Table 7. RECIST 1.1 Non-target Lesion Response Criteria

Non-Target Response Criteria	Definition
Complete Response (CR)	Disappearance of all non-target lesions and normalization of tumor marker level confirmed at least 4 weeks after initial documentation. All lymph nodes must be non-pathological in size (< 10 mm short axis).
Non-Complete Response/Non- Progressive Disease (Non-CR/Non-PD)	Persistence of one or more non-target lesions and/or the maintenance of tumor marker level above the normal limits.
Progressive Disease (PD)	Appearance of one or more non-target lesions and/or unequivocal progression of existing non-target lesions ("unequivocal progression" is defined as an overall level of substantial worsening in non-target disease that is of magnitude that, even in the presence of SD or PR in target disease, the treating physician would feel it important to change therapy).

6.11 Evaluation of Best Overall Response

The best overall response is the best response recorded from the start of the treatment until disease progression/recurrence (taking as reference for PD the smallest measurements recorded since the treatment started). In general, the patient's best response assignment will depend on the achievement of both measurement and confirmation criteria.

Patients with a global deterioration of health status requiring discontinuation of treatment without objective evidence of disease progression at that time will be classified as having "symptomatic deterioration." Every effort will be made to document the objective evidence of disease progression even after discontinuation of treatment.

In some circumstances, it may be difficult to distinguish residual disease from normal tissue. When the evaluation of complete response depends on this determination, it is recommended that the residual lesion be investigated (fine needle aspirate/biopsy) to confirm the complete response status.

Table 8. RECIST Overall Response Criteria

Target Response	Non-Target Response	New Lesions	Overall Response
CR	CR	No	CR
CR	non-CR/non-PD	No	PR
CR	Not Evaluated	No	PR
PR	Non-PD or not evaluated	No	PR
SD	Non-PD or not evaluated	No	SD
Not all evaluated	Non-PD	No	NE
PD	Any	Yes or No	PD
Any	PD	Yes or No	PD
Any	Any	Yes	PD

NE = inevaluable

6.12 Duration of Overall Response

The duration of overall response is measured from the time measurement criteria are met for CR or PR (whichever status is recorded first) until the first date that recurrence or PD is objectively documented, taking as reference for PD the smallest measurements recorded since the treatment started.

6.13 Duration of Stable Disease

Stable disease is measured from the start of the treatment until the criteria for disease progression are met, taking as reference the smallest measurements recorded since the treatment started.

For the purposes of this trial, the minimal time interval required between 2 measurements for determination of SD is 6 weeks (at least 2 consecutive assessments 6 weeks apart revealing SD). This time interval takes into account the expected clinical benefit that such a status may bring to the population under study.

7 SAFETY

The investigator is responsible for monitoring the safety of patients who have enrolled in the study. All adverse events (AEs) occurring after any administration of the study medication will be followed until the event resolves, until the patient begins alternative treatment, or until the end of the study. Investigators will grade AEs using the NCI CTCAE, version 5.0 (see Appendix C).

Investigators are required to document all AEs regardless of causality, observed during the study period, starting with initial dose of study drug through the 30 days after patient's last dose of study drug, or until the beginning of a new anti-cancer therapy, whichever occurs first per section 7.9.

AEs occurring following the signature of the informed consent, but prior to the first dose of study drug, will not be reported as AEs. It is also important to record all AEs that result in permanent discontinuation of the investigational product being studied, whether serious or non-serious. Serious adverse events (SAEs), as defined below, must be reported to (Sponsor/Celgene) within 24 hours of knowledge of their occurrence.

7.1 Adverse Events

An AE is any unfavorable medical occurrence in a clinical investigation patient administered a pharmaceutical product, which does not necessarily have to have a causal relationship with this treatment. An AE can therefore be any unfavorable and unintended sign, symptom, or disease temporally associated with the use of a medicinal (investigational) product, whether or not considered related to the medicinal (investigational) product. All AEs, including observed or volunteered problems, complaints, or symptoms, are to be recorded on the CRF. Each AE is to be evaluated for duration, intensity, and causal relationship with the study medication or other factors.

Progression of disease is considered an efficacy outcome parameter and should not be captured as an AE.

A non-serious AE is any untoward medical occurrence that does not meet any of the criteria for SAEs.

Patients should be instructed to report any AE that they experience to the Investigator, starting from the time of their first dose. Investigators should assess AEs at each visit. AEs occurring during the clinical trial, starting at the time of the initial study drug infusion, and the follow-up period should be recorded on the appropriate AE page of the CRF.

Wherever possible, a specific disease or syndrome rather than individual associated signs and symptoms should be identified by the investigator and recorded on the CRF. However, if an observed or reported sign or symptom is not considered a component of a specific disease or syndrome by the investigator, it should be recorded as a separate AE on the CRF.

An abnormal laboratory value is considered to be an AE if the abnormality:

- results in discontinuation from the study;
- requires treatment, modification/ interruption of IP dose, or any other therapeutic intervention;
- or is judged to be of significant clinical importance, e.g., one that indicates a new disease process and/or organ toxicity, or is an exacerbation or worsening of an existing condition.

7.2 Serious Adverse Events

A serious adverse event (SAE) is any adverse experience occurring at any dose that results in any of the following outcomes:

- Death
- A life-threatening adverse event
- Life-threatening is defined as an event with immediate risk of death from the event as it occurred. It does not include an event that might have caused death if it occurred with a greater severity.
- In-patient hospitalization or prolongation of existing hospitalization, except for the following:
    - A standard procedure for protocol therapy administration. However, hospitalization or prolonged hospitalization for a complication of therapy administration will be reported as an SAE.
    - Routine treatment or monitoring of the studied indication not associated with any deterioration in condition.
    - The administration of blood or platelet transfusion as routine treatment of studied Indication. However, hospitalization or prolonged hospitalization for a complication of such transfusion remains a reportable SAE.
    - A procedure for protocol/disease-related investigations (e.g., surgery, scans, endoscopy, sampling for laboratory tests, bone marrow sampling). However, hospitalization or prolonged hospitalization for a complication of such procedures remains a reportable SAE.
    - Hospitalization or prolongation of hospitalization for technical, practical, or social reasons, in absence of an AE.
    - A procedure that is planned (i.e., planned prior to starting of treatment on study); must be documented in the source document and the CRF. Hospitalization or prolonged hospitalization for a complication remains a reportable SAE.
    - An elective treatment of or an elective procedure for a pre-existing condition unrelated to the studied indication.

Emergency outpatient treatment or observation that does not result in admission, unless fulfilling other seriousness criteria above.

- Results in a persistent or significant disability/incapacity
- A congenital anomaly/birth defect
- Important medical events that may not be immediately life-threatening, or result in death or hospitalization, but may jeopardize the patient or may require intervention to prevent one of the other listed outcomes above.

7.3 Adverse Event Severity

AE will be graded according to the NCI CTCAE, Version 5.0 (see Appendix C)

- Grade 1 Mild AE
- Grade 2 Moderate AE
- Grade 3 Severe AE
- Grade 4 Life-threatening or disabling AE
- Grade 5 Death related to AE

Severity versus Seriousness: Severity is used to describe the intensity of a specific event while the event itself, however, may be of relatively minor medical significance (such as severe headache). This is not the same as "seriousness," which is based on patient/event outcome at the time of the event. For example, the NCI CTCAE grade 4 (life-threatening or disabling AE) is assessed based on unique clinical descriptions of severity for each AE, and these criteria may be different from those used for the assessment of AE seriousness. An AE assessed as grade 4 based on the NCI CTCAE grades may or may not be assessed as serious based on the seriousness criteria.

7.4 Duration

For both AEs and SAEs, the Investigator will provide a record of the start and stop dates of the event.

7.5 Action Taken

The Investigator will report the action taken for each trial treatment (including nab-paclitaxel, cisplatin, and gemcitabine) as a result of an AE or SAE, as applicable (e.g., discontinuation, interruption, or dose reduction of specified study treatment, as appropriate) and report if concomitant and/or additional treatments were given for the event.

7.6 Outcome

The Investigator will report the outcome of the event for both AEs and SAEs. All SAEs that have not resolved upon discontinuation of the subject's participation in the study must be followed until recovered (returned to baseline), recovered with sequelae, not recovered or death (due to the SAE).

7.7 Causality Assessment

All adverse events regardless of CTCAE grade must also be evaluated for relationship (in regards to each study treatment including/ trial interventions). This is to be recorded for each study treatment including nab-paclitaxel, cisplatin, and gemcitabine.

The determination of the likelihood that trial treatment caused the adverse event will be provided by an investigator. The Investigator must determine the relationship between the administration of study treatment and the occurrence of an AE/SAE as Not Suspected or Suspected as defined below:

Relationship	Attribution
Not Suspected	Not Related
	Unlikely
Suspected	Possibly
	Probably
	Definite

Not suspected: Means a causal relationship of the adverse event to IP administration is unlikely or remote, or other medications, therapeutic interventions, or underlying conditions provide a sufficient explanation for the observed event.

Suspected: Means there is a reasonable possibility that the administration of IP caused the adverse event. ‘Reasonable possibility’ means there is evidence to suggest a causal relationship between the IP and the adverse event.

Causality should be assessed and provided for every AE/SAE based on currently available information. Causality is to be reassessed and provided as additional information becomes available.

The Medical Monitor will make the final determination of relationship between the administration of study treatment and the occurence of the reported AEs/SAEs.

7.8 Patient Reporting of AEs and SAEs

Patients are to be encouraged to call the site to report any unexpected symptoms or problems they encounter between office visits. These events should be considered in the same fashion as if they had been reported at a scheduled office visit. At each scheduled office visit, after the patient has had an opportunity to spontaneously mention any problems, the Investigator should inquire about AEs by asking the following standard questions

- Have you had any (other) medical problems since your last clinic visit?
- Have you taken any new prescribed or over-the-counter medicines or herbal/vitamin preparations, other than those given to you in this study, since your last visit/assessment?
- Have any new procedures been performed since your last study visit?

7.9 Investigator Reporting Adverse Events

The Investigator is responsible for recording and following all Grade ≥3 AEs, all grade neuropathy adverse events, any AE that leads to study treatment discontinuation and any AE that is associated with and contributing to an SAE as determined by the Investigator regardless of causality, observed during the study period, starting with initial dose of study drug through the 30 days after patient’s last dose of study drug, or until the beginning of a new anti-cancer therapy, whichever occurs first. The Investigator should follow AEs until the event is resolved or stabilized, the patient is lost to follow-up, or the event is otherwise explained. Events occurring within 30 days prior to study drug administration should be recorded as pre-treatment signs and symptoms. The only exception to this is for an AE that occurs prior to the first dose of study drug but is due to a procedure associated with assessments carried out to determine eligibility or to permit participation in this protocol – this should be recorded as an AE (rather than a pre-treatment sign or symptom).

The Investigator or designee must completely and promptly record each AE in the source documentation, regardless of relationship to study drug as determined by the Investigator.

The Investigator must assess AE/SAE causality for any patients treated at his/her site. The Investigator should attempt, if possible, to establish a diagnosis based on the patient's signs and symptoms. When a diagnosis for the reported signs or symptoms is known, the Investigator should report the diagnosis, not the symptoms, as the AE.

Clinically significant laboratory abnormalities present at the baseline visit will be recorded as pre-treatment signs and symptoms.

For the purposes of this study, only the following adverse events are to be recorded in the eCRFs:

- Grade ≥ 3 AEs
- All grade neuropathy adverse events
- Any AE that leads to study treatment discontinuation
- Any AE that is associated with and contributing to an SAE as determined by the Investigator

In addition, all SAEs must be reported promptly to both Triligent and Celgene after the Investigator recognizes/classifies the event as a SAE. The specific reporting time frame depends on the type of SAE. For life-threatening or fatal events, the Investigator must report initial information on the SAE within 24 hours/ 1 business day of becoming aware of the event, preferably by email or alternatively by phone or fax; at a minimum, a description of the event and the Investigator's judgment of causality must be provided at the time of the initial report. If an SAE is reported by phone the Investigator must email or fax a completed SAE report form along with the SAE Report Form Cover letter to (Triligent and Celgene) within 1 business days. For an event that is not life-threatening or fatal, the Investigator must email a completed SAE report form within 2 business days after he/she recognizes/classifies the event as an SAE.

The Investigator should follow all AEs/SAEs observed during the study until the AE has subsided, or until the condition stabilizes (in the case of persistent impairment), the patient receives alternative therapy, or the patient dies.

For any additional questions regarding reporting requirements of a SAE, please contact the Triligent Project Lead as listed in the study contact list.

7.9.1 Expedited Reporting by Investigator to (Sponsor/Celgene)

Serious adverse events (SAE) are defined above in Section 7.2.

The investigator must inform both the Sponsor (via CRO, Triligent) and Celgene in writing using a MEDWATCH 3500A form of any SAE within 24 hours of being aware of the event. The Medwatch 3500A form along with the SAE Report Form Cover letter must be completed and supplied to Triligent and Celgene by email within 24 hours. The initial report must be as complete as possible, including an assessment of the causal relationship between the event and each trial treatment (including nab-paclitaxel, cisplatin, and gemcitabine). Information not available at the time of the initial report (e.g., an end date for the adverse event or laboratory values received after the report) must be documented on a follow-up MEDWATCH 3500A form. A final report to document resolution of the SAE is required. The Celgene tracking number AX-CL-PANC-PI-13301 and the institutional protocol number should be included on SAE reports (or on the cover letter) sent to Triligent and Celgene. A copy of email or the fax transmission confirmation of the

SAE report to Triligent and Celgene should be attached to the SAE and retained with the patient records.

Note: Participating study sites should NOT report SAEs to the FDA. Celgene will be responsible for reporting to FDA.

SAE Reporting to the Sponsor (via CRO, Triligent):

Email: SAE@Triligent.com
Fax: 949-203-6151
Telephone: 317-402-9777

Drug Safety Contact Information:
Celgene Corporation
Global Drug Safety and Risk Management
86 Morris Avenue
Summit, New Jersey 07901
Fax: (908) 673-9115
E-mail: drugsafety@celgene.com
Telephone: 1-908-673-9667
Toll Free: 1-800-640-7854

7.9.2 IRB Notification of SAEs

The Investigator is responsible for promptly notifying the IRB of all SAEs, including any follow-up information per local IRB guidelines, occurring at his/her site and any SAE regulatory reports and Investigational New Drug Safety Reports that he/she receives from (Sponsor/Celgene).

7.10 SAE Follow-Up

For all SAEs occurring after first dose of study medication or within 30 days of the last administration of study medication, the investigator must submit follow-up reports to (Sponsor/Celgene) regarding the status of the SAE and the patient’s subsequent course until the SAE has subsided, or until the condition stabilizes (in the case of persistent impairment), the patient receives alternative therapy, or the patient dies.

7.10.1 Sponsor Notification of Post-Study SAEs

The Investigator should notify (Sponsor/Celgene) of any death or SAE occurring after a patient has withdrawn from the study, or after 30 days of the last study drug dose, whichever is later, when such death or SAE may reasonably be related to the medication used in the study. However, Investigators are not obligated to actively seek AEs in former study participants.

7.11 Pregnancy

While not considered a SAE unless a serious criterion is met, pregnancies occurring in patients enrolled on the study or in their partners must be reported. The investigator should complete the pregnancy report form and fax it to (Sponsor/CRO) within 24 hours of knowledge of the pregnancy.

Pregnancies and suspected pregnancies (including a positive pregnancy test regardless of age or disease state) of a female subject occurring while the subject is on study treatment, or within 30 days of the subject’s last dose study treatment, are considered immediately reportable events.

Study treatment is to be discontinued immediately. The pregnancy, suspected pregnancy, or positive pregnancy test must be reported to HRI and Celgene Drug Safety immediately by facsimile, or other appropriate method, using the Pregnancy Initial Report Form, or approved equivalent form. The female subject may be referred to an obstetrician-gynecologist (not necessarily one with reproductive toxicity experience) or another appropriate healthcare professional for further evaluation.

The Investigator will follow the female subject until completion of the pregnancy, and must notify Celgene Drug Safety immediately about the outcome of the pregnancy (either normal or abnormal outcome) using the Pregnancy Follow-up Report Form, or approved equivalent form.

If the outcome of the pregnancy was abnormal (e.g., spontaneous or therapeutic abortion), the Investigator should report the abnormal outcome as an AE. If the abnormal outcome meets any of the serious criteria, it must be reported as an SAE to Celgene Drug Safety immediately by facsimile, or other appropriate method, within 24 hours of the Investigator's knowledge of the event using the SAE Report Form, or approved equivalent form.

All neonatal deaths that occur within 28 days of birth should be reported, without regard to causality, as SAEs. In addition, any infant death after 28 days that the Investigator suspects is related to the in utero exposure to the IP should also be reported to Celgene Drug Safety immediately by facsimile, or other appropriate method, within 24 hours of the Investigator's knowledge of the event using the SAE Report Form, or approved equivalent form.

Male Subjects

If a female partner of a male subject taking investigational product becomes pregnant, the male subject taking IP should notify the Investigator, and the pregnant female partner should be advised to call their healthcare provider immediately. Male patients treated with nab-paclitaxel are advised not to father a child during and up to 6 months after treatment.

7.12 Overdose

Overdose, as defined for this protocol, refers to nab-Paclitaxel, cisplatin and gemcitabine dosing.

On a per dose basis, an overdose is defined as the following amount over the protocol-specified dose of nab-paclitaxel, cisplatin or gemcitabine assigned to a given patient, regardless of any associated adverse events or sequelae.

IV 10% over the protocol-specified dose

On a schedule or frequency basis, an overdose is defined as anything more frequent than the protocol required schedule or frequency.

On an infusion rate basis, an overdose is defined as any rate faster than the protocol-specified rate. For nab-paclitaxel, an infusion completed in less than 25 minutes may increase Cmax by approximately 20%, therefore a nab-paclitaxel infusion completed in less than 25 minutes will meet the infusion rate criterion for an overdose.

Complete data about drug administration, including any overdose, regardless of whether the overdose was accidental or intentional, should be reported in the case report form.

Any sequela of an accidental or intentional overdose of study treatment should be reported as an AE. If the sequela of an overdose is an SAE, then the sequela must be reported on a Medwatch

3500A form and as an AE. The overdose resulting in the SAE should be identified as the cause of the event on the Medwatch 3500A form and CRF but should not be reported as an SAE itself.

In the event of overdose, the subject should be monitored as appropriate and should receive supportive measures as necessary. There is no known specific antidote for nab-paclitaxel, cisplatin or gemcitabine overdose. Actual treatment should depend on the severity of the clinical situation and the judgment and experience of the treating physician.

Drug Safety Contact Information:
Celgene Corporation
Global Drug Safety and Risk Management
86 Morris Avenue
Summit, New Jersey 07901
Fax: (908) 673-9115
E-mail: drugsafety@celgene.com
Telephone: 1-908-673-9667
Toll Free: 1-800-640-7854

8 STATISTICAL CONSIDERATIONS

8.1 Trial Design

This is a phase II open-label trial, with the identity of the treatment known to the patients, Investigators, Triligent and Sponsor.

8.2 Sample Size Considerations

The a-priori alpha level for all statistical tests will be set at .025 (one-sided). Sample size is based on the phase III IMPACT trial (Von Hoff, 2013), demonstrating a 12-month OS of 35%, versus 12-month OS of 67.5% in the PCRT 12-001 trial (Jameson, 2017). The historic control rate of 55% was selected to provide a conservative estimate of survival for the current study. To detect a difference between 35% and 55% 12-month OS rates with 80% power, hazard ratio of 0.57, and a one-sided 0.025 alpha-level, 42 patients are needed. To account for attrition, a total of 50 patients will be enrolled in the investigation.

A log--rank test will be used to test the null hypothesis that there is no difference between the conditions in the probability of an event:
H_0: $\delta \leq 1$ vs. H_1:$\delta > 1$

8.3 Statistical Analysis Plan

8.3.1 Analysis of the Conduct of the Trial

Enrollment, major protocol deviations, and discontinuations for the trial will be summarized using a CONSORT diagram.

8.3.2 Patient Disposition

A detailed description of patient disposition will include:

- A summary of data on patient discontinuation from treatment.
- A summary of data on overall qualification status of all patients.

- An account of all identified protocol deviations.

All patients enrolled in the study will be included in the summation. An evaluable patient is any patient who has received even one dose of any of the study medications. The number of patients who do not qualify for analysis, who die or discontinue before treatment begins, will be specified.

8.3.3 Analysis of Patient Characteristics

Patient characteristics will include a summary of the following:

- Patient demographics
- Baseline disease characteristics
- Pre-existing conditions
- Prior therapies
- Concomitant medications and treatments

Other patient characteristics will be summarized as appropriate.

8.4 Efficacy Analysis

The efficacy analysis will include the following parameters: objective response rate (CR + PR), disease control rate (CR + PR + SD at 9 weeks), progression-free survival (PFS), stable disease rate at 9 weeks (SD), and overall survival (OS). The efficacy analysis will only be conducted on patients who have received at least one dose of cisplatin, nab-paclitaxel and gemcitabine and have at least one post baseline tumor assessment.

Objective responses will be evaluated using the Response Evaluation Criteria In Solid Tumors 1.1 (RECIST 1.1). Changes (i.e. improvements) in tumor measurements from baseline values will be assigned a status of CR or PR or SD. Objective response measurements will comprise the sum of CR plus PR. The overall response rate, as well as the rates for the individual categories of response (i.e. CR, PR, SD, and PD), will be estimated by the percentage of patients achieving these criteria. The disease control rate will consist of the sum of CR + PR + SD for 9 weeks.

Progression-free survival is defined as the interval from the date of registration (i.e. assignment of patient number) to the earliest date of documented evidence of recurrent or progressive disease, or the date of death due to any cause, whichever occurs first. For the estimation of progression-free and overall survival a Kaplan-Meier analysis will be performed.

Overall survival will be measured from the date of registration (i.e. assignment of patient number) to the date of death due to any cause, or the date of last contact (censored observations).

Time-to-event endpoints, including PFS and OS will be assessed using the Kaplan-Meier method (Kaplan 1958).

Objective response rates, clinical benefit response, and CA 19-9, will be summarized descriptively.

Continuous variables will be summarized using the mean (SD) or median (range). Frequency tables will be used to summarize categorical variables. Logistic regression will be used to assess the impact of patient characteristics on response and toxicity rates. The distribution of time-to-event endpoints (e.g. response duration, progression-free survival, overall survival) will be estimated using the Kaplan and Meier method. Cox (proportional hazards) regression will be used to evaluate multivariable predictive models of time-to-event outcomes.

8.5 Safety and Tolerance Analysis

All patients who receive any amount of cisplatin, nab-paclitaxel and gemcitabine will be included in the safety analyses.

The incidence of all AEs (regardless of causality) and all treatment-related AEs (those AEs thought to be suspected related to study drug) will be summarized by NCI CTCAE version 5.0 term and maximum grade. The incidence of SAEs and AEs that lead to discontinuation of study drug will also be summarized. Listings of patients who discontinue study drug due to an AE and patients with SAEs and deaths will be presented. Narratives will be provided for patients who experience an SAE.

8.5.1 Stopping Rules

The study will be stopped early based on either one of the following criteria being met:

1. Treatment related deaths > 2 patients in the first 25 patients, as that is the number we observed in the original 25 patient study.

2. Treatment related bleeding in the first 25 patients that is life threatening and/or fatal in > 1 patient

8.6 Laboratory Assessments

8.6.1 Hematology Parameters

To investigate the maximal degree of myelosuppression, the NCI CTCAE version 5.0 grade for WBC, ANC, platelet count, and hemoglobin concentration will be summarized by the most severe grade in the first treatment cycle and by the most severe grade during the therapy for each treatment group; testing of treatment group differences will be performed using a CMH test. The incidence of patients with NCI CTCAE version 5.0 hematology values of Grade 3 or 4 that occurred after the first dose of study drug will be presented. Data for patients with Grade 3 or 4 hematology values will be listed.

8.6.2 Clinical Chemistry

Liver and renal function will be summarized using the NCI CTCAE version 5.0 for alkaline phosphatase, ALT, AST, total bilirubin, BUN and creatinine. The number and percentage of patients who have more than one NCI CTCAE version 5.0 grade will be summarized using the most severe Grade for the first cycle of therapy and for anytime during therapy for each treatment group; testing of treatment group differences will be performed using a Cochran-Mantel-Hanzel test. The incidence of patients with NCI CTCAE version 5.0 chemistry values of Grade 3 or 4 that occurred after the first dose of study drug will be presented. Data for patients with Grade 3 or 4 chemistry values will be listed.

8.6.3 Peripheral Neuropathy

Peripheral neuropathy events will be captured according to protocol and reported by Investigators and investigative staff in accordance with adverse event and SAE reporting standards.

8.7 Replacement of Patients

Patients who are enrolled into the study, but fail to receive nab-paclitaxel, cisplatin, and gemcitabine may be replaced.

9 ETHICAL, LEGAL, AND ADMINISTRATIVE ASPECTS

9.1 Ethics

9.1.1 Institutional Review Board/Ethics Committee Approval

Before study initiation, this protocol and informed consent form will be submitted for review and approval to the IRBs charged with oversight for the clinical sites. In addition, any form of proposed advertising and advertising text for patient recruitment must be reviewed and approved by (Sponsor/CRO) prior to submission to the IRB. The Investigator will forward to (CRO or Sponsor) -nominated designee a copy of the IRB's approval of this protocol, any amendments, informed consent form, and any modifications to the informed consent, based on the FDA regulations set forth in 21 CFR 56 of the *Code of Federal Regulations,* as well as those of the applicable regulatory bodies in all other participating countries outside of the U.S.

In addition, the Investigator will be responsible for forwarding to (CRO or Sponsor) -nominated designee a description of the IRB board members (including profession and affiliation) or a United States (US) Department of Health and Human Services (DHHS) General Assurance number and expiration date. If neither of these is available, the chairperson must submit a statement indicating that the members of the board responsible for the review meet FDA and other appropriate regulatory requirements. In addition, the labeling for all approved study drugs should be submitted to the IRB for informational purposes.

9.1.2 Ethical Conduct of the Study

This study will be conducted in compliance with the protocol, Good Clinical Practice (GCP), Guidelines of the International Conference on Harmonization (ICH) of Technical Requirements for Registration of Pharmaceuticals for Human Use, and in full compliance with the World Medical Association Declaration of Helsinki and its most recent amendments.

9.1.3 Informed Consent

Written informed consent of the patient to participate in the study must be obtained and documented by the Investigator in accordance with the FDA regulations set forth in 21 CFR 50 as well as the applicable regulatory bodies in all other participating countries outside the United States.

The Investigator must provide the patient with a copy of the informed consent form in a language understandable to the patient. Written consent should be obtained before any protocol-required procedures are performed, including any procedure not part of normal patient care (e.g., withdrawal of current medications).

Changes made by a participating site to the recommended informed consent must be forwarded to HRI/Triligent for approval prior to submission to the corresponding IRB. A copy of the signed informed consent will be given to the patient or their legal representative and a copy must be retained in the Investigator's study records.

9.2 Disclosure of Data

9.2.1 Confidentiality

The Investigator and any other study personnel involved in this study shall not disclose, or use for any purposes (other than for the performance of this study), any data, records, or other information (hereinafter collectively "information") disclosed to the Investigator or other study personnel. Such information shall remain the confidential and proprietary property of HRI and shall be disclosed only to the Investigator or other designated study personnel.

The obligation of non-disclosure shall not apply to the following:

- relevant disclosure to potential study participants for the purpose of obtaining informed consent;
- information after such time that it is or becomes publicly available through no fault of the Investigator or other study personnel; and,
- information after such time that it is disclosed to the Investigator by a third party entitled to disclose such information.
- If the study site is a 'covered site' under the definitions of the Health Insurance Portability and Accounting Act (HIPAA), the Investigator will ensure that the patient consents to the use of data by HRI/ Triligent/Celgene and its designees for the purposes of regulatory submissions, study publications, and drug approval.

9.2.2 Publication

The Investigator(s) is obligated, consistent with academic standards and with due regard to the protection of Celgene or Triligent confidential information and intellectual property, to publish or present the results of work performed in accordance with the study; provided that any proposed publication or presentation is first reviewed and approved in writing by Celgene. Celgene shall complete its review within 30 days after receipt of the proposed publication or presentation. Upon Celgene request, proposed publication or presentation will be delayed up to 60 additional days to enable Celgene to secure adequate intellectual property protection of property of Celgene that would be affected by such proposed publication or presentation. If Celgene believes in good faith that any proposed publication or presentation contains any confidential information and/or intellectual property Celgene shall have the right to require HRI to remove the information. HRI agrees and will consider all other comments of Celgene in good faith.

9.2.3 Trial Registration and Results Posting Requirements

Under the terms of the Food and Drug Administration Modernization Act (FDAMA) and the Food and Drug Administration Amendments Act (FDAAA), the Sponsor of the trial is solely responsible for determining whether the trial and its results are patient to the requirements for submission to the Clinical Trials Data Bank, http://www.clinicaltrials.gov. Information posted will allow patients to identify potentially appropriate trials for their disease conditions and pursue participation by

calling a central contact number for further information on appropriate trial locations and trial site contact information.

9.3 Quality Assurance

9.3.1 Study Data Monitoring Plan

Data monitoring procedures will be carried out by Triligent International for all participating sites and will be performed on a regular basis to comply with Good Clinical Practice guidelines.

Review of the case report forms, cross-reference with source documentation (including radiology review), review of study related regulatory documents and logs (e.g., enrollment, study site staff training and delegation) and review of drug infusion records will be monitored on an ongoing basis during monitoring sessions. The monitor will ensure that the investigation is conducted according to protocol design and regulatory requirements.

At the conclusion of the monitoring visit, the site monitor will meet with the site staff to discuss and request specific corrections to the case report forms, and/or request clarification, and/or additional source documentation. The Principal Investigator and Clinical Research Coordinator responsible for the study will be provided with a follow-up letter for resolution of the findings.

The CRO site monitor will complete a written monitoring report and forward it to the HRI. The report will include a summary of what the site monitor reviewed and the site monitor's statements concerning the significant findings/facts, deviations and deficiencies, conclusions, actions taken or to be taken, and/or actions recommended to ensure compliance. The site Principal Investigator will be expected to submit any Corrective Action Plans, in writing, to HRI/Triligent Administration and the Triligent site monitor. A copy of the monitoring forms, final monitoring reports, and Corrective Action Plan will be kept in the site monitor's study file at Triligent International for follow-up at the next monitoring session.

9.3.2 Data Safety and Monitoring

This treatment regimen combines 3 chemotherapeutic agents with known toxicity profiles. Because cancer is a life-threatening disease, treatments that result in Grade 3 and 4 toxicities are considered to have an acceptable risk profile. Data reported to the sponsor will be received by the Lead Principal Investigator on a regular basis and not less than once a month. In addition, SAEs will be reported to the Sponsor immediately and reviewed as they are received. Any unacceptable toxicities or severe toxicities that occur more frequently than expected will be discussed by the Lead Principal Investigator and the site Principal Investigators who will decide jointly whether the study should be modified, interrupted, or stopped. A monthly conference call will be held with investigators participating in the study. The Sponsor will provide listings of toxicities on a regular basis.

9.3.3 On-site Audits

Regulatory authorities, the IEC/IRB, HRI and/or Triligent may request access to all source documents, data capture records, and other study documentation for on-site audit or inspection. Direct access to these documents must be guaranteed by the investigator, who must provide support at all times for these activities.

9.4 Premature Closure of the Study

This study may be prematurely terminated, if in the opinion of the Lead Principal Investigator or HRI there is sufficient reasonable cause.

HRI has the right to discontinue the study under the conditions specified in the clinical trials agreement. Written notification documenting the reason for study termination will be provided to the site investigator by the terminating party.

Circumstances that may warrant termination include, but are not limited to:

- Determination of unexpected, significant, or unacceptable risk to patients
- Failure to enter patients at an acceptable rate
- Insufficient adherence to protocol requirements
- Insufficient complete and/or evaluable data
- Plans to modify, suspend or discontinue the development of the drug
- Should the study be closed prematurely, all study materials must be returned to HRI.

9.5 Investigator Documentation

9.5.1 Form FDA 1572

The Investigator must provide HRI with a fully executed Form FDA 1572. Any additions to the study must be provided via a new fully executed Form FDA 1572. Others should follow FDA guidance "Information Sheet Guidance for Sponsors, Clinical Investigators, and IRBs".

9.5.2 Curriculum Vitae

The Investigator must provide HRI with his/her current signed and dated curriculum vitae and a current signed and dated curriculum vitae for each sub-Investigator listed on Form FDA 1572. Current signed and dated curriculum vitae is defined as updated within 2 years of study start up.

9.5.3 Investigator Protocol Agreement

The Investigator must sign the Investigator's Protocol Agreement. The original must be kept on file at the Investigator site and a copy provided to HRI/Triligent. The completed Investigator's Protocol Agreement signifies agreement to comply with all procedures outlined by this protocol by the Investigator. An Investigator's Protocol Agreement must be signed if and when a protocol amendment is issued by (Sponsor/CRO).

9.5.4 Financial Disclosures

The Investigator and sub-Investigator(s) must complete a Clinical Investigator Financial Certification/Disclosure Statement to report financial interests and arrangements that may be of concern to FDA per 21 CFR 54.

9.5.5 Laboratory Certification and Normal Ranges

The Investigator will indicate on the Form FDA 1572 the name and location of any local laboratories that will be used for laboratory assessments. The Investigator will provide a copy of all clinical laboratory certifications, certification numbers, and dates of certifications, and. Normal ranges for all laboratory tests will be included with all reported results. Updated versions of these documents must be provided to HRI/Triligent as appropriate. In the event the clinical laboratory

is changed during the study HRI /Triligent will be promptly notified, and the Form FDA 1572 will be updated. Appropriate documentation will be submitted to HRI/Triligent to verify the certification of the new laboratory.

All radiology facilities being utilized outside the investigative site must be pre-approved by HRI.

9.5.6 Records Retention

In accordance with applicable regulatory requirements, following closure of the study, the Investigator will maintain a copy of all site study records in a safe and secure location. HRI will inform the Investigator of the time period for retaining these records in order to comply with applicable regulatory requirements.

HRI reserves the right to terminate the study for refusal of the Investigator and/or investigational site to comply with any requirements stated in this study protocol.

10 DATA MANAGEMENT

10.1 Case Report Forms

All the clinical data will be captured by the site on electronic case report forms (eCRFs). The eCRFs will be used for all consented patients. The investigator and trained trial personnel will enter and edit the data via a secure network, with secure identification and password requirement. A complete electronic audit trial will be maintained. The investigator will be required to provide approval of all data to confirm accuracy. Copies of the eCRFs will be provided to the investigator at the conclusion of the trial.

Triligent International will monitor the study to verify study data, medical records, and eCRFs in accordance with current ICH GCP guidelines as well as other applicable regulations and guidelines.

10.2 Source Documents

Source documents serve as the evidence of the existence of the patient and the data collected for this trial. Source documents will be the responsibility of the Investigator and will be filed at the site and available as needed by the Sponsor, Triligent, or assigned clinical monitor.

Data captured on the eCRFs is to be transcribed from source document and must be consistent with any discrepancies explained and documented.

REFERENCES

1. American Cancer Society. Cancer Facts & Figures 2018. Atlanta: American Cancer Society. 2018; p 20-21. www.cancer.org

2. Alexandrov LB, Nik-Zainal S, Wedge DC, Aparicio SA, Behjati S, Biankin AV, Bignell GR, Bolli N, Borg A, Børresen-Dale AL, Boyault S. Signatures of mutational processes in human cancer. Nature. 2013 Aug;500(7463):415.

3. Alexandrov LB, Nik-Zainal S, Siu HC, Leung SY, Stratton MR. A mutational signature in gastric cancer suggests therapeutic strategies. Nature communications. 2015 Oct 29;6:8683.ssaf

4. E, Verlinde-Carvalho M, Delbaldo C, Grenier J, Sellam Z, Pouessel D, Bouaita L, Baumgaertner I, Sobhani I, Tayar C, Paul M, Culine S. 5-Fluorouracil/Leucovorin Combined with Irinotecan and Oxaliplatin (FOLFIRINOX) as Second-Line Chemotherapy in Patients with Metastatic Pancreatic Adenocarcinoma. Oncology 2011;80:301-306 (DOI: 10.1159/000329803)

5. Atkinson, T.M., Mendoza, T.R., Sit, L., Passik, S., Scher, H.I., Cleeland, C.S., & Basch, E. (2010), *Pain Medicine 11*, 337-346.

6. Barrett MT, Deiotte R, Lenkiewicz E, Malasi S, Holley T, Evers L, Posner RG, Jones T, Han H, Sausen M, , Velculescu VE, Jameson G, Von Hoff DD. Clinical study of genomic drivers in pancreatic ductal adenocarcinoma. British Journal of Cancer. 2017 Aug;117(4):572.

7. Barrett, M.T., et al., The association of genomic lesions and PD-1/PD-L1 expression in resected triple-negative breast cancers. Breast Cancer Res, 2018. 20(1): p. 71.

8. Burris HA 3rd, Moore MJ, Andersen J, Green MR, Rothenberg ML, Modiano MR, Cripps MC, Portenoy RK, Storniolo AM, Tarassoff P, Nelson R, Dorr FA, Stephens CD, Von Hoff DD. Improvements in survival and clinical benefit with gemcitabine as first-line therapy for patients with advanced pancreas cancer: a randomized trial. J Clin Oncol. 1997 Jun; 15(6):2403-13.

9. Cisplatin Prescribing Information (updated 2012) – Accessed via Daily Med (National Library of Medicine) http://dailymed.nlm.nih.gov/dailymed/lookup.cfm?setid=a440f077-46f6-4688-a209-65bce38d1c92

10. Cleeland CS, Mendoza TR, Wang XS, Chou C, Harle M, Morrissey M, & Engstrom MC. Assessing Symptom Distress in Cancer: The M. D. Anderson Symptom Inventory. Cancer 2000; 89:1634-1646.

11. Conroy T, Desseigne F, Ychou M, Bouché O, Guimbaud R, Bécouarn Y, Adenis A, Raoul J-L, Gourgou-Bourgade S, de la Fouchardière C, Bennouna J, Bachet J-B, Khemissa-Akouz F, Péré-Vergé D, Delbaldo C, Assenat E, Chauffert B, Michel P, Montoto-Grillot C, Ducreux M. FOLFIRINOX versus gemcitabine for metastatic pancreatic cancer. N Engl J Med. 2011;364:1817-1825.

12. Dragovich T: Pancreatic Cancer. eMedicine.Medscape.Com, 2012. Available from: http://emedicine.medscape.com/article/280605.

13. Eisenhauer EA, Therasse P, Bogaerts J, Schwartz LH, Sargent D, Ford R, Dancey J, Arbuck S, Gwyther S, Mooney M, Rubinstein L, Shankar L, Dodd L, Kaplan R, Lacombe D, Verweij J. New response evaluation criteria in solid tumours: revised RECIST guideline (version 1.1). Eur J Cancer. 2009 Jan; 45(2):228-47.

14. Frasci G, Panza N, Comella P, Nicolella GP, Natale M, Manzione L, Bilancia D, Cioffi R, Maiorino L, De Cataldis G, Belli M, Micillo E, Mascia V, Massidda B, Lorusso V, De Lena M, Carpagnano F, Contu A, Pusceddu G, Comella G. Cispatin, gemcitabine, and paclitaxel in locally advanced or metastatic non-small-cell lung cancer: A phase I-II study. J Clin Oncology. Journal of Clinical Oncology. 1999 Aug;17(8):2316-2325.

15. Frese KK, Neesse A, Cook N, Bapiro TE, Lolkema MP, Jodrell DI, Tuveson DA. Nab-paclitaxel potentiates gemcitabine activity by reducing cytidine deaminase levels in a mouse model of pancreatic cancer. Cancer Discovery. 2012;2(3):OF1-OF10. DOI: 10.1158/2159-8290.CD-11-0242

16. Gemcitabine (Gemzar™) Prescribing Information (Updated 2/2011) – Accessed via Gemzar Website: http://pi.lilly.com/us/gemzar.pdf

17. Heinemann V, Boeck S, Hinke A, Labianca R, Louvet C. Meta-analysis of randomized trials: evaluation of benefit from gemcitabine-based combination chemotherapy applied in advance pancreatic cancer. BMC Cancer. 2008;28:82. DOI: 10.1186/1471-2407-8-82.

18. Holley, T., et al., Deep clonal profiling of formalin fixed paraffin embedded clinical samples. PLoS One, 2012. 7(11): p. e50586.

19. Jameson GS, Borazanci E, Babiker HM, Poplin E, Niewiarowska AA, Gordon MS, Barrett MT, Rosenthal A, Stoll-D'Astice A, Crowley J, Shemanski L, Korn RL, Ansaldo K, Lebron L, Ramanathan RK, Von Hoff DD. 2019. Response rate following albumin-bound paclitaxel plus gemcitabine plus cisplatin treatment among patients with advanced pancreatic cancer: a phase 1b/2 pilot clinical trial. *JAMA oncology*. Published online October 3, 2019. doi:https://doi.org/10.1001/jamaoncol.2019.3394

20. Janjan NA, Wang XS, Mendoza TR, Zhang Y, Das P, Crane C, Delclos M, Krishnan S, Cleeland CS. Utility of the M. D. Anderson Symptom Inventory (MDASI) for symptom evaluation during chemoradiation in patients with gastrointestinal malignancies [abstract]. American Society of Clinical Oncology 43rd Annual Meeting, Chicago IL, Jun 1-5, 2007. J Clin Oncol 25(18 Suppl), 2007; 6613.

21. Liang WS, Craig DW, Carpten J, Borad MJ, Demeure MJ, Weiss GJ, Izatt T, Sinari S, Christoforides A, Aldrich J, Kurdoglu A, Barrett M, Phillips L, Benson H, Tembe W, Braggio E, Kiefer JA, Legendre C, Posner R1, Hostetter GH, Baker A, Egan JB, Han H, Lake D, Stites EC, Ramanathan RK, Fonseca R, Stewart AK, Von Hoff DD. Genome-wide characterization of pancreatic adenocarcinoma patients using massively parallel sequencing. PLos ONE. 2012 Oct 10;7(10):e43192. doi:10.1371/journal.pone.0043192.

22. Meadors N, Floyd J, Perry MC. Pulmonary toxicity of chemotherapy. Semin Oncol. 2006;33(1):98-105.

23. Moore MJ,Goldstein D, Hamm J, et al.: Erlotinib plus gemcitabine compared with gemcitabine alone in patients with advanced pancreatic cancer: a phase III trial of the National Cancer Institute of Canada Clinical Trials Group. J Clin Oncol. 2007;25:1960-1966.

24. Nab paclitaxel (Abraxane™) Prescribing Information (Updated 1/2012) – Accessed via Abraxane Website: http://www.abraxane.com/docs/Abraxane_PrescribingInformation.pdf

25. Reni M, Cereda S, Mazza E, Passoni P, Nicoletti R, Balzano G, Zerbi A, Arcidiacono PG, Staudacher C, Di Carlo V. PEGF (cisplatin, epirubicin, 5-fluorouracil, gemcitabine) regimen as second-line therapy in patients with progressive or recurrent pancreatic cancer after gemcitabine-containing chemotherapy. Am J Clin Oncol. 2008;31:145-150.

26. Rowinsky EK, Donehower RC. Paclitaxel (taxol). N. Engl J Med. 1995;332(15):1004-14.

27. Smith, T. J., Bohlke, K., Lyman, G. H., Carson, K. R., Crawford, J., Cross, S. J., . . . Armitage, J.O. (2015). Recommendations for the Use of WBC Growth Factors: American Society of Clinical Oncology Clinical Practice Guideline Update. *Journal of Clinical Oncology, 33*(28), 3199-3212. doi:10.1200/JCO.2015.62.3488.

28. Verbraecken J, Van De Heyning P, De Backer W, Van Gaal L. Body surface area in normal-weight, overweight, and obese adults. A comparison study. Metabolism. 2006;55(4):515-24.

29. Von Hoff DD, Ramanathan RK, Borad MJ, Laheru DA, Smith LS, Korn RL, Trieu V, Iglesias JL, Zhang H, Soon-Shiong P, Shi T, Rajeshkumar NV, Maiitra A, Hidalgo M. Gemcitabine plus nab-paclitaxel is an active regimen in patients with advanced pancreatic cancer: a phase I/II trial. J Clin Oncol. 2011;29;4548-4554.

30. Von Hoff DD, Ramanathan RK, Evans DB, Demeure MJ, Maney T, Wright B, Gatalica Z, McGinniss MJ. Actionable targets in pancreatic cancer detected by immunohistochemistry (IHC), microarray (MA) fluorescent in situ hybridization (FISH), and mutational analysis. J Clin Oncology. 2012;30s: abstr 4013.

31. Wang XS, Williams LA, Eng C, Mendoza TR, Shah NA, Kirkendoll KJ, Shah PK, Trask PC, Palos GR, Cleeland CS. Validation and application of a module of the M. D. Anderson Symptom Inventory for measuring multiple symptoms in patients with gastrointestinal cancer (the MDASI-GI). *Cancer* 116(8): 2053-2063, 2010.

APPENDIX A: SCHEDULE OF TREATMENT AND EVALUATIONS

	Screening Within 21 Days	Cycle 1					Cycle 2+					Prior to Cycles 4, 7, 10, etc.	End of Study or Early Termination[11]	Follow-up
**Assessment**		**Day 1**	**Day 2**	**Day 8**	**Day 9**	**Day 15**	**Day 1**	**Day 2**	**Day 8**	**Day 9**	**Day 15**			
Window		+7 days[2]		+ 1 day[2]		- 1 day	+7 days[2]		+ 1 day[2]		- 1 day			
Signed informed consent	X													
Signed Optional Tissue Informed Consent	X[8]													
Review inclusion/exclusion	X	X												
Medical history[1]	X	X												
Physical examination	X	X[3]		X[3]		X[3]	X[3]		X[3]				X	
Height (cm)	X													
Weight (kg)	X	X[3]		X[3]			X[3]		X[3]				X	
BSA calculation		X[3]					X[3,4]							
Vital signs	X	X[3]		X[3]		X[3]	X[3]		X[3]				X	
Karnofsky PS	X	X[3]		X[3]		X[3]	X[3]		X[3]				X	
CT/ MRI scan / tumor measurements[5]	X											X	X	
ECG	X													
CBC w/differential & PLTs	X	X[3]		X[3]		X[3]	X[3]		X[3]		X[3]		X	
Serum chemistries [6,14]	X	X[3]		X[3]		X[3]	X[3]		X[3]		X[3]		X	
PT/INR	X	X[3,17]					X[3,17]							
CA 19-9 (or CA 125, or CEA if not expressers of CA 19-9)	X	X[3]					X[3]						X	
Urinalysis[7]	X	X[3]					X[3]						X	
Fresh Tissue and/or Archived blocks of tumor[8]	X[8]													
Serum pregnancy	X	X[3]					X[3]						X	
Concomitant medications[9]	X	X[3]		X[3]		X[3]	X[3]		X[3]		X[3]		X	
Adverse events[10]		X[3]		X[3]		X[3]	X[3]		X[3]		X[3]		X	
Contact Information Review													X	X
Telephone follow-up[12]														X
MDASI-GI and BPI Questionnaires	x	x[3]					x[3]						x	

Assessment	Screening Within 21 Days	Cycle 1					Cycle 2+					Prior to Cycles 4, 7, 10, etc.	End of Study or Early Termination[11]	Follow-up
		Day 1	Day 2	Day 8	Day 9	Day 15	Day 1	Day 2	Day 8	Day 9	Day 15			
Window		+7 Days[2]		+ 1 day	-	- 1 day	+7 days[2]				- 1 day			
**TREATMENT**														
Nab-pacilitaxel[13]		X		X			X		X					
Cisplatin[13]		X		X			X		X					
Gemcitabine[13]		X		X			X		X					
Post Chemotherapy Hydration [15]		X	X		X			X	X	X				
Pegfilgrastim [16]				X					X					

Schedule of Events footnotes:

1. To include concurrent baseline conditions (using NCI CTCAE, version 5.0), prior cancer therapy (including documentation of prior surgery, adjuvant or neoadjuvant chemotherapy and radiotherapy).
2. Intended cycles may be delayed for non-toxicity reasons for up to 7 days (for reasons such as scheduling conflicts), but only with documentation and explanation in the CRF. Dosing on day 8 will not be delayed beyond + 1 day. Individual dosing days will not be delayed. If an individual dosing day needs to be delayed beyond +1 day, that particular dose is not to be administered and will be considered a missed dose.
3. All assessments should be performed preferentially on the same day of the study visit but may be performed within 1 day prior to study dose administration (on Days 1 and 8) if medical or scheduling conditions require.
4. BSA only needs to be changed if there has been a change >10% in body weight from Cycle 1-Day 1 (See Section 4.2).
5. Computed tomography (CT) / magnetic resonance imaging (MRI) scan to document disease status (including chest, abdomen, pelvis, and other regions as clinically indicated. In addition, a brain scan is required to exclude brain metastases if clinically indicated only. If a CT scan was taken within 28 days prior to first dose, a new scan is not necessary. However, if a new scan is to be done, it should be performed within 5 days of starting chemotherapy. In addition to the CT / MRI scan, tumor size may also be assessed utilizing visual or palpable lesions on physical examination including full assessment of all known metastases (see RECIST 1.1 criteria in Section 6). Follow-up scans are due every 3 cycles (prior to cycle 4, 7, 10)
6. To include BUN, phosphorus, magnesium, creatinine, total protein, albumin, calcium, glucose, total bilirubin, alkaline phosphatase, AST, ALT, and electrolytes (chloride, sodium, potassium, and bicarbonate) and CA 19-9 (or CA 125, or CEA if not expressers of CA 19-9).
7. Lab urinalysis to include protein, specific gravity, glucose, and blood.
8. Archived paraffin block of tumor specimen, metastatic tumor sample preferred. If patient is scheduled to undergo a tumor biopsy for standard of care purposes at any time, they should be consented with the Optional Tissue Collection and Storage Informed Consent Form.
9. To include all medications taken within 30 days prior to study enrollment.
10. Patients will be followed for all Grade ≥3 AEs, all grade neuropathy adverse events, any AE that leads to study treatment discontinuation and any AE that is associated with and contributing to an

SAE as determined by the Investigator regardless of causality, observed during the study period, starting with initial dose of study drug through the 30 days after patient's last dose of study drug, or until the beginning of a new anti-cancer therapy, whichever occurs first. The Investigator should follow AEs until the event is resolved or stabilized, the patient is lost to follow-up, or the event is otherwise explained.

11. End of Study or Early Termination assessments can be completed 14-28 (+/- 2) days from the last dose of study medication.
12. Follow-up assessments for survival in person or by telephone will be conducted in all patients 30 days after the End of Study or Early Termination Visit and then monthly for up to 12 months from the time of enrollment.
13. The sequence of drug administration is nab-paclitaxel, then cisplatin, then gemcitabine. Post cisplatin hydration: IV fluids up to 1000 mL (with additives as clinically indicated) IV given as infusion on days cisplatin is administered on days 1 and 8 repeated every 21 days. May start at the same time as the gemcitabine infusion. Volume may be reduced to 500 ml at the Investigator's discretion.
14. The Cockcroft-Gault equation may be used in calculating the creatinine clearance at any of the time points in this protocol.
15. Post cisplatin hydration: IV fluids up to 1000 mL (with additives as clinically indicated) IV given as infusion on days cisplatin is administered on days 1 and 8 repeated every 21 days. May start at the same time as the gemcitabine infusion. Additional post-cisplatin hydration of 1 liter of IV fluids on days 2 & 9 required for Cycles 1-3. Volume may be reduced to 500 ml at the Investigator's discretion. Note: If the cisplatin is held in any cycle, the additional hydration on day of chemotherapy or day after would not be needed.
16. Pegfilgrastim will be administered subcutaneously on day 9 of each treatment cycle. Neulasta OnPro® may be given on Day 8 if patient is not returning for Day 9 hydration.
17. PT/INR required only If patient is on anticoagulation with warfarin.

APPENDIX B: KARNOFSKY PERFORMANCE STATUS

Karnofsky Performance Status		
	**Score**	**Descriptions**
Able to carry on normal activity and to work; no special care needed.	100	Normal: no complaints; no evidence of disease
	90	Able to carry on normal activity; minor signs or symptoms of disease
	80	Normal activity with effort; some signs or symptoms of disease
Unable to work; able to live at home and care for most personal needs; varying amount of assistance needed.	70	Cares for self; unable to carry on normal activities or to do active work
	60	Requires occasional assistance, but is able to care for most of personal needs
	50	Requires considerable assistance and frequent medical care
Unable to care for self; requires equivalent of institutional or hospital care; disease may be progressing rapidly.	40	Disabled: requires special care and assistance
	30	Severely disabled: hospital admission is indicated although death not imminent
	20	Very sick: hospital admission necessary; active supportive treatment needed
	10	Moribund: fatal processes are progressing rapidly
	0	Dead

APPENDIX C: REVISED NATIONAL CANCER INSTITUTE COMMON TERMINOLOGY CRITERIA FOR ADVERSE EVENTS (NCI CTCAE), VERSION 5.0 (PUBLISHED 27 NOVEMBER 2017)

The National Cancer Institute (NCI) Cancer Therapy Evaluation Program (CTEP) Common Terminology Criteria for Adverse Events (CTCAE), version 5.0 can be viewed on-line at the following NCI web site:

https://ctep.cancer.gov/protocoldevelopment/electronic_applications/docs/CTCAE_v5_Quick_Reference_8.5x11.pdf

APPENDIX D: DRUG STUDY DRUG PREPARATION, DOSING, ADMINISTRATION AND STORAGE

See links below to website access

Nab paclitaxel (Abraxane™) Prescribing Information (Updated 8/2020) – Accessed via Abraxane Website: https://media.celgene.com/content/uploads/abraxane-pi.pdf

Cisplatin Prescribing Information (Updated 06/01/2015) – Accessed via Daily Med (National Library of Medicine) http://dailymed.nlm.nih.gov/dailymed/lookup.cfm?setid=a440f077-46f6-4688-a209-65bce38d1c92

Gemcitabine (Gemzar™) Prescribing Information (Updated 5/2019) – Accessed via Gemzar Website: http://pi.lilly.com/us/gemzar.pdf

APPENDIX E: THE MD ANDERSON SYMPTOM INVENTORY GASTROINTESTINAL CANCER MODULE (MDASI-GI) (TO BE SUBMITTED SEPARATELY FROM PROTOCOL FOR IRB APPROVAL)

MD Anderson Symptom Inventory (MDASI) modules augment the 19 core MDASI symptom and interference items with additional items identified as unique to a particular patient population. MDASI modules may be disease-specific, disease-site-specific, or treatment-specific.
The MDASI gastrointestinal cancer module (MDASI-GI) is a site-specific module. Along with the core MDASI's 13 symptom items and 6 interference items, the MDASI-GI also assesses 5 symptoms specific to gastrointestinal cancer:

- Constipation
- diarrhea or watery stools
- difficulty swallowing
- change in taste
- feeling bloated

MDASI-GI Features

- Purpose: To assess the severity of multiple gastrointestinal cancer-related symptoms and the impact of these symptoms on daily functioning
- Population: Patients with symptoms caused by gastrointestinal cancer and its treatment
- Assessment areas: Severity of multiple symptoms and the impact of symptoms on daily functioning during the last 24 hours
- Method: Self-report; paper-and-pencil form or tablet PC (self-administered or via interview), or telephone-based interactive voice response (IVR) system
- Time required: Five minutes
- Scoring: Please see the MDASI User's Guide
- Reliability: Cronbach alpha reliability ranges from 0.80 to 0.87

APPENDIX F: THE BRIEF PAIN INVENTORY (BPI) – SHORT FORM (TO BE SUBMITTED SEPARATELY FROM PROTOCOL FOR IRB APPROVAL)

The Brief Pain Inventory (BPI) is available in two formats: the BPI short form, which is used for clinical trials and is the version used for the foreign-language translations; and the BPI long form, which contains additional descriptive items that may be clinically useful (for example, items that expand the possible descriptors of pain, such as burning, tingling, etc.). For brevity's sake and for the patient's ease of use, however, we recommend the short form of the BPI.

In response to the US Food and Drug Administration (FDA) guidance for the pharmaceutical industry on the use of patient-reported outcome measures in medical product development to support labeling claims, we have prepared a BPI User's Guide to provide documentation of the BPI's development and psychometric properties. The information offered therein addresses the recommendations in the FDA guidance and establishes the BPI's adequacy as a measure to support medical product claims.

BPI Features

- Purpose: To assess the severity of pain and the impact of pain on daily functions
- Population: Patients with pain from chronic diseases or conditions such as cancer, osteoarthritis and low back pain, or with pain from acute conditions such as postoperative pain
- Assessment areas: Severity of pain, impact of pain on daily function, location of pain, pain medications and amount of pain relief in the past 24 hours or the past week
- Responsiveness: Responds to both behavioral and pharmacological pain interventions
- Method: Self-report or interview
- Time required: Five minutes (short form), 10 minutes (long form)
- Scoring: No scoring algorithm, but "worst pain" or the arithmetic mean of the four severity items can be used as measures of pain severity; the arithmetic mean of the seven interference items can be used as a measure of pain interference
- Reliability: Cronbach alpha reliability ranges from 0.77 to 0.91

APPENDIX G: 2006 UPDATE OF ASCO PRACTICE GUIDELINE RECOMMENDATIONS FOR THE USE OF WHITE BLOOD CELL GROWTH FACTORS: GUIDELINE SUMMARY

Setting/Indication	Recommendation
Primary prophylaxis	Primary prophylaxis is recommended for the prevention of FN in patients who have a high risk of FN based on age, medical history, disease characteristics, and myelotoxicity of the chemotherapy regimen. For "dose-dense" regimens, CSF is required and recommended. Clinical trial data support the use of CSF when the risk of FN is in the range of 20% or higher.
Primary prophylaxis: Special circumstances	Certain clinical factors predispose to increased complications from prolonged neutropenia, including: patient age > 65 years; poor performance status; previous episodes of FN; extensive prior treatment including large radiation ports; administration of combined chemoradiotherapy; bone marrow involvement by tumor-producing cytopenias; poor nutritional status; the presence of open wounds or active infections; more advanced cancer, as well as other serious comorbidities. In such situations, primary prophylaxis with CSF is often appropriate, even with regimens with FN rates of < 20%.
Secondary prophylaxis	Secondary prophylaxis with CSF is recommended for patients who experienced a neutropenic complication from a prior cycle of chemotherapy (for which primary prophylaxis was not received), in which a reduced dose may compromise disease-free or overall survival or treatment outcome. In many clinical situations, dose reduction or delay may be a reasonable alternative.
Therapeutic use: Afebrile neutropenia	CSF should not be routinely used for patients with neutropenia who are afebrile.
Therapeutic use: Febrile neutropenia	CSF should not be routinely used as adjunctive treatment with antibiotic therapy for patients with fever and neutropenia. However, CSF should be considered in patients with fever and neutropenia who are at high-risk for infection-associated complications, or who have prognostic factors that are predictive of poor clinical outcomes. High-risk features include expected prolonged (> 10 days) and profound (< 0.1 × 109/L) neutropenia, age > 65 years, uncontrolled primary disease, pneumonia, hypotension and multi-organ dysfunction (sepsis syndrome), invasive fungal infection, or being hospitalized at the time of the development of fever.
Dose intensity/density of chemotherapy	Dose-dense regimens should only be used within an appropriately designed clinical trial or if supported by convincing efficacy data.

Setting/Indication	Recommendation
Adjuncts to progenitor-cell transplantation	Administration of CSF to mobilize PBPC often in conjunction with chemotherapy, and their administration after autologous, but not allogeneic, PBPC transplantation is the current standard of care.
AML: Initial or repeat induction chemotherapy	CSF use following initial induction therapy is reasonable, though there has been no favorable impact on remission rate, remission duration, or survival. Patients > 55 years of age may be most likely to benefit from CSF use.
AML: CSF for priming effects	Use of CSF for priming effects is not recommended.
AML: Consolidation chemotherapy	CSF use can be recommended after the completion of consolidation chemotherapy because of the potential to decrease the incidence of infection and eliminate the likelihood of hospitalization in some patients receiving intensive post remission chemotherapy. There seems to be more profound shortening of the duration of neutropenia after consolidation chemotherapy for patients with AML in remission than for patients receiving initial induction therapy. As yet there is no information about the effect of longer-acting pegylated CSFs in patients with myeloid leukemias, and they should not be used in such patients outside of clinical trials.
MDS	Intermittent administration of CSF may be considered in a subset of patients with severe neutropenia and recurrent infection.
ALL	CSF administration is recommended after the completion of the initial first few days of chemotherapy of the initial induction or first post remission course, thus shortening the duration of neutropenia of < 1,000/mm^3 by approximately 1 week.
Acute leukemia in relapse	CSF should be used judiciously, or not at all, in patients with refractory or relapsed myeloid leukemia since the expected benefit is only a few days of shortened neutropenia.
Radiotherapy ± chemotherapy	CSF should be avoided in patients receiving concomitant chemotherapy and radiation therapy, particularly involving the mediastinum. In the absence of chemotherapy, therapeutic use of CSF may be considered in patients receiving radiation therapy alone if prolonged delays secondary to neutropenia are expected.
Older patients	Prophylactic CSF for patients aged ≥ 65 years with lymphoma treated with curative chemotherapy (CHOP or more aggressive regimens) should be given to reduce the incidence of FN and infections.
Pediatric patients	As in adults, the use of G-CSF is reasonable for the primary prophylaxis of pediatric patients with a likelihood of FN. Similarly,

Setting/Indication	Recommendation
	the use of G-CSF for secondary prophylaxis or for therapy should be limited to high-risk patients. However, the potential risk for secondary myeloid leukemia or myelodysplastic syndrome associated with G-CSF represents a concern in children with ALL whose prognosis is otherwise excellent. For these reasons, the specific use of G-CSF in children with ALL should be considered carefully.
Comparative clinical activity of G-CSF and GM-CSF	No guideline recommendation can be made regarding the equivalency of the two colony-stimulating agents. Further trials are recommended to study the comparative clinical activity, toxicity, and cost-effectiveness of GCSF and GM-CSF.
Treatment for radiation injury	Current recommendations for the management of patients exposed to lethal doses of total-body radiotherapy, but not doses high enough to lead to certain death due to injury to other organs, includes the prompt administration of CSF or pegylated G-CSF.

Abbreviations: CSF, colony-stimulating factors; FN, febrile neutropenia; PBPC, peripheral-blood progenitor cell; AML, acute myeloid leukemia; MDS, myelodysplastic syndrome; ALL, acute lymphocytic leukemia; CHOP, cyclophosphamide, doxorubicin, vincristine, prednisone; G-CSF, filgrastim; GM-CSF, sargramostim; pegylated G-CSF, pegfilgrastim.
