# Supplementary material for: Phase II clinical trial of nab‐paclitaxel plus cisplatin plus gemcitabine (NABPLAGEM) in patients with untreated advanced pancreatic cancer
Source: Cancer Med. 2024 Jun 22;13(12):e7412. doi: 10.1002/cam4.7412 (PMC11192994; doi:10.1002/cam4.7412)
Supplement: Supplementary file 2 — Data S2. [file CAM4-13-e7412-s002.docx]

**Supplemental Contents**

[Supplement 1. Study Protocol 2](#_Toc167877253)

[Supplement 2. 3](#_Toc167877254)

[eTable 1. Patient Detail 3](#_Toc167877255)

[eFigure 1. Overall Survival in NAPLAGEM trial by HRD Mutation 3](#_Toc167877256)

[eAppendix 1. Methods for the tissue analysis for comparative genomic hybridization (CGH) and next generation sequencing (NGS) 4](#_Toc167877257)

[eAppendix 2. 01-21 Tumor Analysis 6](#_Toc167877258)

[eFigure 2. DNA content flow sorting and genome profile of AXL-PANC 01-21 6](#_Toc167877259)

[eFigure 3. Mutation signatures in flow sorted tumor fraction of AXL-PANC 01-21. 7](#_Toc167877260)

[eTable 2. Adverse events (grade ≥ 3) that were possibly, probably, or definitely related to study drug or study procedure, by severity (highest grade per person, per preferred term), n (%) 8](#_Toc167877261)

[eTable 3. List of serious adverse events that were possibly, probably, or definitely related to one or more study drugs. 8](#_Toc167877262)

[eAppendix 3. SAE Narratives 9](#_Toc167877263)

[eTable 4. MDASI-GI scores at baseline, midpoint (C4/D1), and end of study; mean ± SD 11](#_Toc167877264)

[eTable 5. Sensitivity Analysis: MDASI-GI scores at baseline, midpoint (C4/D1), and end of study among patients with baseline scores > 0 11](#_Toc167877265)

[eTable 6. BPI scores at baseline, midpoint (C4/D1), and end of study: mean ± SD 11](#_Toc167877266)

[eTable 7. Sensitivity Analysis: BPI scores at baseline, midpoint (C4/D1), and end of study among participants with baseline scores > 0 11](#_Toc167877267)

[eFigure 4. Swimmer Plot, Sorted by Survival Time 12](#_Toc167877268)

[eFigure 5. Swimmer Plot, Sorted by Time on Treatment 12](#_Toc167877269)

[eTable 8. Comparison of Phase IB/II Pilot to Phase II 13](#_Toc167877270)

[eTable 9. Study drug dosing, by trial 14](#_Toc167877271)

**Supplemental Material**

# Supplement 1. Study Protocol

# Supplement 2.

This supplementary material has been provided by the authors to give readers additional information about their work.

# eTable 1. Patient Detail

# eFigure 1. Overall Survival in NAPLAGEM trial by HRD Mutation


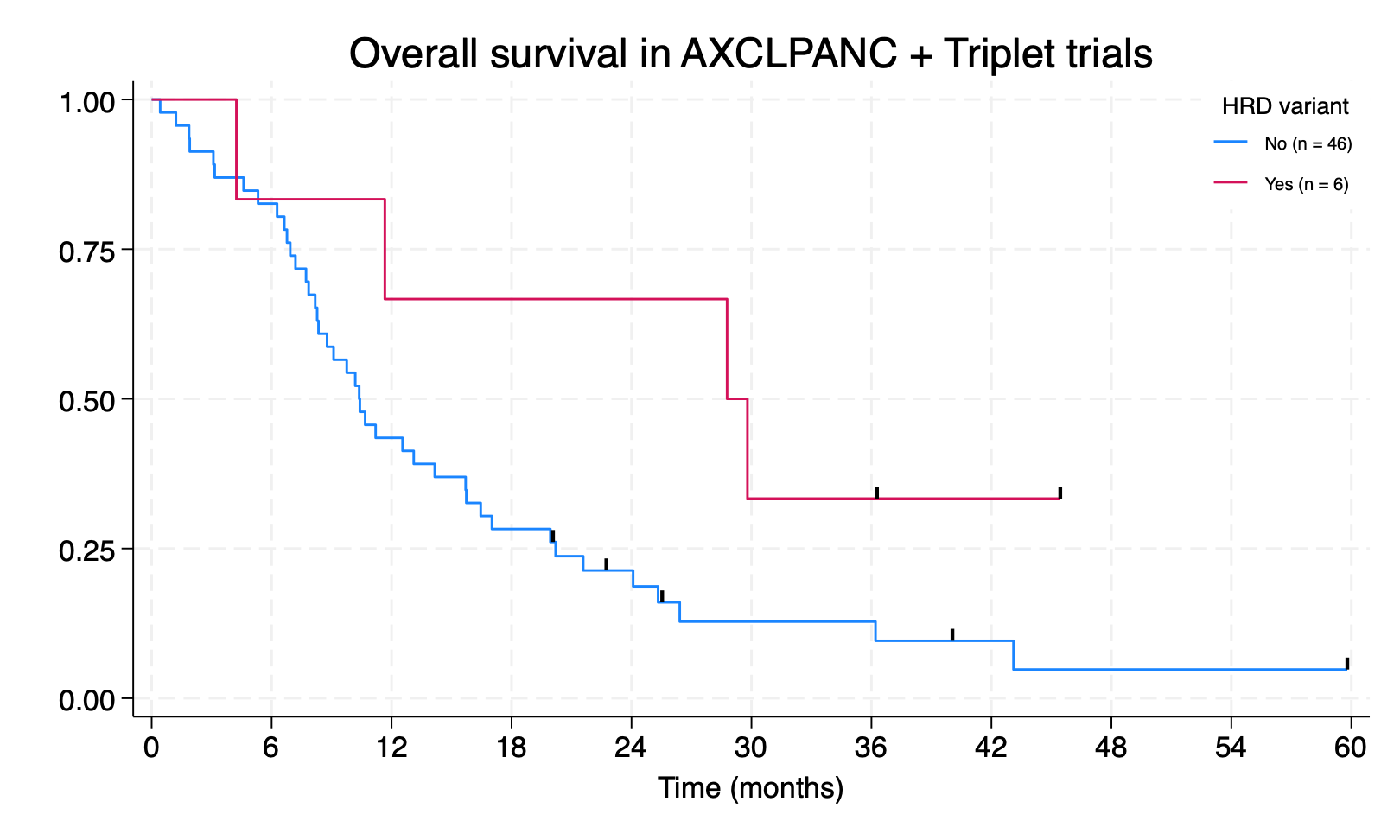


Median (95% CI) survival, stratified by mutation status:

No HRD variants: 10.4 (8.2–15.7) months

Yes HRD variants: 28.8 (4.2– .) months

Cox proportional hazards regression model:

Unadjusted, HR = 0.39 (p = 0.078)

Adjusted for study, HR = 0.48 (p = 0.179)

# eAppendix 1. Methods for the tissue analysis for comparative genomic hybridization (CGH) and next generation sequencing (NGS)

**Flow Cytometry:** For each FFPE sample, excess paraffin was removed with a scalpel from either side of 40-60 μm scrolls then processed according to our published methods.^1, 2^ We used one to three 50 µm scroll(s) from each FFPE tissue block to obtain sufficient numbers of intact nuclei for sorting and molecular assays. Frozen tissue samples were minced in the presence of NST buffer and DAPI according to published protocols.^1, 3, 4^ Nuclei from each sample were disaggregated then filtered through a 40 μm mesh prior to flow sorting with an Influx or Aria III cytometer (Becton-Dickinson, San Jose, CA) with ultraviolet excitation and DAPI emission collected at >450 nm. DNA content and cell cycle were analyzed using the software program MultiCycle (Phoenix Flow Systems, San Diego, CA).

**aCGH:** All aCGH was done according to our published protocols.^1, 4, 5, 6^ Briefly, DNAs from frozen tissue and FFPE samples were treated with DNase 1 prior to Klenow-based labeling. High molecular weight templates were digested for 30 minutes while DNAs from FFPE samples were digested for only 1 minute. In each case 1 µL of 10x DNase 1 reaction buffer and 2 μL of DNase 1 dilution buffer were added to 7 μL of DNA sample and incubated at room temperature then transferred to 70°C for 30 minutes to deactivate DNase 1. Sample and reference templates were then labeled with Cy-5 dUTP and Cy-3 dUTP respectively using a BioPrime labeling kit (Invitrogen, Carlsbad, CA) according to our published protocols^5^. All labeling reactions were assessed using a Nanodrop assay (Nanodrop, Wilmington, DE) prior to mixing and hybridization to 400k CGH arrays (Agilent Technologies, Santa Clara, CA) for 40 hours in a rotating 65°C oven. All microarray slides were scanned using an Agilent 2565C DNA scanner, and the images were analyzed with Agilent Feature Extraction version 11.0 using default settings. The aCGH data were assessed with a series of QC metrics, then analyzed using an aberration detection algorithm (ADM2)^7^. The latter identifies all aberrant intervals in a given sample with consistently high or low log ratios based on the statistical score derived from the average normalized log ratios of all probes in the genomic interval multiplied by the square root of the number of these probes. This score represents the deviation of the average of the normalized log ratios from its expected value of zero and is proportional to the height h (absolute average log ratio) of the genomic interval, and to the square root of the number of probes in the interval. All data have been deposited to GEO repository (accession GSE233746).

**Whole genome sequencing:** DNAs from sorted tumor population and patient matched control sample were sequenced within the Mayo Clinic Medical Genome Facility (MGF). Libraries were prepared using Nextera DNA Flex library prep kit. Samples were then sequenced on Illumina NovaSeq S4. Genome_GPS v5.0.3 (formerly named as TREAT^8^) was used as a comprehensive secondary analysis pipeline for DNA sequencing data at Mayo Clinic. FASTQ files were aligned to the hg38 reference genome using bwa-mem (VN:V0.7.10) using default options. Realignment was performed using GATK (VN:3.4–46)^9^. Multisample variant calling was performed using the GATK (VN: 3.4–46) Haplotype Caller, and variants were filtered using variant recalibration Variant Quality Score Recalibrator for both single nucleotide variants and insertions and deletions. To call somatic mutations, the workflow uses a combination of Mutect2^10^ and Strelka2^11^. Identified variants were annotated using BioR^5, 12^ framework with functional features, impact prediction, and clinical significance using Clinical Annotation of Variants, ClinVar, Human Gene Mutation Database, Mayo Biobank, and Exome Aggregation Consortium population frequencies. All data have been submitted to NIH Sequence Read Archive (SRA) (accession number pending).

**References**

1. Holley, T., et al., *Deep clonal profiling of formalin fixed paraffin embedded clinical samples.* PLoS One, 2012. **7**(11): p. e50586.

2. Barrett, M.T., et al., *Genomic amplification of 9p24.1 targeting JAK2, PD-L1, and PD-L2 is enriched in high-risk triple negative breast cancer.* Oncotarget, 2015. **6**(28): p. 26483-93.

3. Rabinovitch, P.S., et al., *Predictors of progression in Barrett's esophagus III: baseline flow cytometric variables.* Am J Gastroenterol, 2001. **96**(11): p. 3071-83.

4. Barrett, M.T., et al., *Clinical study of genomic drivers in pancreatic ductal adenocarcinoma.* Br J Cancer, 2017. **117**(4): p. 572-582.

5. Ruiz, C., et al., *Advancing a clinically relevant perspective of the clonal nature of cancer.* Proc Natl Acad Sci U S A, 2011. **108**(29): p. 12054-9.

6. Lenkiewicz, E., et al., *Genomic and Epigenomic Landscaping Defines New Therapeutic Targets for Adenosquamous Carcinoma of the Pancreas.* Cancer Res, 2020. **80**(20): p. 4324-4334.

7. Lipson, D., et al., *Efficient calculation of interval scores for DNA copy number data analysis.* J Comput Biol, 2006. **13**(2): p. 215-28.

8. Asmann, Y.W., et al., *TREAT: a bioinformatics tool for variant annotations and visualizations in targeted and exome sequencing data.* Bioinformatics, 2012. **28**(2): p. 277-8.

9. McKenna, A., et al., *The Genome Analysis Toolkit: a MapReduce framework for analyzing next-generation DNA sequencing data.* Genome Res, 2010. **20**(9): p. 1297-303.

10. Cibulskis, K., et al., *Sensitive detection of somatic point mutations in impure and heterogeneous cancer samples.* Nat Biotechnol, 2013. **31**(3): p. 213-9.

11. Kim, S., et al., *Strelka2: fast and accurate calling of germline and somatic variants.* Nat Methods, 2018. **15**(8): p. 591-594.

12. Kocher, J.P., et al., *The Biological Reference Repository (BioR): a rapid and flexible system for genomics annotation.* Bioinformatics, 2014. **30**(13): p. 1920-2.

# eAppendix 2. 01-21 Tumor Analysis

Patient 01-21 was a 52 year old female with liver metastasis who experienced disease progression after 2 cycles of study treatment with growth in liver lesions and new onset of symptoms consistent with brain metastasis. The sorted aneuploid fraction of sample 01-21 had a prominent 3.1N population with copy number variants (CNVs) including gain of 12p, losses of 17p, 18q11-qtel, and 9p21-ptel. The latter included a focal homozygous deletion of CDKN2A. In contrast, the co-existing sorted diploid fraction was CNV normal. Notably, we discriminated copy number polymorphisms, gains and losses, that were present in the 2.0N fraction from somatic CNVs in the 3.1N tumor population. The tumor had a low tumor mutation burden (TMB) of 4 mut/Mb and was microsatellite stable (MSS). Somatic mutations including KRASG12V, and a 6bp insertion in M237 exon 6 of TP53. The latter is a “hot spot” for pathogenic variants including indels. Mutational signatures included single base substitution (SBS) 1 and 5. The former is associated with cell division and biological clock related to aging in adult onset tumors, while SBS5 has an unknown etiology associated with tobacco exposure. Indel signatures (IDs) included ID1, 2, and 8 that are linked to DNA repair deficiencies. Double base substitution (DBS) signature DBS9 of unknown etiology, was also present. However, there was no evidence for homologous recombination deficiency (HRD) such as pathogenic variants and deletions in BRCA1/2 and related genes. Furthermore, the overall burden of copy number lesions was relatively low compared to known HRD cases.

# eFigure 2. DNA content flow sorting and genome profile of AXL-PANC 01-21

A) DNA content flow cytometry histogram identified a diploid (2.0N) peak (P2) and an aneuploid peak (P3) in biopsy. B) Whole genome CNV plots of flow sorted diploid and aneuploid fractions. The aneuploid tumor fraction has a KRASG12V variant, a homozygous deletion of CDKN2A, and a 6bp insertion in TP53 exon 3.

# eFigure 3. Mutation signatures in flow sorted tumor fraction of AXL-PANC 01-21.

A) Single base substitution (SBS) signatures include SBS1 and SBS5. B) Insertion/deletion (ID) signatures include ID1, ID8, and ID12. C) Double base substitution (SBS) signature SBS9.

# eTable 2. Adverse events (grade ≥ 3) that were possibly, probably, or definitely related to study drug or study procedure, by severity (highest grade per person, per preferred term), n (%)

| **Preferred term** | **Grade 3** | **Grade 4** | **Grade 5** |
| --- | --- | --- | --- |
| Acute kidney injury | 1 (2.4) | 0 | 0 |
| Anemia | 20 (47.6) | 0 | 0 |
| Anorexia | 1 (2.4) | 0 | 0 |
| Colitis | 2 (4.8) | 0 | 0 |
| Diarrhea | 5 (11.9) | 0 | 0 |
| Enterocolitis | 1 (2.4) | 0 | 0 |
| Fatigue | 3 (7.1) | 0 | 0 |
| Gait disturbance | 1 (2.4) | 0 | 0 |
| Generalized muscle weakness | 1 (2.4) | 0 | 0 |
| Hypocalcemia | 1 (2.4) | 0 | 0 |
| Hypokalemia | 4 (9.5) | 0 | 0 |
| Hyponatremia | 0 | 1 (2.4) | 0 |
| Lymphocyte count decreased | 1 (2.4) | 0 | 0 |
| Mucositis oral | 1 (2.4) | 0 | 0 |
| Neutrophil count decreased | 7 (16.7) | 4 (9.5) | 0 |
| Peripheral sensory neuropathy | 3 (7.1) | 0 | 0 |
| Platelet count decreased | 11 (26.2) | 16 (38.1) | 0 |
| Skin infection | 1 (2.4) | 0 | 0 |
| White blood cell decreased | 6 (14.3) | 1 (2.4) | 0 |

# eTable 3. List of serious adverse events that were possibly, probably, or definitely related to one or more study drugs.

| **Patient** | **Grade** | **Serious adverse event (SAE)** | **Nab-paclitaxel** | **Cisplatin** | **Gemcitabine** |
| --- | --- | --- | --- | --- | --- |
| 01-05 | 3 | Enterocolitis | Probably | Unlikely | Unlikely |
| 01-06 | 3 | Diarrhea | Probably | Possibly | Possibly |
| 01-13 | 4 | Hyponatremia | Possibly | Possibly | Possibly |
| 01-21 | 3 | Colitis | Probably | Unlikely | Unlikely |
| 02-01 | 4 | Platelet count decreased | Definitely | Definitely | Definitely |
| 02-05 | 3 | Skin infection | Probably | Probably | Probably |
| 02-07 | 3 | Acute kidney injury | Probably | Possibly | Probably |
| 02-09 | 4 | Platelet count decreased | Definitely | Definitely | Definitely |

# eAppendix 3. SAE Narratives

**Treatment-Related SAE Narratives**

**01-05 Enterocolitis Grade 3:** A 70-year-old female during Cycle 1, the patient experienced abdominal pain with diarrhea, grade 4 thrombocytopenia, grade 4 chemotherapy induced neutropenia and was admitted to hospital. CT scan performed confirmed grade 3 enterocolitis. Patient received treatment with morphine, hydrocortisone, antibiotics (cefepime and vancomycin) and metronidazole during hospitalization. Patient was discharged and resumed study treatment on C2/D1.

**01-06 Diarrhea Grade 3**: A 79-year-old male during Cycle 1 experienced grade 3 diarrhea, grade 2 abdominal pain, and generalized weakness, grade 3 thrombocytopenia and was admitted to hospital. Chest x-ray and CT were all clinically non-significant. Diarrhea resolved and patient was discharged with ongoing grade 2 abdominal pain. Patient chose not to resume study treatment and was followed for survival status.

**01-13 Hyponatremia Grade 4**: A 78-year-old male during Cycle 1 experienced grade 4 hyponatremia (serum sodium 119 mmol/L) and was admitted to the hospital. During hospitalization, patient was placed on fluid restriction and was treated with NS and was later discharged. The patient resumed study treatment on C2/D1.

**01-21 Colitis Grade 3**: A 52-year-old female during Cycle 2 experienced grade 3 colitis, and chemotherapy induced grade 3 anemia and was admitted to hospital. CT scan confirmed mild to moderate colitis, lactoferrin lab was positive, negative for C.diff, and GI bleed was ruled out. The patient was treated with loperamide. MRI of brain and spine showed multiple new intracranial metastasis and bone lesions. The patient was taken off study treatment due to disease progression and was followed for survival status.

**02-01 Thrombocytopenia Grade 4**: A 67-year-old female during C4/D15 experienced grade 4 thrombocytopenia with platelet count of 6,000. As the patient would not accept blood products due to observed religion, the investigator assessed this SAE to meet the criteria of Medically Significant Event. The SAE resolved with platelets reported as 218,000 and the patient resumed study treatment on C5/D1.

**02-05 Skin Infection Grade 3:** A 67-year-old male during Cycle 7 reported an abscess on thigh that had worsened and developed redness and swelling, and fever of 101.8°F. The patient had a history of scratching due to anxiety. The patient was sent to the ER to have it assessed and treated. The patient was diagnosed with cellulitis and admitted to the hospital (C7/D4) and received IV vancomycin and had the abscess drained and an excisional debridement performed. The patient was discharged from the hospital after 4 days on oral antibiotics (levofloxacin and doxycycline). The patient did not resume study treatment as CT scans performed showed disease progression. The patient was taken off study treatment due to disease progression and was followed for survival status.

**02-07 Acute Kidney Injury Grade 3**: A 60-year-old male on C1/D5 reported generalized weakness, hiccups, and fever of 101°F. Patient felt otherwise well, and temperature normalized with acetaminophen. The symptoms were felt to be chemotherapy related and disease related. On C1/D6, the patient reported a return of the fever, a pulse of 113 bpm, and a BP of 84/52 mmHg, and it was advised that patient should go to the ER. Upon presentation, the patient was found to be hypotensive and tachycardic with atrial fibrillation and rapid ventricular response. His labs included grade 3 anemia, grade 3 neutropenia, grade 3 lymphopenia, impaired renal function with electrolyte abnormalities consistent with grade 3 acute kidney injury as determined with nephrology. He was started on IV fluids and norepinephrine, empiric antibiotics, and IV bicarbonate. He also received rasburicase for hyperuricemia. Overall, his condition improved within 48 hours of hospitalization. He did not require supplemental oxygen. He was discharged in stable condition and on oral antibiotics for a urinary tract infection. He was to resume study treatment at a reduced dose on C2/D1. The medical monitor reviewed the SAE and determined that given cisplatin is potentially nephrotoxic, and patients with advanced pancreatic cancer are frequently dehydrated due to disease-related gastrointestinal symptoms, they may be at higher risk of renal toxicity. The protocol was then amended to require the previously optional cisplatin post hydration on days 2 and 9 for Cycles 1-3.

**02-09 Thrombocytopenia Grade 4**: A 61-year-old female during Cycle 10/ D15 experienced grade 4 thrombocytopenia, platelet count of 8,000. Patient denied bruising and bleeding. Platelet transfusion was administered and patient C11/D1 labs reported platelet count of 100,000. Patient resumed study treatment in C11/D1.

Patient tended to have decreased platelet on Day 15 of the cycle and received platelet transfusion, however platelet count had never been < 10,000. For this reason, the treating investigator reported this as an SAE because it was thought, with a platelet count < 10,000, the patient was at risk for a spontaneous intra-cranial bleed.

**Unrelated Grade 5 SAE Narratives**

**02-03 Cardiac Arrest**: A 65-year-old male started study treatment and returned for C1/D2 hydration, vitals were normal apart from blood pressure that was elevated (140/92), patient reported loose bowel movement and one episode of stool incontinence that was attributed to laxatives taken for opioid-induced constipation. On C1/D3 the spouse called to report patient had increased abdominal pain, and the patient was advised to increase pain medications. On C1/D8 spouse reported nausea, and prochlorperazine was added to ondansetron that was already being taken. The treating oncologist attempted to arrange a telehealth visit for that day but authorization for this visit could not be obtained. Patient was evaluated by PCP on that day, and documentation from that visit noted the patient felt weak, nauseated, lethargic, and had abdominal discomfort with bloating. He did not report fever or chills but did report night sweats. The vitals taken at that visit included: Temp 98.5°F, HR of 72 bpm, BP of 110/60 mmHg and RR of 12 bpm. PCP decreased clonidine tablets from 0.3 mg to 0.2 mg 3 times a day and advised patient to continue taking lisinopril 20 mg daily. At home that night the patient continued to have abdominal pain and woke up complaining of pain and called 911 early morning on C1/D9. The spouse reported the patient was alert and interactive when he left the house with the paramedics. The documentation from the ED stated the patient arrived unresponsive in cardiac arrest and resuscitation was initiated. There was no documentation of heart rhythm, and no vital signs are documented by paramedics or the ED. There was no EKG or laboratory tests obtained in the ED, nor did the patient ever achieve return of spontaneous circulation. The patient’s medical history included: pancreatic cancer with metastasis to the liver, splenic vein thrombosis, hypertension, diabetes, and obesity (BMI of 30). Based on the information that was collected a direct cause of death was not confirmed.

Upon review the investigator and medical monitor determined that the cardiac arrest was unlikely related to the study treatment and possibly related to the pancreatic cancer, given the interval of time from treatment to the event, and that the patient may have experienced a catastrophic event as a result of either progression of disease or regression in the setting of response to therapy, possibly associated with bleeding or perforation.

**01-11 COVID-19 Viremia**: A 76-year-old female during Cycle 3 had been exposed to COVID-19 by infected family member and tested positive but was originally asymptomatic. The patient eventually developed fever (104°F) and was sent to the ED and admitted to the hospital due to low oxygen. CT of chest showed extensive bilateral peripheral prominent ground glass consolidation involving all lobes of both lungs, consistent with COVID-19 pneumonia. Patient was treated with dexamethasone (10 mg IV q 24 hr), vancomycin (1000 mg IV q 12 hr) and administered 1 unit of COVID convalescent plasma. Patient further deteriorated and was transferred to ICU, was intubated, and later died due to respiratory failure and multi-organ failure from viremia related to COVID-19. The patient’s medical history included: pancreatic cancer with metastasis to the liver, type 2 diabetes, and hypertension.

# eTable 4. MDASI-GI scores at baseline, midpoint (C4/D1), and end of study; mean ± SD

| **Subscale** | **Baseline** | **C4/D1** | **C4/D1 imputed** | **End of study** |
| --- | --- | --- | --- | --- |
| Core symptom severity |  |  |  |  |
| *n* | 42 | 28 | 42 | 42 |
| mean ± SD | 2.5 ± 1.7 | 1.5 ± 1.5 | 2.0 ± 1.9 | 2.5 ± 2.0 |
| Overall symptom distress (interference) |  |  |  |  |
| *n* | 42 | 28 | 42 | 42 |
| mean ± SD | 3.1 ± 2.9 | 2.0 ± 2.5 | 2.8 ± 3.0 | 3.2 ± 3.0 |
| Gastrointestinal module |  |  |  |  |
| *n* | 42 | 28 | 42 | 42 |
| mean ± SD | 1.8 ± 1.7 | 1.5 ± 1.4 | 1.7 ± 1.6 | 1.8 ± 1.6 |

#

# eTable 5. Sensitivity Analysis: MDASI-GI scores at baseline, midpoint (C4/D1), and end of study among patients with baseline scores > 0

| **Subscale** | **Baseline** | **C4/D1** | **C4/D1 imputed** | **End of study** |
| --- | --- | --- | --- | --- |
| Core symptom severity |  |  |  |  |
| *n* | 40 | 27 | 40 | 40 |
| mean ± SD | 2.6 ± 1.6 | 1.5 ± 1.6 | 2.0 ± 1.9 | 2.5 ± 2.1 |
| Overall symptom distress (interference) |  |  |  |  |
| *n* | 36 | 23 | 36 | 36 |
| mean ± SD | 3.6 ± 2.8 | 2.2 ± 2.6 | 2.9 ± 3.1 | 3.4 ± 3.0 |
| Gastrointestinal module |  |  |  |  |
| *n* | 33 | 21 | 33 | 33 |
| mean ± SD | 2.4 ± 1.6 | 1.5 ± 1.2 | 1.8 ± 1.5 | 2.0 ± 1.7 |

# eTable 6. BPI scores at baseline, midpoint (C4/D1), and end of study: mean ± SD

| **Subscale** | **Baseline** | **C4/D1** | **C4/D1 imputed** | **End of study** |
| --- | --- | --- | --- | --- |
| Pain severity |  |  |  |  |
| *n* | 42 | 28 | 42 | 42 |
| mean ± SD | 2.7 ± 2.1 | 1.1 ± 1.5 | 1.4 ± 2.1 | 2.3 ± 2.5 |
| Pain interference |  |  |  |  |
| *n* | 42 | 28 | 42 | 42 |
| mean ± SD | 3.1 ± 2.9 | 1.7 ± 2.4 | 2.3 ± 3.1 | 2.9 ± 3.2 |

# eTable 7. Sensitivity Analysis: BPI scores at baseline, midpoint (C4/D1), and end of study among participants with baseline scores > 0

| **Subscale** | **Baseline** | **C4/D1** | **C4/D1 imputed** | **End of study** |
| --- | --- | --- | --- | --- |
| Pain severity |  |  |  |  |
| *n* | 34 | 21 | 34 | 34 |
| mean ± SD | 3.4 ± 1.9 | 1.3 ± 1.7 | 1.7 ± 2.2 | 2.2 ± 2.4 |
| Pain interference |  |  |  |  |
| *n* | 34 | 22 | 34 | 34 |
| mean ± SD | 3.8 ± 2.8 | 1.6 ± 2.2 | 2.3 ± 3.1 | 2.7 ± 3.1 |

#

# eFigure 4. Swimmer Plot, Sorted by Survival Time


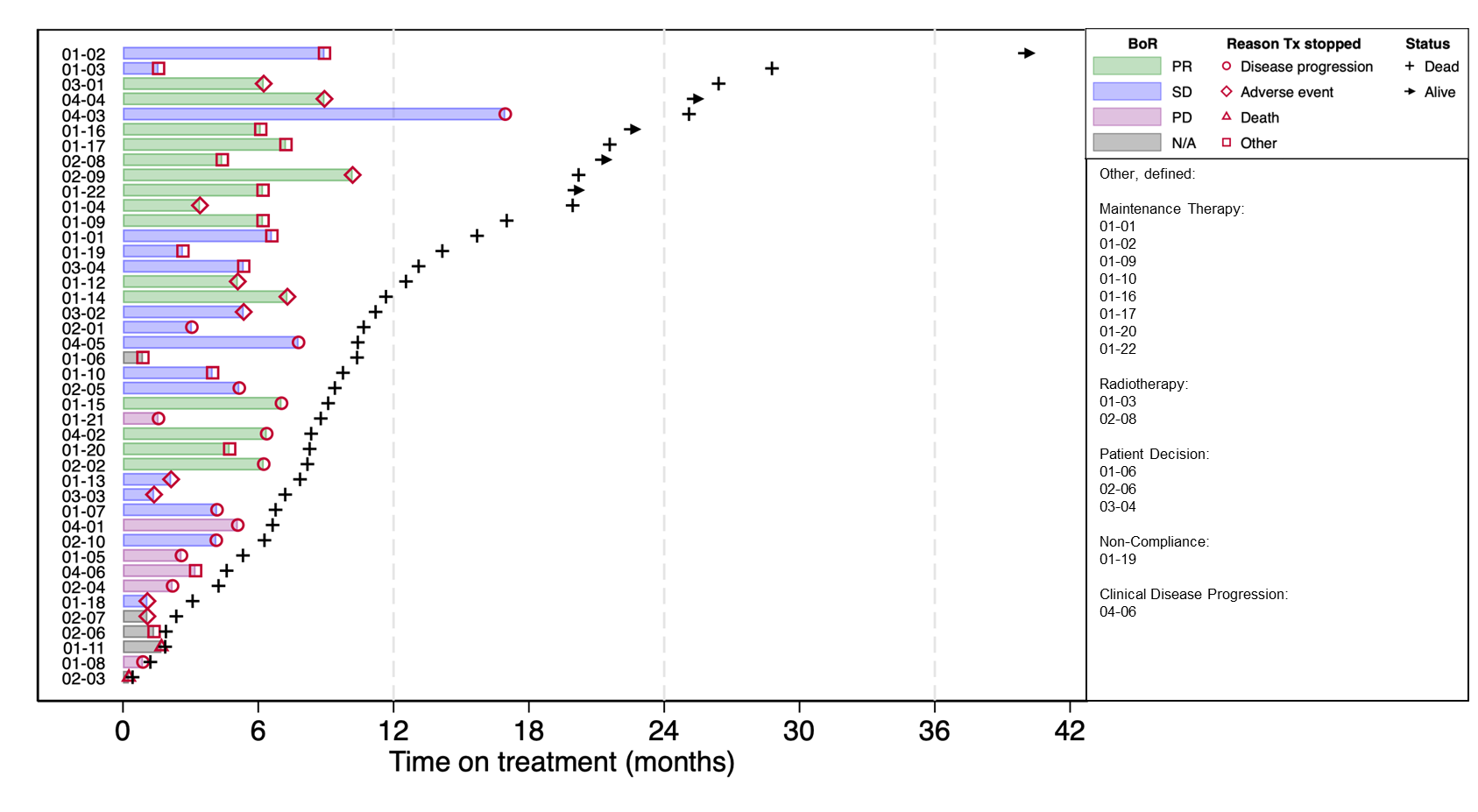


#

# eFigure 5. Swimmer Plot, Sorted by Time on Treatment


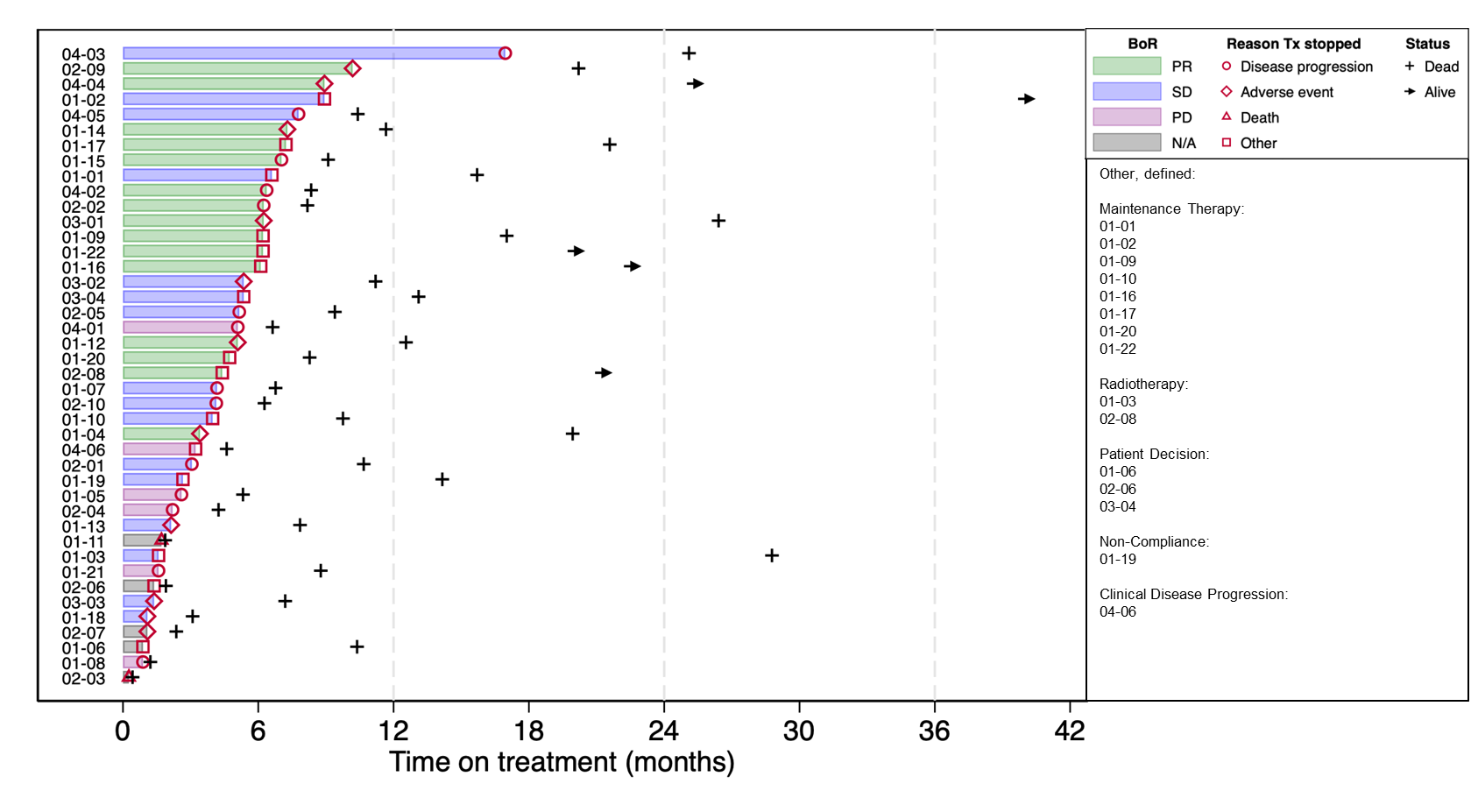


#

# eTable 8. Comparison of Phase IB/II Pilot to Phase II

|  | **Original Phase IB/II**  **NCT01893801**  **N=25** | **Phase II**  **NCT03915444**  **N=42** |
| --- | --- | --- |
| **Number of Enrolling Sites** | 3 sites | 4 sites |
| **Enrollment Distribution** | | |
| Site 1, % | 92 | 52 |
| Site 2, % | 4 | 24 |
| Site 3, % | 4 | 10 |
| Site 4, % | NA | 14 |
| **No. Evaluable for response (%)** | 24 (96) | 37 (88) |
| **Median Age, years (range)** | 65.0 (47- 79) | 66.8 (40.7-82.9) |
| > 65 years, % | 52 | 60 |
| **Sex** | | |
| Female, % | 44 | 33.3 |
| Male, % | 56 | 66.7 |
| **Race/Ethnicity** | | |
| White, % | 96 | NA |
| White Non-Hispanic, % | NA | 76.2 |
| White Hispanic, % | NA | 11.9 |
| Black, % | 0 | 7.1 |
| Asian, % | 4 | 4.8 |
| **KPS** | | |
| 100, % | 40 | 12 |
| 90, % | 48 | 43 |
| 80, % | 12 | 40 |
| 70, % | 0 | 5 |
| **Baseline elevated CA 19-9, %** | 76 | 83 |
| **Prior Cancer Related Surgery, %** | 20 | 7 |
| **Prior Adjuvant Chemo, %** | 16 | 10 |
| **OS Median, months** | 16.4 (95% CI 10.2-25.3) | 9.8 (95% CI 8.2-12.6) |
| **OS at 12 months, %** | 64 | 38 |
| **ORR (CR+PR), %** | 71 | 42 |
| **DCR (CR+PR+SD) at 9 weeks, %** | 88 | 91 |
| **Time on Treatment**  **Median, cycles (range)** | 8 (1-15) | 6 (1-23) |
| **Normalization of CA 19-9, %** | 28 | 23 |
| **Treatment related AE > 3 (most common)** | | |
| Thrombocytopenia, % | 68 | 64 |
| Neutropenia, % | 24 | 26 |
| Anemia, % | 32 | 48 |
| **Number discontinued study treatment due to related AEs (%)** | 2/25 (8) | 9/42 (21.4) |
| **SAEs (%)** | 16 SAEs in 12 pts (48) | 28 SAEs in 22 pts (52.4) |
| Related (%) | 12 (75) | 8 (29) |
| **Deaths (%)** | 3 (12) | 2 (4.8) |

# eTable 9. Study drug dosing, by trial

| **Treatment** | **Original Phase IB/II**  **NCT01893801 N = 25 median (range)** | **Phase II**  **NCT03915444**  **N=42 median (range)** | **P** |  |
| --- | --- | --- | --- | --- |
| Treatment time (weeks) | 26.9 (3.0–44.9) | 19.8 (3.0–71.0) | 0.135 |  |
| Cumulative dose for Nab-Paclitaxel (mg) | 3,396.0 (468.0–7407.5) | 2,221.5 (271.2–6,970.0) | 0.060 |  |
| Cumulative dose for Cisplatin (mg) | 7,84.0 (104.0–1482.5) | 504.0 (54.2–1,677.0) | 0.067 |  |
| Cumulative dose for Gemcitabine (mg) | 27,168.0 (3744.0–59280.0) | 17,620.0 (2,170.0–53,370.0) | 0.078 |  |
| Nab-Paclitaxel dose intensity (mg/wk) | 125.7 (70.9–165.1) | 125.6 (78.3–172.7) | 0.370 |  |
| Cisplatin dose intensity (mg/wk) | 28.9 (19.9–39.9) | 27.4 (18.1–34.7) | 0.221 |  |
| Gemcitabine dose intensity (mg/wk) | 1,037.0 (566.3–1,321.5) | 1,019.2 (678.9–1,333.3) | 0.449 |  |
| Note: P-value calculated using non-parametric Wilcoxon rank-sum test | | | | |
